# Infectious agent release and Pacific salmon exposure at Atlantic salmon farms revealed by environmental DNA

**DOI:** 10.1038/s41598-024-83250-5

**Published:** 2024-12-28

**Authors:** Arthur L. Bass, Emiliano DiCicco, Karia H. Kaukinen, Shaorong Li, Rick Johnson, John Powell, Victor Isaac, Nicola B. Dedeluk, Andrew W. Bateman, Kristina M. Miller

**Affiliations:** 1 Fisheries and Oceans Canada, Pacific Biological Station, Nanaimo, V9T 6N7 Canada; 2 Pacific Salmon Foundation, Vancouver, V6H 3V9 Canada; 3 Kwikwasut’inuxw Haxwa’mis First Nation, Alert Bay, V0N 1A0 Canada; 4 Mamalilikulla First Nation, Campbell River, V9W 8C9 Canada; 5 ‘Namgis First Nation, Alert Bay, V0N 1A0 Canada

**Keywords:** Molecular biology, Infection, Marine biology, Ecological epidemiology

## Abstract

The potential risk posed by infectious agents (IAs) associated with netpen aquaculture to wild fishes is determined based on the “release” of IAs from netpens into the environment, the “exposure” of the wild fish to those released agents, and the “consequence” for wild fish experiencing infection by those agents. Information available to characterize these three factors is often lacking, and the occurrence of transmission from aquaculture to wild fish as well as potential consequences of such transmission are difficult to observe. In this study, we utilized environmental DNA (eDNA) to characterize the release of dozens of IAs from, and exposure of Pacific salmon to, Atlantic salmon aquaculture. We combined these factors with the consequence of infection, as determined by the literature, to identify IAs that may pose a risk to wild salmon exposed to aquaculture in British Columbia, Canada. Over an 18-month period, eDNA samples were collected from seven active and four inactive netpen aquaculture sites in the Broughton Archipelago, BC. A meta-analytical mean across 22 IAs showed that the odds of IA detection at active sites was 4.3 (95% confidence interval = 2.3:8.1) times higher than at inactive sites, with 11 IAs in particular demonstrating a pattern consistent with elevated release. *Oncorhynchus tshawytscha* was the only Pacific salmon species presenting eDNA detections more likely to occur around and within active netpens relative to inactive sites. After considering the evidence of negative consequences of infection (from previous literature) in tandem with release model results, we determined that *Tenacibaculum maritimum*, *Tenacibaculum finnmarkense*, *Ichthyobodo* spp., and Piscine orthoreovirus are potential risks to Pacific salmon exposed to marine netpen aquaculture. These IAs, and others demonstrating patterns consistent with release but with insufficient prior research to evaluate the consequences of infection, require further studies that identify the factors influencing the intensity of release, the spatial extent of release around netpens, and the prevalence of infection in wild fish within known distances from netpens.

## Introduction

Domestic animal production facilities can be difficult to isolate from their surrounding ecosystems. This is especially true in the case of infectious agents (IAs), which are sometimes carried into and out of facilities by wildlife vectors or even the flow of air or water. The introduction of IAs from the surrounding environment to animals within culture facilities is well-studied due to its highly visible (and sometimes economically catastrophic) impacts on cultured animal populations. Some examples of such “wild-domestic transmission” (sometimes referred to as “spillover”, a term we avoid here due to usage ambiguity) include Brucellosis transmitted from wild ungulates to cattle in North America^1^ and avian influenza transmitted from migratory aquatic birds to poultry^2^. The converse pattern, “domestic-wild transmission”, occurs when IAs amplified in dense, captive animal populations infect wild animals in ecosystems adjacent to culture facilities. Since wild animal populations are free-roaming, the impacts of domestic-wild transmission are much more difficult to observe, and any negative impacts, as well as the associated IAs, often go unidentified.

Compared to other types of animal production facilities, netpen aquaculture faces enhanced risk for IA transfer because nets provide no barrier against the flow of water, which can both convey agents long distances^3^ and provide a matrix within which they may persist^4^. In marine aquaculture regions, IAs can be transmitted horizontally between high density culture populations that are in close proximity to one another and through movement of equipment and personnel between facilities^4–6^. While it is well established that large numbers of cultured stock are lost to wild-domestic transmission^7^, the evidence of domestic-wild transmission impacting wild aquatic populations is much more sparse due to the difficulties in observing mortality and sub-lethal effects in wild populations^8^. However, studies have demonstrated that wild fish species (and in turn, their predators) are attracted to active aquaculture sites due to abundant and consistent nutrient subsidies in the form of feed and waste, as well as the presence of a physical structure^9,10^. Once attracted, wild species may convey IAs to the cultured fish, but are also likely to be exposed to abundant macro- and microparasites amplified by unnaturally high densities of cultured fish released from predation and experiencing reduced physiological demands for survival^9^.

**Fig. 1:**
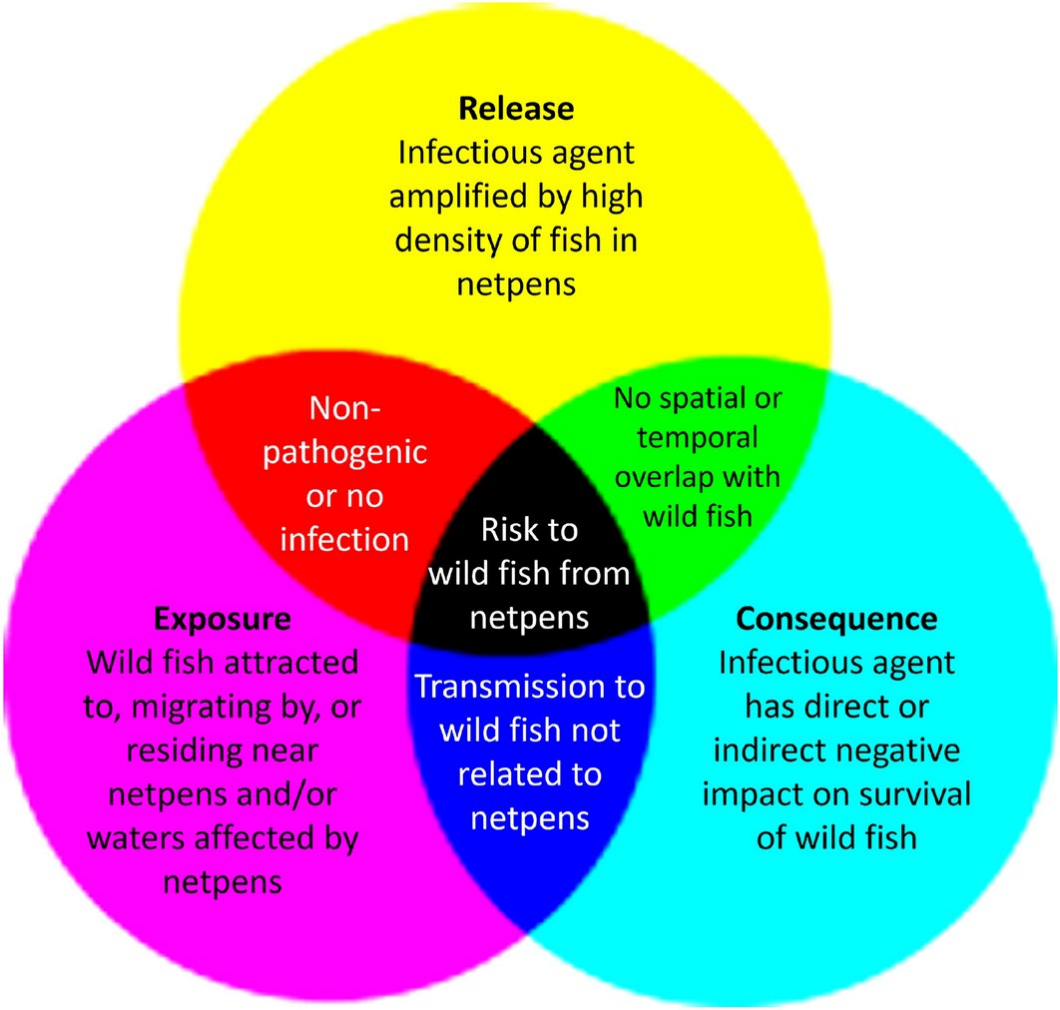
Risk to wild fish caused by infectious agent transmission from netpen aquaculture requires the intersection of release, exposure, and consequence. These three factors of risk analyses are represented in the three circles. Presence of two but not three of these factors does not indicate risk, and the outcome of two factors in the absence of the third is described in their overlaps in the figure.

In the presence of wild fish species of conservation concern and an absence of monitoring and research capable of adequately studying domestic-farm transmission from aquaculture, experts must synthesize available information to conduct risk assessments to inform regulation and management^11,12^. Considerations for whether IAs in aquaculture may pose a risk to wild fish include: whether or not cultured populations amplify IAs to concentrations beyond the baseline in the surrounding environment (referred to as “release”^11^), whether or not wild fish presence in proximity to aquaculture overlaps in space and time with release (“exposure”), and whether or not the IAs are virulent (“consequence”; Fig. 1). While consequence may be informed by previous literature, release and exposure require at least a modicum of real world data and the reliability of a risk assessment is improved as the quantity and relevance of data increases. In data-poor situations (i.e. those with elevated uncertainty) it is common to employ the “precautionary principle”, which holds that potential risk must be assumed, and protective action taken to prevent potential harm, until data satisfactory for evaluating risk are available^13^.

The increasing adoption of modern molecular technologies in fisheries science is improving our understanding of wild-domestic and domestic-wild transmission by providing novel, informative datasets. Specifically, environmental DNA (eDNA), the extraction of nucleic acids from an environmental sample such as air, water, or soil, can offer insight into release and exposure. The presence of nucleic acid fragments specific to an organism in a water sample, while not necessarily indicating that organism’s viability, does signify the recent presence of that organism in the nearby surroundings, because eDNA degrades due to ultraviolet radiation and the metabolic action of microogranisms^14,15^. In the ocean, eDNA can be quickly advected or dispersed away from the farm source due to tidal and other currents; however, the balance of advective, diffusive, and decay processes can result in equilibrium distributions of eDNA around point sources, such as netpen farms^16^. Thus, a water sample collected in a matter of minutes can provide information regarding the presence of wild fish species and IA taxa. This technology has been used in recent years to monitor or study aquaculture facilities in order to anticipate wild-domestic transmission^17^, characterize domestic release^18^, and associate IA detections with subsequent mortalities in cultured fish^19^.

In coastal British Columbia, Atlantic salmon aquaculture facilities occupy waters where multiple species of Pacific salmon (a mixture of wild and hatchery-produced fish) reside and migrate as juveniles and adults. Many of these Pacific salmon populations have experienced declines in abundance over the last three decades^20,21^, with some populations showing some of the poorest returns on record in the most recent years^20^. While the factors leading to these declines are manifold (with climate change likely playing a major role), there has been vigorous debate regarding the impact of netpen aquaculture on Pacific salmon populations. In British Columbia, as elsewhere, wild-domestic transmission is better understood than domestic-wild transmission, although several recent studies have provided evidence suggesting that several pathogens may be transmitted from netpen aquaculture to free-roaming Pacific salmon^18,22,23^.

In this study we used a novel dataset featuring eDNA detections of Pacific salmon and IAs to inform the potential release of dozens of IA taxa from netpen aquaculture sites and exposure of Pacific salmon to these releases. Environmental DNA samples were collected at active and inactive netpen sites (fallow or decommissioned) to compare the presence of both Pacific salmon (exposure) and IAs (release) between active and inactive sites. We drew information regarding the impacts of these IAs from the literature to score the consequences of infection for each of the various taxa. While a formal and complete risk assessment is beyond the scope of this study, we use some components of the risk assessment process (release, exposure, consequence) to identify agents of concern in farm-wild interactions that warrant further attention and investigation.

## Results

To determine whether contamination may have occurred at the time of eDNA collection, field blanks were collected prior to sampling at each site. Ninety-eight of 149 field blanks amplified at least one assay with a total of 208 sample/assay combinations that were positive, indicating some degree of contamination of the sampling equipment in the field. Contamination was likely due to splashing or aerosolization of ocean water during challenging (stormy, rough water) sampling conditions on an open boat deck. The amplified assays were from the most commonly detected taxa, with *Salmo salar* in 93% of positive field blanks, and *Paranucleospora theridion* (26%), and *Candidatus* Sygnamydia salmonis (23%) being the next most common. A total of 429 positive detections of pathogens or fish were nullified (coded as “NA”) due to positive detections in the field blanks from corresponding sample locations and sampling times (detections where Ct exceeded corresponding Ct in field blank). This included 227 positive detections of *S. salar* eDNA, 79 positive detections of *P. theridion*, and lower numbers (< 20) for 17 other assays.

**Fig. 2:**
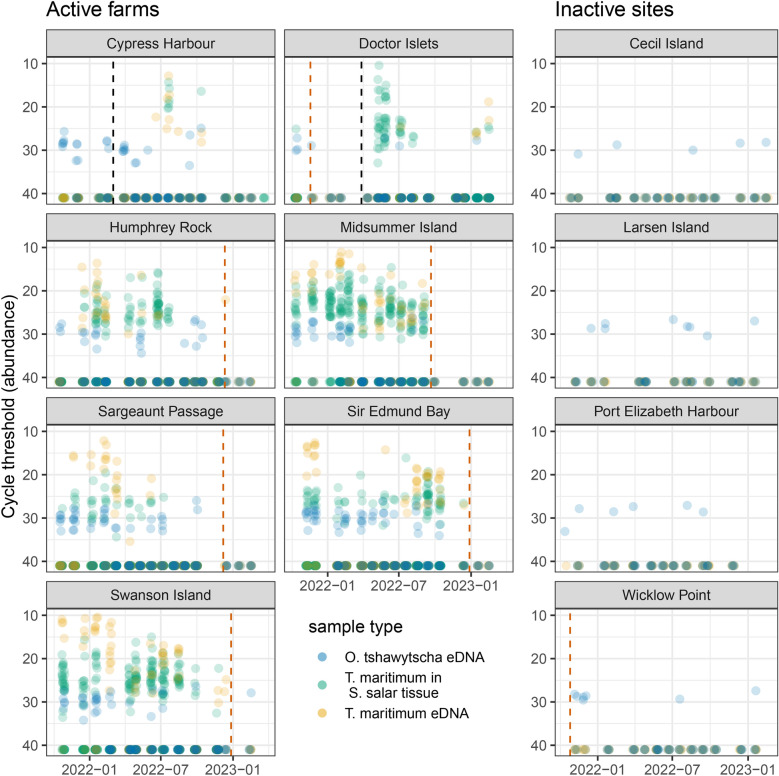
Raw data from the Broughton Archipelago reveal that *T. maritimum* DNA (orange points) was more likely to be detected in water samples from active farms compared to inactive or fallow sites, *T. maritimum* was frequently detected in *S. salar* tissues (green points), and *O. tshawytscha* eDNA (blue points) was more likely to be detected in water samples from active farms and was commonly detected in the presence of *T. maritimum*. Points exceeding 40 (max Ct) indicate samples were collected but the target nucleic acid was not detected. Black vertical dashed lines indicate *S. salar* stocking dates and red vertical dashed lines indicate harvest dates (thus farms are fallow after red lines). Note that Cypress Harbour was a broodstock facility so that occasional, but never complete, harvest occurred and Doctor Islets was the only farm to be harvested and then restocked during the study period (fallow from November 2021 to March 2022).

For some IAs, visualizing the raw data revealed stark differences between farms and inactive sites and provided evidence of both aquaculture-mediated IA release and exposure of a Pacific salmon species prior to the application of statistical analyses. A plot of *Tenacibaulum maritimum* (Fig. 2), serving as an exemplar, demonstrates that *T. maritimum* was almost never detected at inactive sites (including decommissioned and fallow farms), but was commonly detected in eDNA samples and *S. salar* tissues collected at active farms, often in the presence of *Oncorhynchus tshawytscha* eDNA. A similar pattern was observed for Atlantic salmon calicivirus (ASCV), *Caligus clemensi*, Cutthroat trout virus 2 (CTV-2), *Lepeophtheirus salmonis*, *Moritella viscosa*, Piscine orthoreovirus 1a (PRV), *Tenacibaculum dicentrarchi*, and *Tenacibaculum finnmarkense*. Plots with the same format as Fig. 2 but for all IAs, including those not modeled, are available in the supplementary material (Figures S1–S28).

### Infectious agent prevalance in *Salmo salar* and eDNA samples

Infectious agent prevalences in *S. salar* ranged from zero prevalence (*Neoparamoeba perurans*, Erythrocytic necrosis virus (ENV), Salmon Pescarenavirus 1 (SPAV-1),*Vibrio anguillarm*, and *Aliivibrio salmonicida*) to high average prevalence (78–86%) across all collections (CTV-2, *P. theridion*, and PRV; Table 1). Note that *T. finnmarkense* was not assayed in *S. salar* tissues due to the timing of assay development.

In water samples, several IAs including SPAV-1, SPAV-2, and *V. anguillarum*, were never detected. Others including ASCV, CTV-2, *Lepeophtheirus salmonis*, *Moritella viscosa*, PRV, and *T. maritimum*, were detected at very low prevalance (or not at all) at inactive sites but several times higher at active farms. In contrast, IAs including *Ca.* S. salmonis, *Flavobacterium psychrophilum*, *Ichthyophonus hoferi*, *Ichthyophthirius multifiliis*, *Kudoa thyrsites*, *Loma salmonae*, and *Piscirickettsia salmonis* showed little difference in average prevalence between active farms and inactive sites (Table 1). Comparing prevalence between eDNA samples processed with the two different lysis buffers demonstrated the considerable impact of buffer type on detectability for most assays (Table 1), although this was confounded by season.

**Table 1:** Prevalence across all Atlantic salmon and eDNA collections from active farms and fallow or decommissioned (inactive) farms in the Broughton Archipelago, BC. Blanks in the *S. salar* column indicate that these assays were not run on *S. salar* tissues. To show the potential impact of lysis buffer on prevalence, values are provided for each of the two different types (Purelink vs. Rebead)–both from eDNA samples collected at active farms. *Tenacibaculum finnmarkense* was not assayed in *S. salar* tissues.

Abbrev.	Name	Grouping	*S. salar* tissues	Active farms (both buffers)	Active farms (Purelink)	Active farms (Rebead)	Inactive sites (both buffers)
Sandlance	Ammodytes hexapterus	Marine fish		5	6.5	4.2	4.3
Herring	Clupea spp.	Marine fish		74.8	93.5	65.6	48
Anchovy	Engraulis spp.	Marine fish		59	60.6	58.2	1.1
Lamprey	Entosphenus tridentatus	Marine fish		0.1	0	0.2	0
Pollock	Gadus chalcogrammus	Marine fish		18.6	39.8	8.2	17.2
Pacifcicod	Gadus macrocephalus	Marine fish		1.1	2.6	0.4	0.4
Stickleback	Gasterosteus aculeatus	Marine fish		5.4	10	3.2	1.1
surf Smelt	Hypomesus pretiosus	Marine fish		0	0	0	0
Eulachon	Thaleichthys pacificus	Marine fish		7.4	15.6	3.4	5.7
ae_sal	Aeromonas salmonicida	Infectious agent	2.8	1.3	3	0.4	0.4
ascv	Atlantic Salmon Calicivirus	Infectious agent	52.9	14.5	36.8	3.6	1.4
ca_cl	Caligus clemensi	Infectious agent	2.7	26.4	63.2	8.4	3.2
c_b_cys	Candidatus Branchiomonas cysticola	Infectious agent	1.9	29.8	81	4.9	24.4
sch	Candidatus Syngnamydia salmonis	Infectious agent	7.7	99.9	100	99.8	99.3
ctv-2	Cutthroat Trout Virus	Infectious agent	80	19.6	46.8	6.3	0.7
env	Erythrocytic Necrosis Virus	Infectious agent	0	41	61.5	31	27.6
fa_mar	Facilispora margolisi	Infectious agent	6.4	29.4	59.7	14.6	20.8
fl_psy	Flavobacterium psychrophilum	Infectious agent	6.8	24.4	33.8	19.8	31.9
icd_spp	Ichthyobodo spp.	Infectious agent	44.9	60.6	96.1	43.2	52
icp_spp	Ichthyophonus spp.	Infectious agent	0.7	81.7	95.2	75.1	83.2
ic_mul	Ichthyophthirius multifiliis	Infectious agent	2.5	82.7	97	75.7	83.5
ku_thy	Kudoa thyrsites	Infectious agent	8.3	2.7	6.5	0.8	2.9
le_sa	Lepeophtheirus salmonis	Infectious agent	0.2	3.3	8.7	0.6	0
lo_spp	Loma spp.	Infectious agent	0.2	18.4	47.6	4.2	19
mo_vis	Moritella viscosa	Infectious agent	0.3	16.9	36.8	7.2	2.2
ne_per	Neoparamoeba perurans	Infectious agent	0	0.9	2.6	0	2.2
pa_ther	Paranucleospora theridion	Infectious agent	86	86.7	95.2	82.5	67
pa_kab	Parvicapsula kabatai	Infectious agent	0.8	4.5	10.4	1.7	2.5
pa_pse	Parvicapsula pseudobranchicola	Infectious agent	8.7	1.4	4.3	0	1.8
prv-1	Piscine Orthoreovirus	Infectious agent	78.4	17.9	36.4	8.9	1.1
pisck_sal	Piscirickettsia salmonis	Infectious agent	0.8	51.9	88.7	34	49.8
p-narnav	Putative narnavirus	Infectious agent	1.2	15.6	42.9	2.3	15.1
re_sal	Renibacterium salmoninarum	Infectious agent	0.5	1	2.2	0.4	0.4
spav-1	Salmon Pescarenavirus 1	Infectious agent	0	0	0	0	0
spav-2	Salmon Pescarenavirus 2	Infectious agent	0.2	0	0	0	0
te_dic	Tenacibaculum dicentrarchi	Infectious agent	12.9	5.5	11.3	2.7	1.1
te_fin	Tenacibaculum finnmarkense	Infectious agent		56.7	87	42	10
te_mar	Tenacibaculum maritimum	Infectious agent	20.4	27.1	36.4	22.6	0.4
vi_ang	Vibrio anguillarum	Infectious agent	0	0	0	0	0
vi_sal	Aliivibrio salmonicida	Infectious agent	0	0.3	0.9	0	0
vhsv	Viral Hemorrhagic Septicemia Virus	Infectious agent	0.1	5.5	16.5	0.2	1.1
ye_ruc	Yersinia ruckeri	Infectious agent	0.1	1.4	3	0.6	0.7
Ongo_ATP6	Oncorhynchus gorbuscha	Salmonid		2.7	2.6	2.7	2.9
Onke_ATP6	Oncorhynchus keta	Salmonid		4.7	12.6	0.8	5.4
Onki_CYTB	Oncorhynchus kisutch	Salmonid		11.5	19	7.8	9.3
Onmy_COI	Oncorhynchus mykiss	Salmonid		2.3	2.6	2.1	1.8
Onne_COIII	Oncorhynchus nerka	Salmonid		5.8	4.3	6.5	4.3
Onts_COI	Oncorhynchus tshawytscha	Salmonid		25.8	58.9	9.7	10
Sasa_COI	Salmo salar	Salmonid		95.3	98.7	93.7	43.4

### Association of Pacific salmon and other marine fish eDNA with active aquaculture

**Fig. 3:**
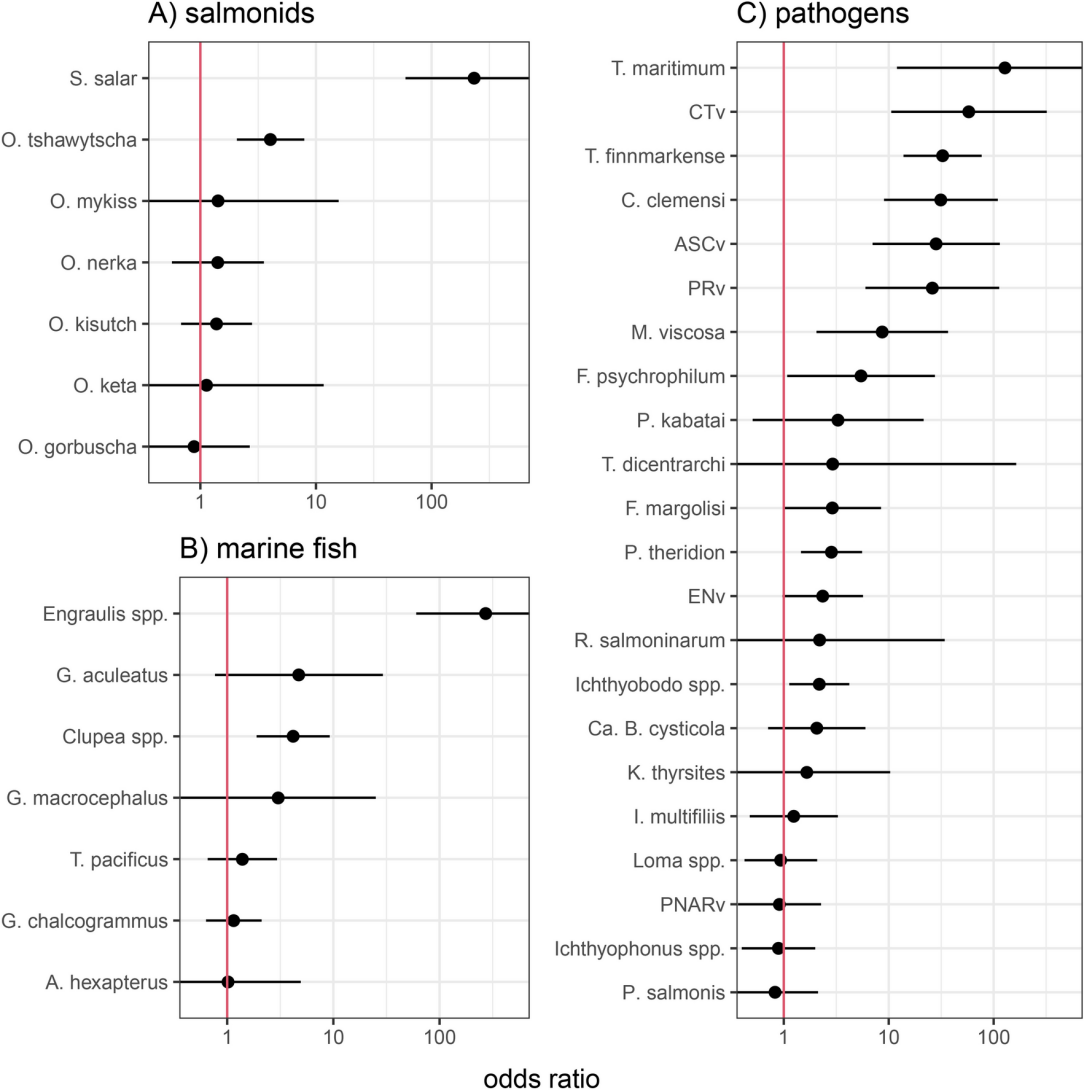
Plots A–C depict odds ratios for sample location (inactive site versus active farm) in generalized linear mixed models of eDNA presence. Points indicate the odds ratios and horizontal lines represent 95% confidence intervals around the odds ratios. An odds ratio with a 95% confidence interval exceeding 1 (red vertical line) indicates that the eDNA of that taxa is more likely to be detected at an active farm than an inactive site.

Environmental DNA from all salmon species assayed was detected over the course of the study (Table 1). *Salmo salar* eDNA was much more likely to be present in samples collected at active fish farms than those collected at inactive sites (Odds ratio = 234.0 (95% confidence interval = 59.5–921.3); Fig. 3A). *Salmo salar* eDNA was common across all samples (detected at an average prevalence of 95.3% across active farm visits and 43.4% across inactive site visits, Table 1). Of the Pacific salmon species, *O. tshawytscha* was the only species more likely to be detected at active farms than inactive sites (OR = 4.1 (2.1–7.9); Fig. 3A).

Of the non-salmonid marine fish taxa, *Hypomesus pretiosus* (surf smelt) was not detected in any water samples during the study. *Engraulis* spp. (anchovy) had the largest estimate in presence/absence models, indicating this genus was much more likely to be detected at active farms than inactive sites (OR = 273.1 (60.3–1237.1); Fig. 3B). *Clupea* spp. (herring) was the only other marine fish taxa for which eDNA was more likely to be detected at active farms relative to inactive sites (OR = 4.2 (1.9–9.2)).

### Association of infectious agent eDNA with active aquaculture

The prevalences of *Aeromonas salmonicida*, *Ca.* S. salmonis, *Lepeoptheirus salmonis*, *A. salmonicida*, *N. perurans*, *Parvicapsula pseudobranchicola*, Viral Hemorrhagic Septicemia Virus (VHSV), and *Yersinia ruckerii*, were too low for modeling and that of *Ca.* S. salmonis, which was detected in almost every sample, was too high (i.e., models would not converge). The eDNA from 11 IAs (including four bacteria, three viruses, three microparasite taxa, and one macroparasite) was more likely to be detected at active farms than inactive sites (Fig. 3C). The meta-analytic mean of the models we ran for 22 IAs was significantly positive (Figure S29), indicating that overall, the odds of detecting pathogens at an active farm were 4.3 (95% CI = 2.3–8.1) times higher than the odds of detecting pathogens at an inactive site. Linear regression comparing model estimates for IAs in this study and those generated using the data from Shea et al.^18^ indicated that estimates for IAs that were modeled using data from both studies were more similar ($\beta$ = 0.65, *p* = 0.003, $R^2$ = 0.65) than would be expected due to chance alone (Figure S30). Model estimates were on average 1.5 times higher in this study compared to Shea et al.^18^. The meta-analytic mean for the models of the ten pathogens from the study by Shea et al.^18^ that overlapped with pathogens from our study was also positive with an odds ratio of 3.3 (95% C.I. = 1.5–:7.0, Figure S29). For reference, the corresponding odds ratio from the original analysis conducted by Shea et al.^18^, which used a different modeling approach and included a total of 19 pathogens, was 2.7 (1.5–5.0)^18^.

### Infectious agent consequence scores from literature

**Fig. 4:**
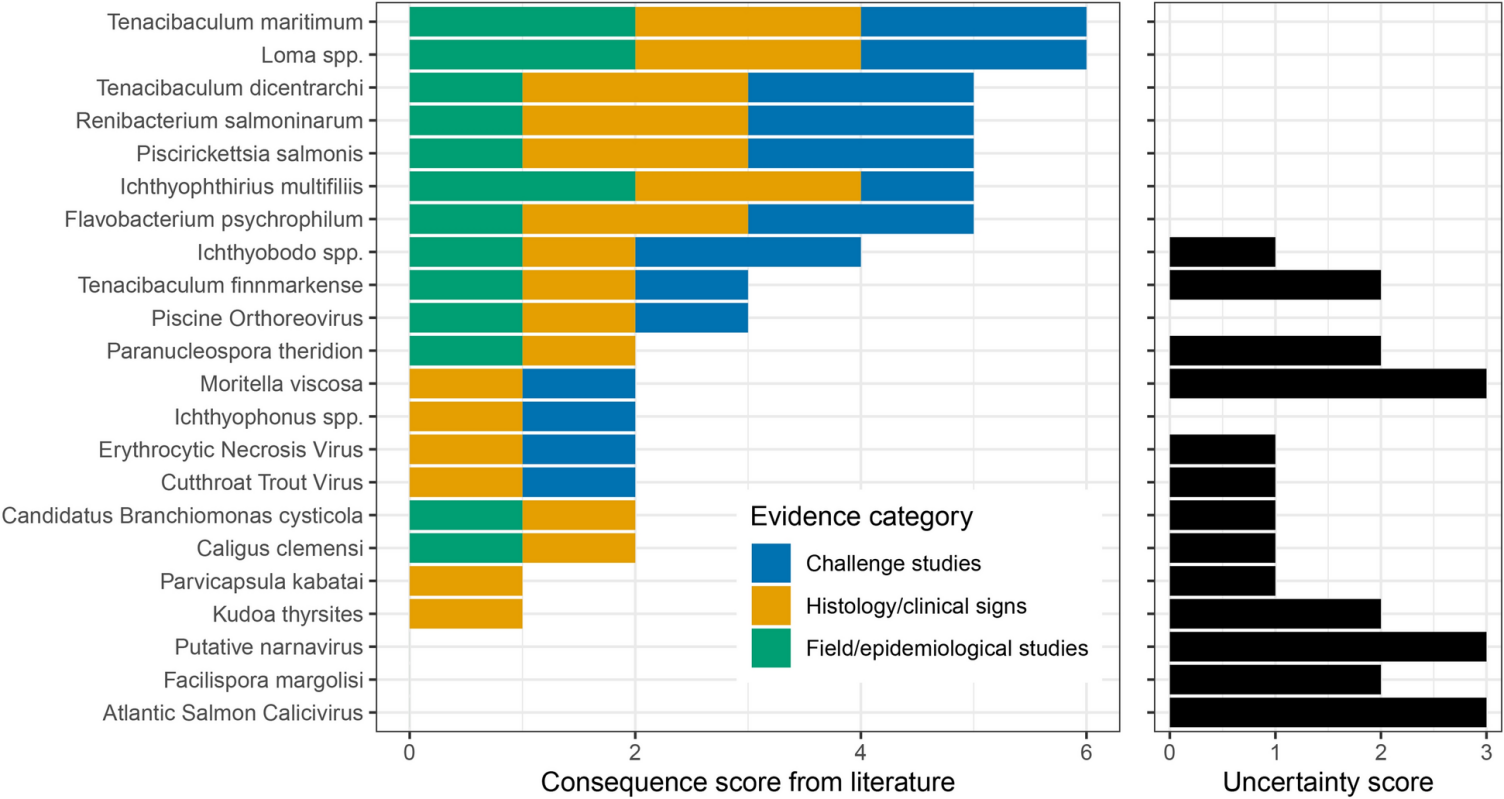
Consequence scores (left panel), and accompanying uncertainty scores (right panel), were created for each modeled infectious agent based upon published literature. The consequence score was a composite of the weight of evidence from three categories: challenge studies, histological and/or clinical signs, and field or epidemiological studies (scoring rationale and references provided in Table S1). For each category where no information was available, or only pertained to *S. salar*, a point was added to the uncertainty score.

Assigned “consequence of infection” scores and associated uncertainty based on evidence from the literature are provided in Fig. 4 (see Table S1 for score explanations and references for each IA). Thirteen of 22 agents for which we reviewed the literature lacked published studies of at least one of the categories (challenge studies, histological examinations, field epidemiology) we used to rank the evidence of consequence from infection. Putative narnavirus, in the most extreme example, has had no studies of the aforementioned categories conducted since its recent discovery^24^. Overall consequence scores ranged from zero (no evidence of impact on Pacific salmon) to six (evidence in each category of impact on at least one Pacific salmon species). Infectious agents with lower consequence scores tended to have higher uncertainty scores (Fig. 4).

### Integration of aquaculture association estimates and consequence scores

**Fig. 5:**
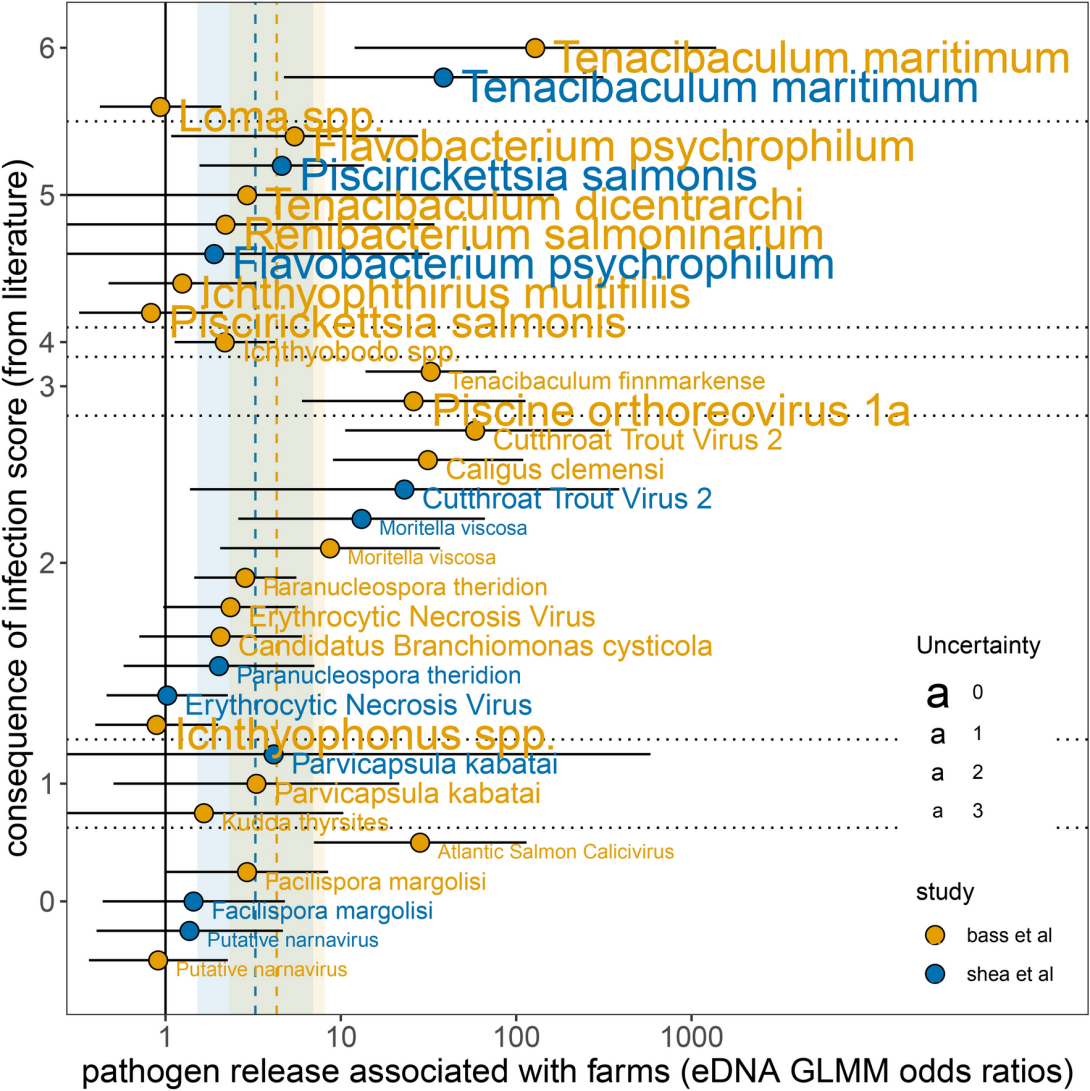
Infectious agents are positioned to demonstrate the interplay between their likelihood of release associated with aquaculture (x-axis (log-scale)—odds ratios from models in this study and a reanalysis of data from Shea et al.^18^) and the consequence of infection (y-axis—consequence scores determined from the literature; see Figure 4, Table S1). The consequence axis is not linear, IAs are ordered by consequence score (for visibility) and horizontal dotted lines indicate unit divisions between those scores. For the consequence axis, overall scores are a composite of negative impacts demonstrated from challenge studies, clinical signs and/or histopathology, and field or epidemiological studies (Table S1). The font size of the infectious agent names indicates when literature is lacking for Pacific salmon (i.e., uncertainty)—the smallest text indicates no publications featuring Pacific salmon exist for any of the aforementioned topics, and the largest text indicates at least a single study featuring Pacific salmon exists for all components of the consequence score. Error bars indicate 95% confidence intervals around the estimate. The vertical dashed lines and surrounding ribbons represent the means and 95% confidence intervals from a meta-analysis (Figure S29) of the multiple models from each study, corresponding by color.

*Tenacibaculum maritimum* was the agent with the highest consequence score and largest odds ratio from the GLMMs with which we tested for an association with active aquaculture. Further, the model estimate for *T. maritimum* generated from reanalysis of data from Shea et al.^18^ was similar to the estimate from our study (Figure 5). Other agents with consequence scores of three or greater and odds ratios not overlapping one included *F. psychrophilum*, *Ichthyobodo* spp., *T. finnmarkense*, and PRV (Fig. 5).

The remaining six pathogens that were positively associated with active farms (as determined by GLMMs) all had consequence scores below 3. However, the literature available for composing the consequence scores was incomplete for these six pathogens (Fig. 4), in contrast to those with scores above three (Fig. 5).

### Nucleic acid presence in feed samples

Duplicate samples of pelleted feed from two different companies contained nucleic acids from salmonids, other marine fish, and IAs (Figure S31). Of the salmonids, *S. salar* (20–25 Ct) and *O. nerka* (23–29 Ct) nucleic acids were present in both duplicates for both feed samples. However, subsequent sequencing of the feed samples (unpublished data) indicated that salmonid nucleic acids were found at such low quantities that they were likely the result of environmental contamination occurring prior to our collection of the feed samples. In contrast, *Clupea* spp. and *Engraulis* spp. were abundant in both PCR results (Figure S31) and the subsequent sequencing analysis (unpublished data). *Clupea* spp. nucleic acids were highly abundant in Feed 2 (12–13 Ct) where *Engraulis* spp. was approximately 10 Ct higher, but the opposite was true in Feed 1 (*Engraulis* spp. = 13–14 Ct) (Figure S31). In Feed 2, the sample with a low Ct (high abundance) for *Clupea* spp., both duplicates were positive for Erythrocytic Necrosis virus, *Ichthyophonus* spp., *K. thyrsites*, and *Loma* spp. (all 20–30 Ct, Figure S31).

### Comparison of industry sea lice counts and eDNA detections

Agreement between industry counts and eDNA samples (in terms of presence/absence) was low for *L. salmonis* (5–19%) and moderate for *C. caligus* (60–62%, Table S2). There were no positive eDNA detections for *L. salmonis* in the absence of industry count positives, but 16 for *C. clemensii* (Table S2). *Lepeophtheirus salmonis* was never detected in eDNA samples when the ReBead lysis buffer was used. The likelihood of *C. clemensii* detection in eDNA was positively correlated with industry counts of this copepod (Odds ratio = 2.8, 95% CI = 1.1–7.1, Figure S32B). There was no significant association between industry *L. salmonis* counts and either likelihood of detection or load in eDNA samples (Figure S32C, E), nor was *Caligus clemensi* industry count associated with eDNA sample load (Figure S32D). For *C. clemensi*, lysis buffer had a significant effect on likelihood of detection (OR = 21.7, 8.7–54.1, Figure S32B) and eDNA load (OR = 2.6, 1.8–3.8, Figure S32D). The binomial GLMM for *C. clemensi* predicted that when industry counts were zero and the Purelink lysis buffer was used, probability of detection would be 28–82% (Figure S32B).

## Discussion

Marine aquaculture facilities are physically complex structures featuring high trophic subsidies and densities of fish, relative to the surrounding environment. The abundance of environmental nutrient inputs in the form of pelleted food and fish feces, as well as the physical structure itself, act as attractants for a large array of marine invertebrates and fish, some of which will in turn attract larger fish species^9^. The high density of cultured fish, which are protected from predation and starvation, facilitates the amplification and release of infectious agents. Together, these aspects set the stage for a scenario wherein wild fish are attracted to netpens and then exposed to potential infection (in addition to acting as potential sources of infection for the cultured population). By measuring eDNA of Pacific salmon, marine fish, and infectious agents, we found that such an attraction/exposure scenario likely occurred for Pacific salmon in the Broughton Archipelago, particularly for *O. tschawytscha* exposed to *T. maritimum*, a marine bacterium recently demonstrated to cause high mortality in *O. tschawytscha*^25^. Although virulence has been shown to vary considerably among *T. maritimum* strains^25,26^ and the impact of BC strains of this bacterium on wild Pacific salmon is still being determined, we suggest that the evidence from this study substantiates a potential risk to wild Pacific salmon. Our results regarding IA release were statistically consistent with those of a similar study conducted independently of ours^18^, indicating that our findings were robust and the patterns we identified appear to be consistent across space and time.

We interpreted elevated levels of *O. tshawytscha* eDNA at active salmon aquaculture sites as evidence that Chinook aggregate around farms. We hypothesized that this is the result of the attraction of this species to active aquaculture sites, perhaps due to trophic subsidies or physical structure^27^. While we have not seen previous publications describing the attraction of wild Pacific salmon to netpen aquaculture sites in BC, DFO maintains multiple databases of wild fish mortalities occurring during aquaculture operations (self-reported by aquaculture companies, https://open.canada.ca/data/en/dataset/0bf04c4e-d2b0-4188-9053-08dc4a7a2b03) and *O. tshawytscha* was documented in this database on seven occasions at Broughton Archipelago farms (including several we did not monitor) during our study period. *Oncorhynchus kisutch* (coho salmon) was observed on five occasions and *O. gorbuscha* (pink salmon) on eight, both at the farms we did not monitor. The hypothesis that some species of Pacific salmon are attracted to netpen aquaculture in BC is not unprecedented as this phenomenon has been documented with other fish species in aquaculture worldwide^9,27,28^. Several studies have described aquaculture feed appearing in stomach analyses of wild fish aggregating around netpens^27^. In the Mediterranean Sea, a study on wild fish attracted to netpen sites found that feed pellets were found in 66-89% of sampled stomachs for the five most abundant species captured around netpens^29^. However, one key caveat here, relevant to how we interpret the apparent aggregation of wild Chinook salmon around farms, particularly in the context of putative exposure to IAs, is that we cannot distinguish between adult and juvenile salmonids in our eDNA detections. This is important since susceptibility to some IAs may vary with age or previous exposure. Since eDNA detections are likely to indicate that fish are (or were recently) nearby^14^, we have frequent detections of Chinook in the winter period (Fig. 2) when adults would be uncommon in this region, and we have found no accounts of adult *Oncorhynchus* spp. in close proximity to netpens but several for juvenile fish (DFO incidental catch database and Johannes et al.^30^), we suspect that *Oncorhynchus* eDNA detections are more likely to represent juvenile fish. There are few local Chinook populations in the Broughton region, which means that detections of Chinook around farms are likely to come from coastal migrants from the Salish Sea and the central coast.

In addition to *O. tshawytscha*, we found elevated likelihood of detection at active farms for other non-salmonid marine fish taxa. However, the presence of large quantities of nucleic acids from *Engraulis* spp. (anchovy) and *Clupea* spp. (herring) in feed samples we tested complicated our interpretation of these results. Combining our water sample results, feed results, and DFO’s aquaculture incidental catch database provided the most complete interpretation. The abundant *Engraulis* nucleic acids of *Engraulis* spp. detected in pelleted feed, paired with high *Engraulis* eDNA prevalence at active farms (59.0%) but low prevalence at inactive sites (1.1%), and rare occurrence in the DFO incidental catch database (only observed on one occasion in the Broughton region during our study) suggests that the *Engraulis* spp. detections in water samples were most likely from feed. In contrast, while *Clupea* spp. occurred at high abundance in the feed samples, prevalence was high in water samples from both active (74.8%) and inactive (48.0%) farms, and *Clupea pallasii* was the most frequently observed species in incidental catch records in the Broughton region (140 occasions, with up to 490 000 individuals). Thus, we suspect that our results indicating elevated probability of *Clupea* spp. detections at active farms were truly driven by the presence of *Clupea pallasii*, although this interpretation remains uncertain due to the presence of *Clupea* spp. nucleic acids in the feed. Regardless, it is not disputed that *C. pallasii* are often present at netpen aquaculture sites in BC. The presence of *C. pallasii* at netpens could result in the secondary attraction of their predators^9^, including Pacific salmon (especially Chinook salmon). In addition to potentially facilitating wild-domestic infectious agent transmission when aggregating near netpens, *C. pallasii* may also be exposed to domestic-wild transmission and subsequently act as vectors to other wild fish or other aquaculture facilities, especially as herring entrained in netpens are released live, if possible, when farmed salmon are harvested.

Both *S. salar* and *O. nerka* (sockeye) nucleic acids were detected in both feed samples we tested but were not positive in a subsequent sequencing analysis of the feed samples (unpublished data). The feed samples were collected from DFO laboratories with these salmon species on site, and samples were taken from feed bags some time after they were opened, so we assume that positive PCR detections of salmonids were due to environmental contamination. In processing landings from some Pacific salmon fisheries, salmon by-products are converted to fish meal and fish oil which may be used in feeds^31^, but we are not aware of the use of these products in aquaculture feeds used in BC. In contrast, *Clupea* and *Engraulis* spp. were detected at very low Cts (i.e. high DNA copy numbers), also detected at abundance in the sequencing analysis (unpublished data), and these species were not cultured in the laboratories where the feed samples were obtained; therefore, we are more confident that herring and anchovy constitute real components of the feeds we tested.

Our analysis identified three bacteria species from the family Flavobacteriaceae that presented a high likelihood of eDNA release from active salmon aquaculture sites and high consequences of infection for Pacific salmon: *F. psychrophilum*, *T. maritimum*, and *T. finnmarkense*. *Flavobacterium psychrophilum* is well know from freshwater aquaculture, including Pacific salmon hatcheries^32^. While there is evidence that *F. psychrophilum* can persist in brackish water (6 ppt)^33^, we have seen no reports of transmission of *F. psychrophilum* or associated clinical disease in marine aquaculture or marine resident wild salmonids in BC. However, marine molecular detections of this bacterium have been observed in Pacific salmon^34,35^ and a geostatistical analysis indicated that early marine *O. tshawytscha* sampled closer to active netpen aquaculture were more likely to be positive for *F. psychrophilum*^36^. Nevertheless, molecular detection does not imply disease, and to confirm risk to Pacific salmon from *F. psychrophilum* in the marine environment, evidence of transmission in this environment is required.

In contrast to *F. psychrophilum*, the pathogenicity of both *T. maritimum* and *T. finnmarkense* has been well established for *S. salar* in the marine environment^37^. While clinical disease in Pacific salmon from *T. maritimum* has not been documented in BC per se, this cosmopolitan bacterium has been linked to mortality and clinical disease in Pacific salmon species elsewhere (*O. mykiss* in Chile^38^, in challenge studies using *O. mykiss* in Australia^39^ and *O. tshawytscha* in New Zealand^25^, in netpen *O. tshawytscha* in California^40^ (lesions), and in Alaska^41^). Virulence of *T. maritimum* varies among strains^25,26^, but strains that cause high mortality in *S. salar* are certainly present in BC aquaculture, where (excluding sea lice) *T. maritimum* is the infectious agent most often associated with pathogen-mediated mortality^42^. Therefore, while our qPCR assay did not discriminate among strains of *T. maritimum*, there is a substantial probability that virulent strains were represented in our results. In a correlative study, Bass et al.^43^ found negative associations between *T. maritimum* prevalence and load and population-level survival and body condition for *O. tshawytscha* and *O. kisutch*. Like *T. maritimum*, *T. finnmarkense* has been little studied in *Oncorhynchus* spp. in BC, but clinical disease has been documented in *O. mykiss* and *O. kisutch* in Chile^44^. *Tenacibaculum dicentrarchi* did not appear elevated at active farms in our study, but low prevalences across all samples led to high uncertainty in model estimates for this bacterium (Fig. 3C), and it was present in *S. salar* tissues at farms, although at half the average prevalence of *T. maritimum*. This is in contrast with the geographically overlapping work of Nowlan et al.^45^, where *T. dicentrarchi* typically occurred at higher levels than *T. maritimum* at two Broughton Archipelago farms and was considered the primary agent in Tenacibaculosis outbreaks. Despite several recent studies that have greatly expanded our understanding of the distribution and pathogenicity of *Tenacibaculum* spp. in BC and for *S. salar*^37,45,46^, there is a lack of studies addressing potential impacts to *Oncorhynchus* spp. Our study, in which *T. maritimum* provided the most striking intersection of release, exposure, and consequence across all pathogens tested, interpreted in concert with other recent works^23,25,43,45,47^, indicates that *T. maritimum* release from farms poses a potential risk for wild Chinook salmon in regions with active aquaculture.

Several viruses, including PRV, CTV-2, and ASCV, had positive associations with active farms, indicating elevated release. Piscine orthoreovirus 1a had the highest consequence score of these three viruses, principally because it has been studied more than the others. Recent research has found that likelihood of infection with PRV increases as the distance from active fish farms decreases^22,36^ and this has also been noted from non-statistical observations^48,49^. Other studies conducted in BC have indicated that PRV infection prevalence was negatively correlated with population-level survival for *O. tshawytscha*^43^ and PRV infection was associated with underweight *O. tshawytscha* and *O. kisutch*^43,49^. Wang et al.^50^ found that PRV-infected early marine juvenile *O. tshawytscha* had gene expression patterns consistent with a viral disease response and histopathological evidence of jaundice/anemia, a disease that has been associated with PRV infection on farms^51^, with similar disease manifestations in Pacific salmon and trout caused by other strains of PRV detected outside BC^52,53^. In contrast, laboratory challenge studies have led some researchers to conclude that PRV is not a risk to Pacific salmon^54,55^. The incongruous findings from laboratory challenge studies and observational field studies may indicate that other factors (e.g., temperature, predation) mediate PRV-related impacts. Taken together, the mounting evidence herein and across several published studies points to PRV posing the most impactful viral transmission risk posed to wild salmon from farms in BC.

Neither CTV-2 nor ASCV have been studied extensively in *Oncorhynchus* spp., and thus high consequence scores from the literature were not possible. Although ASCV has been shown capable of establishing systemic infection in *S. salar*^56^, there is no evidence that it causes clinical disease in that species and we found no studies focusing on the impacts of ASCV on *Oncorhynchus* spp. Challenge studies conducted on *O. gorbuscha*, *O. tshawytscha*, and *O. nerka* found that CTV-2 could be naturally transmitted from *S. salar* to *O. tshawytscha*^57^. Histological changes were found in the heart and kidney but the authors were unable to conclusively link this to infection with CTV-2 because control fish were not histopathologically assessed^57^. Nevertheless, the study by Long et al.^57^ demonstrated that in cell culture, CTV-2 had cytopathic effects on *O. tshawytscha* cells, viral loads persisted at higher levels in *O. tshawytscha* relative to other *Oncorhynchus* spp., and that endocarditis and intratubular protein casts in the kidney were more likely to occur in PCR-positive than PCR-negative *O. tschawytscha*. In-situ hybridization of CTV-2 in Atlantic salmon revealed particularly high level detections in the brain^24^, a tissue that was not examined in the *Oncorhynchus* challenge study^57^. Moreover, CTV-2 has been detected in both farmed and wild Chinook salmon, reaching prevalence on farms upwards of 12%^24^. Thus, further investigation of this virus in Pacific salmon is warranted and, given its strong release score, it should be considered a potential risk until more is known.

Two microparasite taxa, *Ichthyobodo* spp. and *Paranucleospora theridion* had positive associations with active farms. *Ichthyobodo necator* or *I. salmonis* are the species of this genus most likely to impact salmonids, but our genus-specific assay for *Ichthyobodo* cannot discriminate between these two species or others in the genus. Using the same assay as ours, Deeg et al.^58^ detected *Ichthyobodo* at prevalences of 14–30% in *O. keta, O. gorbuscha* and *O. kisutch* collected in the Gulf of Alaska, indicating that this genus of protozoan parasites may infect *Oncorhynchus* spp. in our region. Kent et al.^59^ observed heavy *Ichthyobodo* infections associated with gill damage in netpen *O. tshawytscha* in Sechelt, BC. Compared to BC, *Ichthyobodo* has been more thoroughly studied in Japan, where both *I. necator* and *I. salmonis* have been experimentally shown to cause high mortality in *O. keta*^60,61^. Given the high release score for *Ichthyobodo* in our analysis, the noteworthy lack of study on how this prevalent parasite genus impacts BC *Oncorhynchus* spp. populations, and its pathogenicity in other regions (which resulted in a relatively high consequence score in our analysis), we urge more research in the near future. *Paranucleospora theridion* is less understood than *Ichthyobodo* and its impact on physiology and the factors which regulate infections are understudied globally^62^. There is evidence that this marine microsporidian parasite contributes to gill disease^62^ and that infections are enhanced by elevated water temperatures^36,63^, suggesting it could become more impactful as ocean temperatures rise.

Based on the extensive body of literature associating sea lice presence and abundance with active netpen aquaculture worldwide and in our study area^64,65^, we were surprised to find very low prevalence of *Lepeophtheirus salmonis* (salmon louse) eDNA (too low to model). In contrast, the other sea louse assayed in our study, *Caligus clemensi*, showed a moderate prevalence and was more likely to be detected at active farms than inactive sites. The lower eDNA detection prevalence of *L. salmonis* relative to *C. clemensi* was unexpected given that the DFO industry sea lice count database indicated that *L. salmonis* was more commonly observed than *C. clemensi*. One factor that may help to explain this difference is the fact that *C. clemensi* are considered to be generalists but commonly attach to herring (indeed, one of their common names is “herring lice”), which we showed to be associated with active salmon farms. A three times higher rate of agreement for *C. clemensi* relative to *L. salmonis*, in terms of presence/absence between industry counts and eDNA detections, suggests that some aspect of our eDNA collection or laboratory analysis is better suited to the former species. Alternatively, the physiology or life cycle of *C. clemensi* somehow results in a greater rate of nucleic acid shedding or persistence in the environment. There is some evidence that crustaceans shed nucleic acids at low rates due to their hardened exoskeleton^66^, but both ectoparasite species in our study are copepod crustaceans. However, this could explain why eDNA concentrations of neither sea louse species was correlated with industry counts. Other researchers have reported unexpected dissimilarities between sea louse detections in eDNA and manual sea louse counts^17,67^ and this has been the case for other IAs as well^15^. Whatever the case, the low detection rate of *L. salmonis* in the presence of positive manual counts suggests that our study does not properly characterize the release of *L. salmonis* from active netpens and therefore we make no inference regarding the risk associated with this species. For *C. clemensi*, the presence (but not eDNA concentration) of which was positively correlated with industry counts, our results indicate that *Oncorhynchus* spp. are more likely to encounter this ectoparasite around active farms than inactive sites. We note also that, in addition to transmission via infective copepodid larvae, *C. clemensi* are more likely to transfer between hosts as adults and may be more common in the water column around infested farms. Few studies have investigated the potential impacts of *C. clemensi* on *Oncorhynchus* spp. but there is evidence of reduced growth^68^ and foraging ability^69^ for *O. nerka* infected with *C. clemensi*.

Records from one of the aquaculture companies in the study indicated that *S. salar* were vaccinated against *Aliivibrio salmonicida*, *Vibrio anguillarum*, *Aeromonas salmonicida*, *Moritella viscosa*, and *Renibacterium salmoninarum*. Indeed all of these infectious agents were detected not at all or at very low prevalence in *S. salar* tissues, and only *M. viscosa* and *R. salmoninarum* were detected at sufficient prevalence in eDNA to be statistically analysed. *Moritella viscosa* eDNA was more likely to be detected at active farms than inactive sites, which was surprising given its low prevalence in *S. salar* tissues. One potential explanation of this phenomenon is that other fish species attracted to netpens could be bringing this bacterium into the vicinity. Similarly, shedding from *C. pallasii* or other fish species attracted to netpens might also explain the elevated presence of erythrocytic necrosis virus (ENV) in eDNA collected at active farms. Although it was completely absent from *S. salar* tissues, ENV was more likely to be detected at active farms than at inactive sites (confidence intervals marginally overlapped zero, Fig. 3C) and *C. pallasii* is a well-known carrier of this virus^70^. Although our literature search indicated a low consequence score for ENV, the elevated detections despite absence of the virus in *S. salar* tissues illustrates a potential scenario where the attraction of one species (*C. pallasii*) results in pathogen exposure for another (e.g., *Onchorhyncus* spp.), a phenomenon described previously^18,71^. ENV was detected in both of the feed samples we tested (stronger detection in the sample with higher levels of *C. pallasii*) and therefore feeds administered at farms could be another source of elevated detection frequency.

Risk assessments regarding wild sockeye salmon and specific aquaculture interactions were conducted in British Columbia in 2019. These assessments were led by DFO to address the Cohen Commission recommendation that salmon aquaculture facilities be removed from the Discovery Islands unless they were demonstrated to pose minimal risk to Fraser River *O. nerka*^72^. The nine assessments covered risk from IHNV, *A. salmonicida*, *P. salmonis*, *R. salmoninarum*, *Y. ruckerii*, PRV, *T. maritimum*, *M. viscosa*, and VHSV (https://www.dfo-mpo.gc.ca/cohen/iles-discovery-islands-eng.html) potentially transferred from Discovery Island salmon farms. While our study included assays for all of these pathogen taxa aside from IHNV, we detected several at prevalences too low for statistical analysis (*A. salmonicida*, *Y. ruckerii*, VHSV), and found that two others did not show differences in detection prevalance between active farms and inactive sites (*R. salmoninarum*, *P. salmonis* - although reanalyzed data from Shea et al.^18^ indicated that *P. salmonis* was more likely to be detected at active farms). For the remaining three (PRV, *T. maritimum*, and *M. viscosa*) we found that release was elevated at active farms, findings consistent with the release assessment for these pathogens in the DFO risk assessments^73–75^. However, our interpretations concerning the likelihood of exposure and consequences of infection for these pathogens differ from the conclusions in the DFO assessments. This is partly due to the fact that, for the exposure element, the DFO risk assessments solely focused on Fraser River *O. nerka*, while we assayed all *Oncorhynchus* spp. occurring in our region and found that *O. tshawytscha* were more likely to be detected at active farms than inactive sites. For *M. viscosa*, the DFO risk assessment stated that this bacterium does not infect *S. salar* between the months of May and October, and therefore would not be amplified and released during the *O. nerka* migration period^74^. In contrast, we often detected *M. viscosa* eDNA at farm sites concurrently with *O. tshawytscha* eDNA, and the potential impacts of this bacterium on that or other *Oncorhynchus* spp. have not been studied. For PRV, consequences of infection were considered negligible in the DFO risk assessment^73^, but several studies published afterwards^43,50^, and others published prior^51,76,77^, indicate potential for consequences for *O. tshawytscha* and *O. nerka* that should be considered underexamined risks when operating under the precautionary principle. Infection with *T. maritimum* was considered unlikely to occur for Fraser River *O. nerka* by the DFO risk assessment^75^, but there have been no actual laboratory studies performed on sockeye salmon to determine their susceptibility to infection by this bacterium. Clinical disease from *T. maritimum* has, however, been observed in *O. tshawytscha* outside of BC^25,40,41^ and clinical disease and mortality in adult *O. tshawytscha* were recently observed in BC from a closely related species, *T. dicentrarchi*^47^.

Comparing our study and the DFO risk assessments for these latter three pathogens illustrates potential impacts on conclusions drawn from risk assessments due to the assessment’s scope (considering a single host species for exposure versus six species) and the availability of literature informing potential consequences of infection. Incomplete information from the scientific literature or regional field studies contributes to uncertainty in risk assessments, as illustrated by the case of *T. maritimum*. Challenge studies have not yet been conducted to determine whether BC strains of this bacterium can cause disease in BC wild salmon (although challenge trials are currently underway at DFO). In this context, the causal evidence of harm to Pacific salmon in BC remains, technically, outstanding. That said, the features of risk - and potential to mitigate that risk—will vary from context to context: region to region, species to species, and so on. Even when causality is known and risk—broadly defined—exists, environmental factors (e.g. temperature and salinity), pathogen factors (e.g. strain and dosage), and host factors (e.g. species, nutritional condition, predation context, immune state) all constitute “component causes”^78^, which may or may not create the conditions for an etiological agent to cause disease. Although we have not delved into all of the specifics - or component causes - required for a full-blown risk assessment, we have demonstrated necessary (if not sufficient) conditions for risk to wild Pacific salmon, particularly in the context of the precautionary principal. In a framework like the one we used here (a common framework based on the three key elements of release, exposure, and consequence), further information is much more likely to lead to more evidence of pathogenicity and risk of release, rather than less (since the default is minimal risk). Thus, our conclusions regarding which IAs may pose potential risks to Pacific salmon are likely conservative, particularly when we consider agents with strong evidence of release but insufficient scientific literature to accurately assess consequence, including *M. viscosa*, *T. finnmarkense*, *P. theridion*, CTV-2, *C. clemensi*, and ASCV.

Our definition of exposure in this study is based on elevated detections of wild fish eDNA within and around netpen facilities (within 50 m), and is thus more narrowly defined than what may truly be important ecologically. This is because our study was not designed to determine the spatial extent of IA release around active netpen facilities. Shea et al.^16^ found that *S. salar* eDNA could be detected up to 3.7 km from active farms, but the distance over which infectious agents remain viable is likely to vary across taxa^15^. Due to the biology of different IAs and the hydrodynamics around facilities, the highest infection pressure may not always occur in the immediate surroundings of an active farm^79^. Therefore, any individual Pacific salmon (not just *O. tshawytscha*) that migrates through areas in our study region where infection pressures are elevated could be exposed to agents released from netpens. Future studies, including eDNA collections, should feature an experimental design capable of determining the spatial extent of IA release from active farms, perhaps informed by biophysical models^80^.

A confirmation of exposure superior to co-occurrence of IA and host eDNA would be the observation of infections, above any background rate, in wild salmon collected in areas known to experience elevated release of IAs from netpen aquaculture (caged sentinels have been used to this end in the past^81^). Detection in eDNA cannot confirm infection of wild salmonid hosts^15^. The co-occurence of IA and host eDNA that we have interpreted here would also be consistent with scenarios in which wild hosts are exposed to IAs released from aquaculture but do not become infected. From the eDNA data we are unable to determine the precise location of Pacific salmon, the viability of a detected IA, or the infection pressure at a given location; therefore, we are unable to determine the likelihood of infection based on previously established challenge models (which are scarce for Pacific salmon for many of the IAs we assayed). However, because removal of free eDNA from the environment is fast and IA genetic material is relatively rare in the environment, the chances of detecting dead or extracellular nucleic acids is expected to be very low and thus detection in aquatic systems is highly likely to represent a viable life cycle stage^14,15^. Furthermore, the well-studied phenomenon of waterborne transmission of IAs between aquaculture sites that are kilometers apart (whether through free-ranging fish vectors or waterborne transmission)^3,79^ suggests that opportunities for transmission to susceptible wild fish around active farms should be high. Finally, previous studies have shown that fish collected at varying distances from netpen aquaculture were more likely to be infected with IAs and macroparasites as the distance from aquaculture decreased^9,22,36,49,64,65,77,82^. To resolve the data gap between co-occurrence of eDNA and potential infection, studies sampling wild salmon at varying distances from aquaculture, perhaps via nimble methods like “micro-trolling”^83^ or more experimentally controlled methods like caged sentinels^81^, are required and should be paired with eDNA collections. Challenge studies featuring Pacific salmon exposed to varying doses of IAs (and concurrent eDNA collection) would also aid in our interpretation of the risk represented by IA concentrations measured around netpen aquaculture via eDNA sampling. Until such laboratory challenge and field exposure studies are conducted, the combination of field data that characterize the risk of IA release from netpen aquaculture (as found in this study) coupled with consequence information from previous literature is the best means for evaluating the risk posed by netpen aquaculture to wild Pacific salmon. The precautionary principle suggests that the lack of laboratory challenge and field exposure studies (i.e. insufficient data for conclusively determining risk) does not warrant an assumption of minimal impact for wild fish.

The spatial (Broughton Archipelago region) and temporal (18 months) extents of our study may limit the applicability of its results. Variable abiotic and biotic factors among aquaculture regions in BC (and more broadly) are likely to influence the assemblages of salmonids and marine fish as well as the diversity of infectious agents^35,36,42^. Interannual variability can result in outbreak or absence of some IAs over time, as well as strong shifts in salmonid abundance (e.g., between the distinct odd versus even calendar-year *O. gorbuscha* populations). Nevertheless, we found striking similarities between our IA results and those of Shea et al.^18^, who sampled from a broader geographical region (Broughton Archipelago and Discovery Islands) and across three years prior to our study (2016-2018), indicating that many of our results are likely not exclusive to the spatiotemporal extent of our study. Furthermore, while we cannot use our results to generalize about what IAs might be problematic along the entire BC coast, we can use them to prioritize IAs for regulatory consideration, monitoring, and future research. Infectious agents that did not appear to be at a high risk of exposure for wild Pacific salmon in this study could be important elsewhere or under other (perhaps interannually varying) conditions^15^.

Potential sources of bias in our study include those that are specific to our field and laboratory methods, and those specific to the biology of the system that may favor detection of some IAs over others. Methodological biases may include the depth at which samples were collected, the volume of water filtered, filter size and type, and various aspects of the extraction procedure. If eDNA is stratified in the water column, our data might not fully characterize what fish species are attracted to farms because fish abundance and diversity have been shown to vary with depth around netpens^27^. The fact that we had to include the lysis buffer type in our models demonstrates the strong effect of this aspect of the extraction process and we recommend against such a switch mid-study, although it was necessary in our case due to limited availability of the Invitrogen lysis buffer at the time, in part due to the COVID-19 pandemic. Despite methodological differences between our study and Shea et al.^18^, including sampling at different depths, using different filters and filtering methods, and using different extraction techniques, we found very similar results, suggesting the biological patterns we observed were robust to variation in methods. However, Shea et al.^18^ almost never detected PRV, an RNA virus that we detected at moderate prevalence, and this was likely because those authors did not specifically extract RNA from filtrate material.

Agent-specific factors potentially biasing our characterisation of IA release include stability in seawater, variation in how particles disperse in the water column (e.g., parasite spores versus virus particles), and variability in life cycles that influence how IA eDNA is shed from hosts (free versus bound in faeces, mucous, urine, etc). By bias, here we mean among-IA differences in our ability to quantify release. For example, our analysis herein did not consider free viruses—those not bound in shed cellular material that could be captured on a filter. Given that eDNA shedding and decay rates vary with different animal forms^66^, it is likely that variability in eDNA persistence also exists within the IA taxa that constitute our assay panel (e.g., RNA viruses versus multicellular parasites). Pathogens that are transmitted between hosts by vectors and infect internal organs could be more difficult to detect than those transmitted by the fecal-oral route through the water column. Such sources of bias are likely to result in an underestimation of the presence of some infectious agents, relative to others. It is possible that if some taxa are underrepresented due to our methodology, we could be misinterpreting their potential for amplification and release associated with aquaculture.

The remaining aquaculture facilities in the Broughton Archipelago (those monitored in this study) were decommissioned in 2023, as decided by the First Nations included in the BATI agreement. However, the IAs identified as potential risks to wild salmon in this study, as well as the use of eDNA collections to inform risk assessments around domestic-wild pathogen transmission, may be applied to other regions in Canada where potential wild-aquaculture interactions exist. With results exceptionally consistent with those of a previous study^18^, despite methodological variations, our study presents robust evidence of IA release from marine netpen aquaculture. These data and others collected similarly should be considered as important elements to address data and knowledge gaps regarding domestic-wild transmission in future risk assessments.

Now that *T. maritimum*, *T. finnmarkense*, *Ichthyobodo*, and PRV have been identified as IAs that are released from active farms in BC and have known disease potential in *Oncorhynchus* spp., an important next step is to determine the environmental, biological, and operational factors that impact their shedding rates. Such a mechanistic understanding can be used to inform progressive regulations of industry to reduce impacts of these agents on wild salmon. As a hypothetical example, *T. maritimum* might be released from farms at elevated levels: on a seasonal pattern or when water temperatures are high, when fish are stressed by co-infections or in otherwise poor condition, or following farm operations that lead to acute stress such as mechanical delousing. This information would serve as the first biological input, the rate of IA particle release from a given location, into any prospective biophysical model^80^. Controlled laboratory studies could provide necessary information for other inputs, including the persistence of *T. maritimum* at various temperatures and salinities and the infection pressure required to cause infection^80^. With these parameters, biophysical models could predict the dispersal of *T. maritimum* around netpen aquaculture networks and collections of eDNA and wild fish could be used to ground truth model predictions^80^.

Regardless of the answers that future studies of pathogen release and dispersal will provide, no single approach is likely to provide comprehensive insight to the question of risk. For example, questions a biophysical dispersal model alone cannot answer are those that relate to the potentially infected hosts. Inter-species variability in susceptibility to infections, wild host migration details (in space and time), the complicating effects of cummulative stress, and indirect effects of sublethal infections will all factor into the true risk that any IA release creates to wild salmon. Judiciously combining all the relevant features, while also accounting for uncertainty, will be required to holistically gauge risk.

## Methods

### Methods overview

**Fig. 6:**
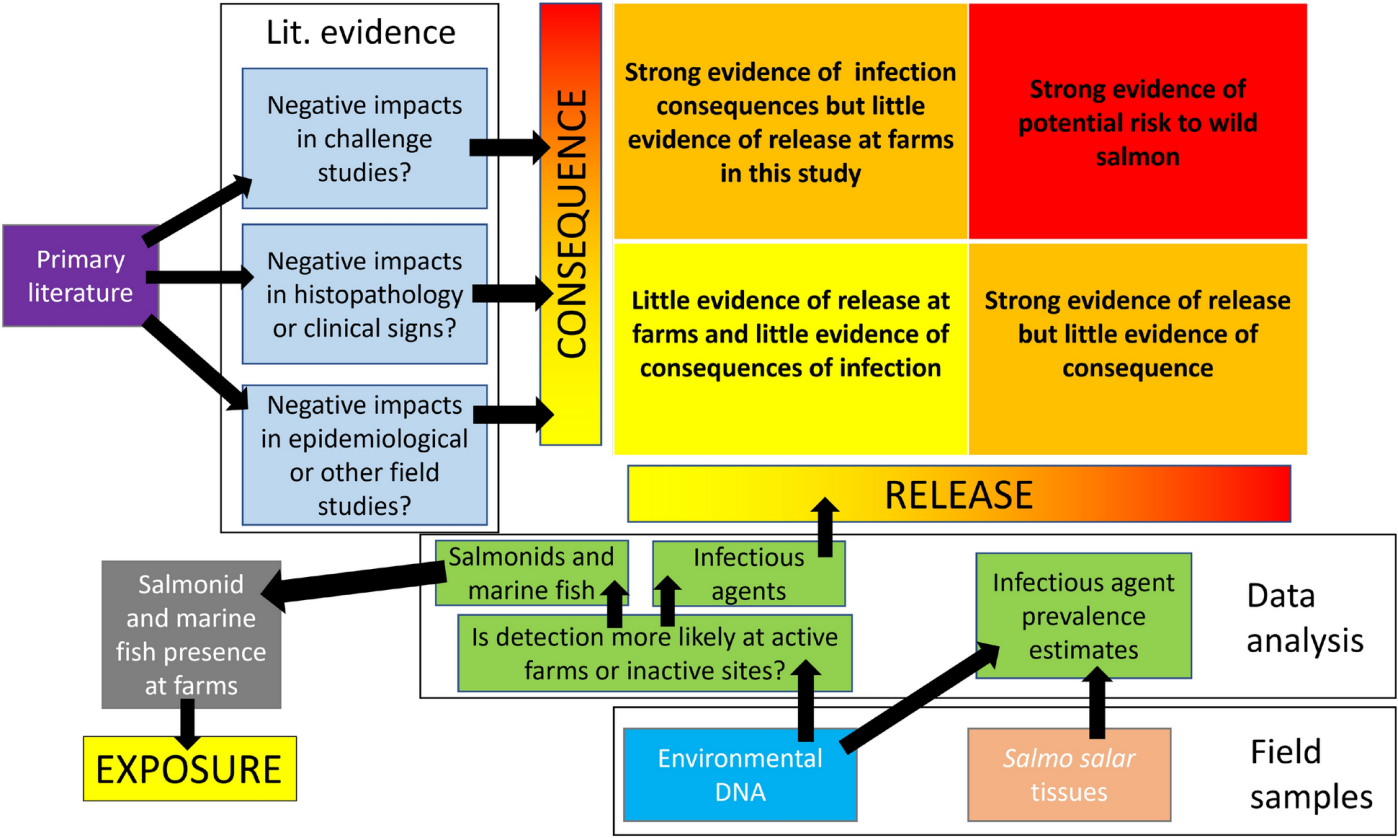
Schematic outlining the study design. Environmental DNA collections were used to characterize infectious agent release at active farms and exposure of wild fish. Evidence from the literature was used to develop agent-specific consequence scores.

To approximate whether any IAs found in Atlantic salmon netpen aquaculture might pose a risk to wild Pacific salmon, we considered the likelihood of release and exposure as evidenced by eDNA detections of IAs and their hosts, coupled with the consequences of infection as determined by evidence from previous literature (Fig. 6). eDNA samples were collected over 18 months from seven active and 4 inactive (decommissioned or fallowed) netpen aquaculture sites and analyzed by qPCR for the presence of salmonids, marine fishes, and IAs (Table 1). To test for evidence of IA release, we modeled whether or not IAs were more likely to occur at active farms versus inactive sites. For exposure, we used the same model structure to determine which Pacific salmon species were more likely to be detected at active farms and thus more likely to be exposed (to agents that showed evidence of release). For consequences of infection we considered whether evidence existed in the form of negative impacts determined from challenge studies, histopathological and/or clinical evidence of disease, or evidence of population level impacts from field studies. Evidence from the literature was converted to qualitative scores. These scores and model estimates were then considered together to identify agents of concern (high probability of release and high consequence score, upper right of Fig. 6), particularly for Pacific salmon species with high exposure (Fig. 6).

### Study area

**Fig. 7:**
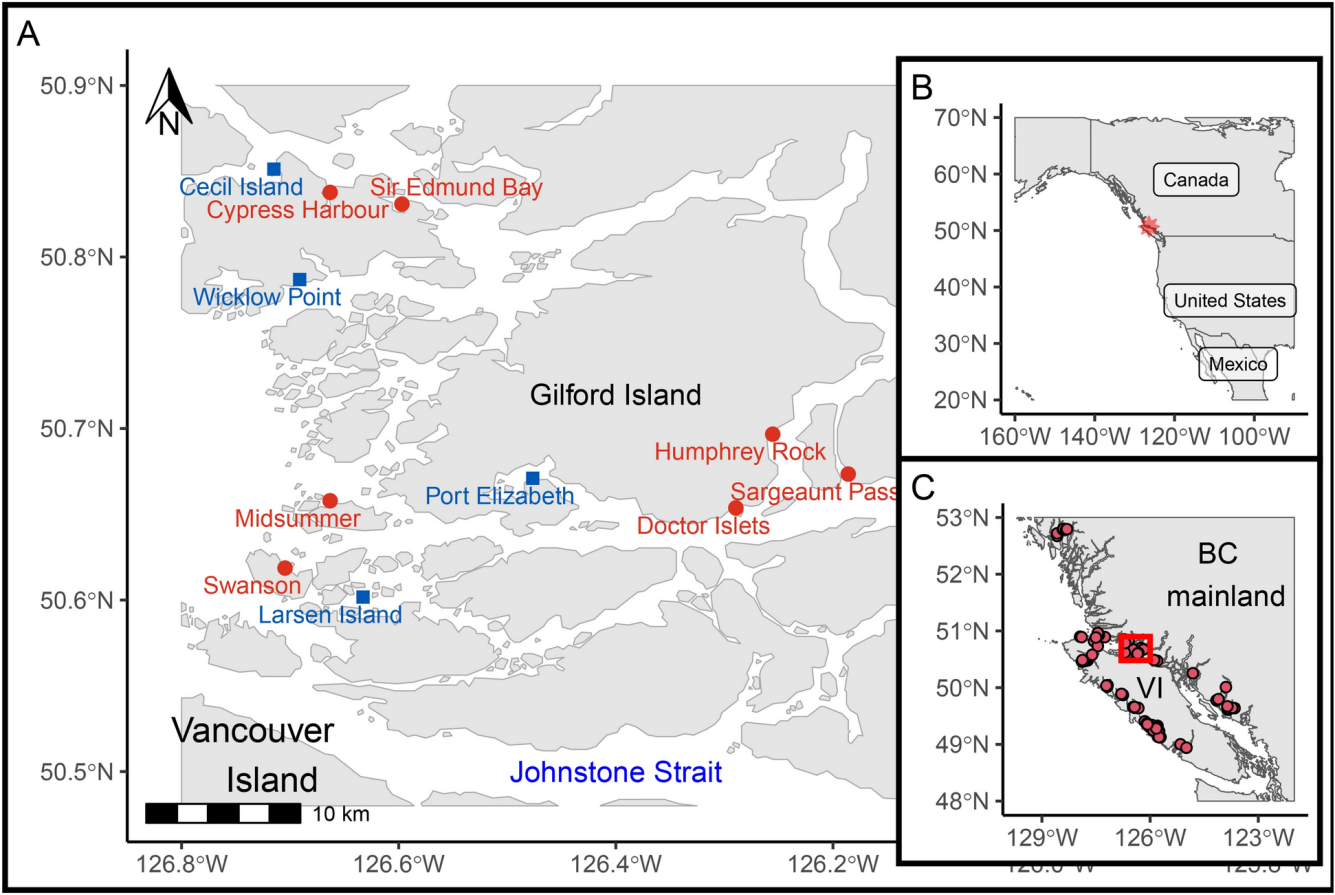
Environmental DNA samples were collected from Atlantic salmon netpen aquaculture sites in the Broughton Archipelago (**A**), located on Canada’s West Coast (**B**), on the north end of Vancouver Island in British Columbia (**C**). In panel A, red circles and text identify active farms and blue squares and text identify decommissioned farms. Panel B shows the Broughton Archipelago (red star) on a map of North America. Panel C shows all salmon aquaculture netpens on the British Columbia coast (at the time of the study) and around Vancouver Island (VI) as well as the Broughton Archipelago (red rectangle). This map was created by the authors in R version 4.3.0^84^ (https://www.r-project.org/) using shoreline data from the Global Self-consistent, Hierarchical, High-resolution Geography Database Version 2.3.7^85^ (https://www.soest.hawaii.edu/pwessel/gshhg/).

The Broughton Archipelago is a complex collection of islands between Vancouver Island and mainland British Columbia (Fig. 7). Salmon aquaculture was first introduced to this region in 1988. In 2018, three First Nations in this region, Kwikwasut’inuxw Haxwa’mis, Mamalilikulla, and ’Namgis, signed a Letter of Understanding with the Province of British Columbia regarding finfish aquaculture in the Broughton Archipelago. This led to an agreement between the Nations and tenure holders (MOWI Canada West and Cermaq Canada) around the transition of aquaculture practices in the region (Broughton Aquaculture Transition Initiative, BATI). The research described herein was conducted under the Indigenous Monitoring and Inspection Plan (IMIP), that was created alongside BATI.

Eleven farms sites were monitored during the course of this study (Fig. 7), with three levels of activity: “active” (fish on site), “fallow” (infrastructure present, no fish on site), and “decommissioned” (infrastructure removed). Sites were visited approximately once a month, weather permitting, from October 2021 to March 2023 (Table S3).

### Environmental DNA collection

Environmental DNA (eDNA) was collected using the EZ-eDNA™ pump and filter system developed by RKS Laboratories Ltd. (Qualicum Beach, BC, Canada), described in^86^. This system consisted of a 12-volt pressure regulated diaphragm pump, coupled with a programmable flow controller set at 5 L. The sample water was pumped through two hollow membrane filter cartridges connected in parallel (field duplicates). Each filter cartridge has two luer locks to attach intake and discharge tubing. The hollow membrane filter consists of 120 Polyethersulfone (PES) membrane tubes with a nominal pore size of 0.1–0.45 $\mu \hbox {m}$ (RKS Laboratories Ltd.). All eDNA samples used a total of 5 L (approximately 2.5 L passing through each filter cartridge). Once the filtration was concluded, an air pump included in the system was used to remove residual water from inside the filter housing. Next, 2 ml of RNA-Later™(ThermoFisher Scientific, Waltham, MA, USA) was injected by syringe into the filter cartridge for preservation, and luer lock end caps were attached for storage. The filter cartridge was then refrigerated for 24–48 h, and subsequently preserved in a -20 °C freezer, until ready to be extracted and analyzed.

At each visit to an active farm, eDNA samples were collected from within netpens and from transects surrounding the farm superstructure. Half of the total active pens on site were sampled at each farm visit (4–6 pens), including one pen (i.e. control pen) that was identified at the beginning of the production cycle and consistently sampled at each visit. Pens with recent mortality were prioritized for sampling. For samples collected from within netpens, the intake hose was submerged to 8 m depth and kept stationary using a weighted pole while 5 L of seawater was pumped (2.5 L per filter cartridge). At active or fallow farms (infrastructure present), transect samples were collected on each of the four sides of the farm superstructure, parallel to the side of the superstructure and at a distance of approximately 20–50 m from the structure’s edge, depending upon the presence of obstacles (boats and anchor lines) and conditions (weather and tides). Five L (2.5 L per cartridge) of seawater was pumped from a depth of approximately 8 m by attaching the intake hose to a downrigger weighted with a 11 kg lead ball. The rate at which each transect was completed by the boat was determined by the length of the side of the superstructure combined with the target of pumping 5 L (pumping took approximately 5–7 min). At decommissioned sites, transects were conducted by boat in four directions to roughly travel the shape of a square with 100 m sides (5 L of seawater collected per direction, to mirror farm samples).

If multiple sites were to be sampled for eDNA in a single day (fish were never sampled from multiple sites in a single day due to biosecurity concerns), a separate pump system was used at each site. When arriving at a sampling location, a field blank (i.e. negative control sample), consisting of 5 L of sterile distilled water was pumped and filtered through two parallel filter cartridges. When sampling at active farms, the transects outside the farm were conducted first, followed by the netpens, with the expectation that eDNA concentrations were likely to be greater inside netpens. When each netpen was sampled, the pump system was first flushed with 5 L of water from that netpen before a sample was taken. Such “flushes” were not conducted between transect samples. After each sampling day, 5 L of 10% bleach were run through pump systems, followed by 5 L of sodium thiosulfate, to neutralize the bleach. The exterior parts of the apparatus were also cleaned with these solutions.

As the composition of salmon feed can include a variety of marine fishes, and possibly nucleic acids from infectious agents present in fish used for feed, we recognized that salmon feed pellets could contribute to eDNA detections around farms, complicating the interpretation of the resulting qPCR data. Hence, we obtained samples of feed used in salmon hatcheries and for grow-out on farms and tested them across the same assays used on fish and eDNA in our study. While this was not an exhaustive effort, as feeds used by commercial farms likely include multiple brands, and can vary with life stage and husbandry concerns, it provided some insight to the interpretation of our results.

### Atlantic salmon sampling

During visits to active farms, Atlantic salmon were collected from netpens for histological analysis and qPCR IA screening. Thirty live, apparently healthy fish as well as up to 10 moribund and/or dead fish were collected from each farm on the day of the visit. Fifteen of the thirty live fish were always collected from the control pen, while the other fifteen were usually collected from a secondary pen, decided by the veterinarian (EDC) on the day of the visit, based on the clinical conditions of the fish and the mortality data for the 30 days before the visit, provided by the facility staff. Collection of moribund/dead fish prioritised these same two pens; however, if very few fish were obtained from these two pens, additional pens were involved in the collection of moribund/dead fish. Moribund fish were either scooped up with a long dip net from inside the pen (possible due to a lack of startle reaction, typical of a lethargic state) or obtained by pumping up dead fish from the bottom of the pens. In one farm where no “mort pumps” were present, professional divers were hired to collect and provide morts from the bottom of the pens.

Small portions of the gills, liver and anterior kidney, to be used for molecular analysis, were sampled (in triplicate) from every fish (live, moribund or dead) and preserved in RNAlater (1.5 mL). Clinical observations were made during dissection. Tissues were stored at 4 °C, and transferred back to BATI’s laboratory facility in Nanaimo, BC. One set of gill, liver, and kidney samples was delivered to DFO’s Pacific Biological Station Molecular Genetics Laboratory within 1–2 days of sampling. Samples were processed for nucleic acid extraction upon arrival.

#### Ethics statement

Fish were collected and euthanized by aquaculture facility personnel (not authors or BATI technicians). Therefore, all work with animals was performed according to the Canadian Council on Animal Care’s (CCAC) Guide to the Care and Use of Experimental Animals, and protocols were approved by Fisheries and Oceans Canada (DFO) through its Pacific Region Animal Care Committee (conditions of license for salmon farm operations). Live-sampled fish were euthanised via overdose of tricaine methanesulfonate (Syndel laboratories Ltd., Nanaimo BC, Canada). All tissue samples involved were collected under the IMIP agreement between the aquaculture companies and BATI. Where applicable, methods herein are reported in accordance with the ARRIVE guidelines.

### Molecular analysis

#### Environmental DNA extraction

DNA and RNA were extracted from hollow membrane filter cartridges using a modified version of the protocol (as described below) from the Invitrogen™ PureLink™ Viral RNA/DNA Mini Kit (Cat# 12280050, Thermo Fisher Scientific). We used two methods, which we label here as “manual extraction” and “semi-automated extraction.” Manual extraction was used for samples collected from September 2021 through April 2022 and semi-automated was used for samples collected thereafter, once the extraction machine became available for use.

For manual extraction, all components, aside from ethanol, used in extraction were included in the Invitrogen Mini Kit. First, RNAlater was removed from the filter cartridges using an electric air pump. Next, 2 ml of lysis buffer mixed with Proteinase K (2 mg/ml) and Carrier RNA (1 µg/sample) was pipetted into each filter cartridge. After thorough vortexing (2400 rpm, 5 min), the samples were incubated at 56 °C with slow shaking for 30 min to ensure complete lysis. The lysate was manually collected from each filter using 5 ml syringes and transferred to separate 5 ml tubes. The 5 ml tubes were then cetrifuged at maximum speed (2400 *g*) for 5 min to remove any remaining unlysed debris or particles. The supernatant was transferred to a new tube where ethanol was added to reach a final concentration of 37% ethanol. The resulting solution was then loaded onto a silica spin column, where a vacuum pulled the solution through. The column was then washed twice with Wash Buffer. Finally, RNA/DNA was eluted in 50 µl of sterile, RNase-free water.

For semi-automated extraction, RKS Laboratories Ltd. (Qualicum Beach, BC, Canada) developed a specialized system based on the manual extraction method. This semi-automated system is capable of processing 12 filters simultaneously. It removed RNAlater from the hollow membrane filters by pumping 5 ml of lysis buffer from Nanjing Rebeads Biotech Co., Ltd (Cat# RBX024-1000, Nanjing, China) into each filter cartridge. Subsequently, 2 ml of lysis buffer (Nanjing Rebeads) mixed with Proteinase K (2 mg/ml, Invitrogen) and Carrier RNA (1 µg/sample, Invitrogen) was introduced into each filter cartridge. The system shook and incubated the 12 filters at 40–50 °C for 30 minutes to ensure thorough lysis. After lysis, the system used air pumps to push the lysate out of the filters into separate 5 ml tubes for each filter. The remaining steps, from centrifuging in 5 ml tubes through elution, were conducted identically to those described above for manual extraction. Preliminary comparison of results from the extraction protocols was performed prior to the switch to semi-automated extraction, but we also discuss the effects on results below.

Feed pellets that were on hand at the experimental fish laboratory at DFO’s Pacific Biological Station and Pacific Science Enterprise Centre were extracted for analysis. The first sample, “feed 1,” was 6 mm BioBrood pellets (Bio-Oregon, Longview, WA, USA), which are intended for fish from 400 to 1000 g. Feed 2 was 1 mm pellets from EWOS (Surrey, BC, Canada). Both feeds were collected from bags that were previously opened, resulting in potential for environmental contamination. Duplicate samples were prepared from each feed type, each sample from feed 1 consisting of 2 pellets and each sample from feed 2 consisting of 6 pellets. The pellets were soaked in the Purelink lysis buffer at 40–50 °C for 30 min with slow shaking, homogenized at 30 Hz for 5 min, and then centrifuged at 3000 rpm for 10 min to allow for separation of liquid and solid layers. Ethanol was added to the supernatant to reach a final ethanol concentration of 37%. The remaining steps followed the same procedure as the eDNA manual extraction.

#### *Salmo salar* tissue extraction

Tissue preparation for qPCR followed Miller et al.^87^ for RNA extraction, but instead of also extracting DNA, DNA was not enzymatically removed from the RNA extraction. We found this procedure reduced dilution of the RNA without sacrificing DNA detections. Single tissues were homogenized on separate plates but equal aliquots for each of: gill, kidney, and liver tissue homogenates were combined for extraction.

#### High-throughput polymerase chain reaction

The BioMark™platform (Fluidigm Corporation, CA, USA; now Standard BioTools), a nanofluidic automated real-time quantitative PCR system, was used to test eDNA samples using 7 salmonid, 9 marine fish, and 32 IA assays (Tables 1, S4). The same platform was used to test *S. salar* mixed tissue samples using the same 32 IA assays run for eDNA. This section briefly describes how samples are prepared for use on the BioMark, but detailed methods are available in previous publications from the DFO Molecular Genetics Lab^16,34,87^.

Following extraction, RNA in the resultant nucleic acid (RNA and DNA) was reverse-transcribed to cDNA. For *S. salar* tissues, nucleic acids were first normalized to 62.5 ng $\mu$L^−1^ using a Biomek NXP™automated liquid-handling instrument, and 1 $\mu$g of normalized nucleic acid was converted to cDNA. The normalisation step for salmon samples ensures that IA results are reported relative to total nucleic acid, the vast majority of which comprises host DNA and RNA. For eDNA samples, because the eDNA itself comprises the nucleic acid in each sample, no such nucleic acid normalization was conducted prior to converting RNA to cDNA. Because reaction volumes used by the BioMark (7 nL) are so much smaller than those used in conventional qPCR ($\sim$25 $\mu$L), a pre-amplification step—as recommended by the manufacturer—was used to increase sensitivity with such small volume reaction wells. In this step, cDNA/DNA underwent 17 PCR cycles using a 1/10 dilution of all primers (no probes) targeting sequences to be assayed on the BioMark^87^. Miller et al.^87^ conducted extensive analyses regarding the impacts of this pre-amplification step, with no negative impacts on specificity of the final assays identified when post-amplification qPCR was carried out using TaqMan probes.

Following pre-amplification, samples and assays were pippetted into the respective loading wells of 96 x 96 well dynamic arrays (Standard BioTools). Serially diluted artificial probe constructs (APCs) and processing controls were included, as per Miller et al.^87^, and a second fluorescent dye was included in all reaction chambers to detect potential laboratory contamination by APCs. The purpose of APCs is to confirm assay function (positive control), calculate assay efficiency, and facilitate estimation of IA DNA/RNA copies. The dynamic arrays were run on the BioMark, individual runs were analyzed for cycle threshold using the Fluidigm Real-Time PCR Analysis software, and scored data were exported to a purpose-built SQL-database. Technicians were blind to the identity of the samples throughout the laboratory work and post-processing.

### Consequences of infection literature search

To contextualize our IA release results, we created “consequence scores” for IAs based upon previously published, peer-reviewed literature (“consequence” in Figs. 1 and 6). For each IA that was detected during the study (Table 1) we searched the existing literature with the goal of developing scores representing the weight of evidence suggesting pathogenicity for a given agent. We considered whether negative impacts of infection have been observed for three categories including challenge studies, histological and/or clinical examinations, and field or epidemiological studies. Evidence for each consequence category was scored from 0 to 2, where 0 represents no evidence of a negative impact and 2 represents robust evidence of negative impacts in at least one species of *Oncorhynchus*. Scores of 2 required evidence of high mortality in challenge or epidemiological studies or severe lesions in histological studies. A score of 1 indicates some evidence of negative impact in *Oncorhynchus* or robust evidence of negative impacts in *S. salar* when evidence is lacking for *Oncorhynchus* spp. The scores for each category were summed so that consequence ranged from 0 to 6 with an associated uncertainty score ranging from 0 to 3 (Table S1). We prioritized studies featuring *Oncorhynchus* spp. but considered studies of *S. salar* where studies were lacking for *Oncorhynchus* spp. If no studies featuring *Oncorhynchus* spp. were found for a given agent for one of the categories, a point was added to the uncertainty score (uncertainty could range from 0 to 3, where 3 represents no studies conducted on *Oncorhynchus* spp. for any consequence categories). This approach gives each category equal opportunity to contribute to the overall consequence score, which is an imperfect simplification of the situation but we have provided the rationale behind all scores (Table S1). Furthermore, consequence scores do not reflect the complexity of contradictory findings amongst studies (e.g., an IA has negative impacts in one *Oncorhynchus* species but not another).

### Comparison of sea louse counts with qPCR results

As a condition of their license to operate, aquaculture facilties in BC must routinely count parasitic copepods (i.e. “sea lice”, *Lepeophtheirus salmonis* and *Caligus clemensi*) in their netpens (https://open.canada.ca/data/en/dataset/3cafbe89-c98b-4b44-88f1-594e8d28838d/resource/f6a948f3-504c-34b0-ac30-58a6b06981e6). A thorough description of the counting process can be found in^88^. The DFO dataset of sea lice counts, collected independently from our eDNA samples, constitutes the only dataset that we could use to ground truth our results from eDNA sampling and laboratory analysis. The DFO Aquaculture Management Division (AMD) provided us with a netpen-level (online version is averaged by farm) dataset of industry sea louse counts from farms in the Broughton Archipelago. Industry sea louse counts could then be matched to eDNA samples for each specific netpen so that we could test for agreement (presence/absence) between the two methods and conduct other statistical analyses.

### Statistical analysis

All IA assays, both for eDNA and *S. salar* tissues, were run in duplicate on the Biomark qPCR dynamic arrays. These analytical duplicates were averaged, and IAs not detected in duplicate were considered non-detections. Field replicates (samples collected simultaneously in parallel on the EZ-eDNA™ pump and filter system) were averaged when both were positive. If only one of two field replicates was positive, the sample was considered positive overall and the positive value was assigned (i.e., the positive value was not averaged with zero). We took this approach because the heterogenous distribution of some IAs (e.g., spore forming IAs) in the water column can lead to false negatives^89^. This approach of not averaging with zero would only have consequences for figures in this study, as models are all based on presence/absence. For IAs, processed results of qPCR data in Ct were converted to copy number calculated from the standard curve established by the APCs. For salmonid and marine fish assays, results were kept in Ct units. Although limit of detection (LOD) estimates have been developed for all IA assays run (Table S4), we did not apply LOD cutoffs to the data prior to analysis. If a field blank was positive for a given assay during a site visit we removed any detections from collections at the same site, date, and assay with a higher Ct value (weaker detection) from the dataset but left other detections unchanged. We did so under the assumption that detections stronger than those in the field blanks could not be caused by contamination.

During exploratory data analysis we determined that the type of lysis buffer used for eDNA filter extraction (Purelink versus Rebead) had an impact on qPCR results, with the Rebead buffer resulting in lower recovery of total nucleic acids, and particularly lower detection of many RNA viruses. We suspect that Rebead was less efficient at recovering RNA because it does not appear to contain Guanidine Thiocyanate, a potent inhibitor of RNase, which would reduce extraction efficiency, particulary for RNA viruses. Therefore, IA taxa more likely to be collected as RNA than DNA would be under-represented from samples collected from May 2022 onward. Other assays appeared less impacted by the lysis buffer used (e.g., salmonid and marine fish assays). When averaging across assays, we determined that Ct values were approximately 2.5 units greater when Rebead buffer was used instead of Purelink. Therefore, to account for this shift in extraction efficiency, the type of lysis buffer used for each sample was included as a fixed effect in all models. We avoided comparisons of prevalences or loads across time for eDNA detections as these would be impacted by the type of lysis buffer used, at least for some assays, given that the switch in lysis buffer was confounded with sampling date. The overall impact of the switch in lysis buffer midway through the project is that IA presence and load are likely to be underestimated, lending a conservative bias to IA detections.

To test whether the probability of detection was associated with the sample site (active farm versus inactive site) we used a generalized linear mixed model (GLMM) with an autoregressive (AR1) correlation structure to account for temporal autocorrelation at each sampling site. This GLMM structure was used both to characterize IA release and marine fish or salmon exposure to release (Fig. 6). The model included fixed effects for sample type (active farm versus inactive site—the variable of interest) and lysis buffer type (Purelink versus Rebead) as well as random intercepts for the sample month and year combination (spanning the 17 month study period) and the sample location (eleven sites). Contrary to the findings of Barrett et al.^9^, who found that inactive sites with infrastructure (“fallow” sites in this study) and those with no infrastructure (“decommissioned” sites in this study) had varying magnitudes of difference from active aquaculture sites, our initial models using these multiple categories of inactive sites did not reveal significant differences in eDNA concentrations between fallow and decommissioned sites; therefore, we grouped fallow and decommissioned sites together as inactive sites. Furthermore, fallow sites were not abundant in this study; most samples labeled as inactive sites were decommissioned farms (Table S3). In addition, we pooled samples collected from within netpens with those collected in transects around farms because no barriers prevent the movement of eDNA in and out of nets and transect samples were collected only a short distance ($\sim$50 m) away from netpen samples. The response variable was eDNA detection (IAs, marine fish, salmon species) and thus the GLMM was a binomial model with a logit link function. Separate models were run for each response variable using the glmmTMB package^90^ in R version 4.3.0^84^. Models that did not converge were removed from analysis and not considered for interpretation. We tested for the uniform distribution of residuals by visualizing a qq-plot and a plot of the residuals against the predicted value using the DHARMa package^91^.

Our experimental design was similar to that used by Shea et al.^18^, who sampled eDNA adjacent to active and fallow aquaculture sites (although not within netpens) in the Broughton Archipelago and Discovery Islands during the summer months of 2016–2018. We used the publicly available data from Shea et al.^18^ (https://datadryad.org/stash/dataset/doi:10.5061/dryad.r7sqv9s98) to test for the repeatability of results for assays (n = 10) that overlapped between the two studies. Again, we used GLMMs fitted using the glmmTMB R package^90^. We did not use an AR1 correlation structure because samples were only collected during a three month period each summer in^18^. These GLMMs included a random intercept for study year, a random intercept for location, and the fixed variable of interest, sample location (active farms versus inactive sites). To determine whether or not the results were similar between the studies, we used simple linear regression.

To estimate a mean effect of farm status on detection probability in our study and in the reanalyzed data from Shea et al.^18^, we employed a meta-analytical approach (e.g., Worm et al.^92^) where each IA’s fitted model (within its respective data set) played the role of a “study” in a typical meta-analysis. We used the R package “meta”^93^ to conduct an inverse-variance weighted random-effects meta-analysis. The Paule-Mandel estimator was used as the method for estimating among-pathogen variance^94,95^.

To compare industry sea lice counts to sea lice loads in eDNA samples we used a similar GLMM with AR1 autocorrelation structure and random intercepts as described earlier. We created a binomial GLMM to test the relationship between industry counts (fixed effect of interest) and the likelihood of a detection in eDNA samples, for both *C. caligus* and *L. salmonis*. We also created a gaussian LMM to test for a positive correlation between industry counts and log-transformed copepod RNA/DNA copies in eDNA samples. As previously, lysis buffer was included as a fixed effect.

## Supplementary Information

Supplementary Information.

## Data Availability

The datasets generated during and/or analysed during the current study are available in the following GitLab repository, https://gitlab.com/mgl-published-repo/broughton-archipelago-edna-study-2024/-/tree/main.
