# Supplementary material for: Infectious agent release and Pacific salmon exposure at Atlantic salmon farms revealed by environmental DNA
Source: Sci Rep. 2024 Dec 28;14:31488. doi: 10.1038/s41598-024-83250-5 (PMC11682043; doi:10.1038/s41598-024-83250-5)
Supplement: Supplementary file 1 — Supplementary Information. [file 41598_2024_83250_MOESM1_ESM.pdf]

Supplementary Information:

Infectious agent release and Pacific salmon exposure at Atlantic salmon farms revealed by environmental DNA

July 29, 2024

Tables

Table S1: This table summarizes the evidence from the literature for the consequence scores assigned to each pathogen assayed in this study. Evidence is categorized as pertaining to challenge studies, histopathological studies, and field studies. Each category is assigned a score from 0 to 2 (bold and in parantheses) depending upon the cumulative weight of evidence for negative consequences. When evidence is absent for a category, one point is added to the "no studies count" (aka uncertainty based on the literature).

taxa **(total score)**	Challenge studies with physiological or survival impact	Gross lesions or Histological evidence of impact	Evidence of impact in wild salmon populations or field studies (including netpens)	Salmon species	No studies count
Candidatus Branchiomonas cysticola **(2)**	No challenge studies **(0)**	-observed in epitheliocysts in *S. salar* but also elsewhere in association with necrosis and inflammation [1] -observed in epitheliocysts in free-ranging *O. tshawytscha* [2] **(1)**	-seasonal neg correlations with body condition for *O. tshawytscha* and *O. kisutch* [3] -many correlations with gill biomarkers in early marine *O. tshawytscha* [2] **(1)**	*S. salar*, *O. tshawytscha*	**1**
Flavobacterium psychrophilum∗ **(5)** ∗(studies for impacts in FW, no evidence of impacts for this bacterium in SW)	-*O. mykiss* and *O. kisutch* particularly susceptible [4] -IP injection lead to high mortality in *O. keta* [5] **(2)**	-Erosion of skin along peduncle or tail, pail or necrotic gills, many other clinical signs [4, 6] **(2)**	-Epizootics reported worldwide in many fish species but almost all described from culture [7] -Higher loads of this bacterium measured in *O. mykiss* below a hydraulically challenging migration feature (spawning migration) compared to above [8] **(1)**	All Pacific salmon species	**0**
Moritella viscosa **(2)**	-*S. salar* bath challenged with this bacterium showed 33% mortality after 10 days [9] -more examples in [10] -No challenge studies with Pacific salmon **(1)**	-immunohistochemistry showed the bacterium associated with skin ulcers [9] **(1)**	-No studies on wild populations -Some netpen outbreaks described in [10] but featuring low mortality **(0)**	*S. salar*	**3**

taxa **(total score)**	Challenge studies with physiological or survival impact	Gross lesions or Histological evidence of impact	Evidence of impact in wild salmon populations or field studies (including netpens)	Salmon species with clinically confirmed infection	No studies count
Piscirickettsia salmonis **(5)**	-high mortality in *O. gorbuscha* and *O. keta* infected by cohabitation [11]<br>-multiple strains cause high mortality in *O. kisutch*[12]<br>-*O. tshawytscha* injected with materials from a BC outbreak experienced 50-88% mortality[13]<br>**(2)**	-Ulcers and exopthalmia observed in mortalities in Chinook in a BC netpen, impacts to liver and kidney observed in histopathology [13]<br>-described in detail in [14]<br>**(2)**	-No studies on wild fish<br>-Cumulative mortality reached 34% among *S. salar* held in experimental netpen [15]<br>-Epizootics reaching 90% for cultured *O. kisutch* in Chile [16]<br>**(1)**	*S. salar*<br>*O. keta*<br>*O. gorbuscha*<br>*O. tshawytscha*	**0**
Renibacterium salmoninarum **(5)**	-High susceptibility in challenged *O. kisutch, O. tshawytscha, O. mykiss* [17, 18]<br>- 85-95% mortality following IP injection in *O. tshawytscha* [19]<br>**(2)**	-Impacts to kidney summarized in [20]<br>**(2)**	-Infections associated with decreased weight for first year marine *O. tshawytscha* [3, 21]<br>-Little information on associated mortality in marine environment<br>**(1)**	*O. tshawytscha*<br>*O. kisutch*<br>*O. keta*<br>*O. mykiss*	**0**
Tenacibaculum dicentrarchi **(5)**	-*O. tshawytscha* experienced 28% morbidity after immersion challenge [22] - *S.salar, O. mykiss, O. kisutch* challenged by bath immersion for one hour - 63, 93, and 0% mortality respectively [23]<br>**(2)**	-BC *O. tshawytscha* displayed ulcerative dermatitis, myositis, liver intrusion, and systemic infection [24]<br>-*S. salar* with oral lesions, necrosis in submucosa, down to bone [25]<br>**(2)**	-causative agent responsible for mortality of wild BC *O. tshawytscha* in holding study [24]<br>**(1)**	*S. salar*<br>*O. tshawytscha*<br>*O. mykiss*	**0**
Tenacibaculum finmarkense **(3)**	-10% mortality for both bath-exposed and cohab *S. salar*, over 12 days [25]<br>-10-80% mortality for bath-exposed *S. salar*, 3-20% for cohabs, over 21 days [26]<br>**(1)**	-mouth lesions with destruction of epidermis and invasion into dermis in *S. salar* [26]<br>**(1)**	- Farms in Chile with *O. mykiss, O. kisutch* experiencing lesions from which *T. finmarkense* was isolated experienced 2-3% mortality. [27]<br>**(1)**	*S. salar*<br>*O. kisutch*<br>*O. mykiss*	**2**

taxa **(total score)**	Challenge studies with physiological or survival impact	Gross lesions or Histological evidence of impact	Evidence of impact in wild salmon populations or field studies (including netpens)	Salmon species with clinically confirmed infection	No studies count
Tenacibaculum maritimum **(6)**	-60–100% mortality for both exposed and cohab *S. salar* over 21 days [25]<br>-60–100% mortality for bath exposed *O. tshawytscha* and 100% showing clinical signs of infection [22]<br>**(2)**	-ulceration and edema formation exposing demis and musculature of caudal trunk; penetration and necrosis of gill filaments; extensive replacement of oral mucosa by bacterial growth in *s. salar* [25]<br>-*T. maritimum* isolated from lesions on farmed *O. mykiss* in Chile [28]<br>-*T. maritimum* isolated from gill lesions on farmed *O. tshawytscha* in California [29]<br>**(2)**	-Associated with decreased wild survival in *O. tshawytscha* and decreased body condition in *O. kisutch, O. tshawytscha* along the BC coast [3]<br>-PCR-detected in wild Fraser River *O. nerka* with modeled theoretical impacts for survival (5-56% decrease through Discovery Islands region migration) [30]<br>-mortality events in cultured *O. tshawytscha* in New Zealand caused by /T. maritimum] [22]<br>**(2)**	*S. salar*<br>*O. mykiss*<br>*O. tshawytscha*	**0**
Atlantic salmon calicivirus **(0)**	-challenge trials in *S. salar* show that calicivirus replicates and establishes a systemic infection [31]<br>**(0)**	-Found in fish with HSMI but no strong evidence of pathogenicity<br>**(0)**	-No studies on wild populations<br>**(0)**	*S. salar*	**3**
Cutthroat Trout virus 2 **(2)**	-challenge of *O. nerka, O. gorbuscha, O. tshawytscha* resulted in persistent infections without disease and mortality but minor pathology [32]<br>**(1)**	-CTV-2 linked to lesions in the brain of *S. salar* with mild neural necrosis [33]<br>-mild endocarditis in *O. tshawytscha* and intratubular protein casts in *O. tshawytscha* and *O. nerka* [32]<br>**(1)**	-No studies on wild populations<br>-Longitudinal study on salmon farms showed elevated levels of CTV2 in dead *S. salar* relative to live [34]<br>**(0)**	*S. salar*<br>*O. tshawytscha*<br>*O. nerka*	**1**
Erythrocytic necrosis virus **(2)**	-*O. keta* experimentally infected showed disequilibrium for a number of blood parameters (low glucose, RBC, etc) and performed poorly after a stress test but no mortalities mentioned [35]<br>-*O. kisutch, O. tshawytscha, O. nerka* refractory to experimental infection [36]<br>**(1)**	-Following experimental infection with ENV, *O. keta* showed intracyto-plasmic erythrocytic inclusions containing ENV [37]<br>**(1)**	-No studies of impacts to wild salmon populations<br>**(0)**	*O. keta*<br>*O. gorbuscha*	**1**

taxa **(total score)**	Challenge studies with physiological or survival impact	Gross lesions or Histological evidence of impact	Evidence of impact in wild salmon populations or field studies (including netpens)	Salmon species with clinically confirmed infection	No studies count
Putative narnavirus **(0)**	-No studies **(0)**	-No studies **(0)**	-No studies **(0)**	*S. salar* and *O. tshawytscha* by molecular detection only [33]	**3**
Piscine orthoreovirus-1a **(3)**	-a challenge study showed a cause and effect relationship between a BC isolate of PRV-1a and mild to moderate inflammatory heart lesions in *S. salar* [38]<br>-several challenge studies have found minor physiological impacts of experimentally applied PRV infection in Pacific salmon and no associated mortality [39, 40]<br>**(1)**	-epidemiological observations of heart lesions associated with PRV-1a in farmed *S. salar* in BC [41]<br>-epidemiological studies associate PRV in farmed and wild *O. tshawytscha* with the disease jaundice/anemia, a consequence of rupture of red blood cells infected by the virus [42, 2]<br>**(1)**	-PRV had negative associations with population-level survival for *O. tshawytscha* [3]<br>-negative associations between PRV1a load and body condition for both Chinook and Coho [3, 43]<br>**(1)**	*S. salar*<br>*O. tshawytscha*<br>*O. nerka*	**0**
Caligus clemensi **(2)**	-no challenge studies **(0)**	-skin lesions in *O. gorbuscha* were associated with the presence of non-motile *C. clemensi* [44]<br>**(1)**	-*C. clemensi* infestation is correlated with reduced growth, foraging success, and competitive ability in wild juvenile *O. nerka* [45, 46, 47]<br>**(1)**	*O. gorbuscha*<br>*O. nerka*	**1**
Facilispora margolisi **(0)**	-No challenge studies **(0)**	-No histo in salmon **(0)**	-No evidence of impact on wild populations **(0)**		**2**
Ichthyophonus spp. **(2)**	-*O. tshawytscha* juveniles IP-injected or exposed to *I. hoferi* infected herring suffered some mortality, but largely during coinfection with *Flavobacterium* spp. [48]<br>-experimentally infected *O. mykiss* had significantly diminished swimming ability relative to controls [49]<br>**(1)**	-In *O. tshawytscha*, histological evidence of *I. hoferi* invasion and spore growth systemically, represented in heart, liver, skeletal muscle, spleen, kidney, and mesentery. Some inflammatory reaction [48]<br>**(1)**	-Studies on migrating adult *O. tshawytscha* in the Yukon river explored the possibility that *I. hoferi* may have population-level impacts but there is little epidemiological evidence for a causal relationship [50]<br>**(0)**	*O. tshawytscha*<br>*O. mykiss*	**0**

taxa **(total score)**	Challenge studies with physiological or survival impact	Gross lesions or Histological evidence of impact	Evidence of impact in wild salmon populations or field studies (including netpens)	Salmon species with clinically confirmed infection	No studies count
Ichthyophthirius multifiliis∗ **(5)** ∗(studies for impacts in FW, no evidence of impacts for this bacterium in SW)	-Many challenge trials for *O. mykiss* to facilitate some test of immune response (*I. multifiliis* is a model organism for immune challenges) [51] **(1)**	-Parasite invades epithelial cells (including gill epithelial) and causes hyperplasia, inflammation and degradation [51] **(2)**	-epizootic outbreaks in prespawn and spawning BC *O. nerka*, estimate to have result in the loss of 153.6 million sockeye fry in 1994 and 1995 [52] **(2)**	*O. nerka* *O. mykiss*	**0**
Ichthyobodo spp. **(4)**	-*O. keta* were experimentally infected with *I.necator* and up to 70% mortality was observed in seawater (but much less in freshwater) [53] -hatchery *O. keta* in Japan were exposed to *I. salmonis* and experienced high infection and 80% cumulative mortality whereas mortality was 0% in a control group [54] **(2)**	-Skin erosion led to osmoregulatory imbalance in *O. keta* [53] -additional descriptions of pathology in [55] **(1)**	-several accounts of largescale mortality in netpen *S. salar* in [55] **(1)**	*S. salar* *O. keta* *O. tshawytscha*	**1**
Kudoa thyrsites **(1)**	-Unknown alternate host of parasite means controlled challenge studies not possible but mortality in *S. salar* has not been observed as a result of infection **(0)**	-In *S. salar*, histological evidence of presence (40-80%), intrusion and reproduction in skeletal and heart muscle resulting in chronic extensive inflammation following natural exposure [56] **(1)**	-No observed negative impacts on wild populations -No survival or physiological impacts in netpens aside from flesh quality impacts **(0)**	*S. salar*	**2**

taxa **(total score)**	Challenge studies with physiological or survival impact	Gross lesions or Histological evidence of impact	Evidence of impact in wild salmon populations or field studies (including netpens)	Salmon species with clinically confirmed infection	No studies count
Loma spp. **(6)**	-*O. tshawytscha* challenged by feeding *L. salmonae* infected gill tissue with almost all fish infected, cumulative mortality was 40-60% across northern and southern populations as well as their hybrid cross, 43 days since challenge [57] **(2)**	-*O. tshawytscha* histological examination revealed systemic infection with *L. salmonae* cyst formation in gills, arteries, liver, muscle, kidney, and heart resulting in inflammation, necrosis, arterial occlusion, pericarditis and tissue degeneration in the tail and head [58] -*O. kisutch* histological analysis of gill and heart revealed *L. salmonae* infection, inflammation, chronic vasculitis and perivasculitis associated with spore dispersion [59] **(2)**	-*O. tshawytscha* *L. salmonae* epizootic event resulted in 10% mortality in a population of 337,869 juvenile chinook in a hatchery setting [58] -*O. kisutch* stocked into seawater pens showed peak summer *L. salmonae* prevalences of 33-65% infection, contributing to high levels of observed mortality (60%) [59] **(2)**	*O. tshawytscha* *O. kisutch*	**0**
Parvicapsula kabati **(1)**	-No challenge studies **(0)**	-Observed in *O. gorbuscha* renal tubules in association with interstitial inflammation [44] **(1)**	-No evidence **(0)**	*O. gorbuscha*	**1**
Paranucleospora theridion **(2)**	-No challenge studies **(0)**	-In a longitudinal farm study of *S. salar*, this parasite was found (via ISH) associated with lesions in multiple tissues(systemic infection) [60] **(1)**	-Multiple examples of a negative association between P. theridion infection intensity and body weight in *S. salar* [60, 61, 62] -Associated mortalities in a case of netpen *S. salar* were 5-12% [60] **(1)**	*S. salar*	**2**

References for Table S1

References

[1] Gjessing, M. C. *et al.* Multi-agent in situ hybridization confirms ca. branchiomonas cysticola as a major contributor in complex gill disease in atlantic salmon. *Fish and Shellfish Immunology Reports* **2**, 100026 (2021).

[2] Wang, Y. *et al.* Infectious agents and their physiological correlates in early marine Chinook salmon (*Oncorhynchus tshawytscha). Conservation Physiology* **11** (2023).

[3] Bass, A. L. *et al.* Identification of infectious agents in early marine chinook and coho salmon associated with cohort survival. *Facets* **7**, 742–773 (2022).

[4] Barnes, E. & Brown, M. L. A review of *Flavobacterium psychrophilum* biology, clinical signs, and bacterial cold water disease prevention and treatment. *The Open Fish Science Journal* **4**, 40–48 (2011).

[5] Misaka, N., Hatakeyama, M., Koide, N. & Suzuki, K. The variation in virulence among flavobacterium psychrophilum strains isolated from chum salmon oncorhynchus keta. **48**, 17–20 (2013).

[6] Starliper, C. E. Bacterial coldwater disease of fishes caused by *Flavobacterium psychrophilum*. *Journal of Advanced Research* **2**, 97–108 (2011).

[7] Nematollahi, A., Decostere, A., Pasmans, F. & Haesebrouck, F. Flavobacterium psychrophilum infections in salmonid fish. *Journal of fish diseases* **26**, 563–574 (2003).

[8] Twardek, W. *et al.* Evidence of a hydraulically challenging reach serving as a barrier for the upstream migration of infection-burdened adult steelhead. *Conservation Physiology* **7**, coz023 (2019).

[9] Løvoll, M. *et al.* Atlantic salmon bath challenged with moritella viscosa–pathogen invasion and host response. *Fish & shellfish immunology* **26**, 877–884 (2009).

[10] Wade, J. & Weber, L. *Characterization of Moritella viscosa and winter ulcer to inform pathogen transfer risk assessments in British Columbia* (Canadian Science Advisory Secretariat, 2020).

[11] Long, A. & Jones, S. R. Piscirickettsia salmonis shedding and tissue burden, and hematological responses during cohabitation infections in chum oncorhynchus keta, pink o. gorbuscha and atlantic salmon salmo salar. *Plos one* **16**, e0248098 (2021).

[12] House, M., Bartholomew, J., Winton, J. & Fryer, J. Relative virulence of three isolates of piscirickettsia salmonis for coho salmon oncorhynchus kisutch. *Diseases of aquatic organisms* **35**, 107–113 (1999).

[13] Brocklebank, J. R., Evelyn, T. P., Speare, D. J. & Armstrong, R. D. Rickettsial septicemia in farmed atlantic and chinook salmon in british columbia: clinical presentation and experimental transmission. *The Canadian Veterinary Journal* **34**, 745 (1993).

[14] Smith, P. A. & Mardones, F. O. Piscirickettsiosis (piscirickettsia salmonis). In *Climate change and infectious fish diseases*, 280–290 (CABI Wallingford UK, 2020).

[15] Jones, S. R., Long, A., MacWilliams, C., Polinski, M. & Garver, K. Factors associated with severity of naturally occurring piscirickettsiosis in netpen-and tank-reared juvenile atlantic salmon at a research aquarium in western canada. *Journal of fish diseases* **43**, 49–55 (2020).

[16] Lannan, C. & Fryer, J. Piscirickettsia salmonis, a major pathogen of salmonid fish in Chile. *Fisheries Research* **17**, 115–121 (1993).

[17] Starliper, C. E., Smith, D. R. & Shatzer, T. Virulence of renibacterium salmoninarum to salmonids. *Journal of aquatic animal health* **9**, 1–7 (1997).

[18] Sakai, M., Atsuta, S. & Kobayashi, M. Susceptibility of five salmonid fishes to renibacterium salmoninarum. *Fish Pathology* **26**, 159–160 (1991).

[19] Beacham, T. & Evelyn, T. Population and genetic variation in resistance of chinook salmon to vibriosis, furunculosis, and bacterial kidney disease. *Journal of Aquatic Animal Health* **4**, 153–167 (1992).

[20] Elliot, D. *Fish viruses and bacteria: pathobiology and protection*, 286–297 (CABI, 2017).

[21] Sandell, T., Teel, D. J., Fisher, J., Beckman, B. & Jacobson, K. Infections by *Renibacterium salmoninarum* and *Nanophyetus salmincola* (Chapin) are associated with reduced growth of juvenile Chinook salmon, *Oncorhynchus tshawytscha* (Walbaum), in the Northeast Pacific Ocean. *Journal of fish diseases* **38**, 365–378 (2015).

[22] Kumanan, K. *et al.* Experimental challenge of Chinook salmon with Tenacibaculum maritimum and Tenacibaculum dicentrarchi fulfils Koch's postulates. *bioRxiv* 2024–03 (2024).

[23] Avendaño-Herrera, R. *et al.* Isolation, characterization and virulence potential of tenacibaculum dicentrarchi in salmonid cultures in chile. *Transboundary and emerging diseases* **63**, 121–126 (2016).

[24] DiCicco, E. *et al.* Tenacibaculosis in wild-caught, captive chinook salmon (oncorhynchus tshawytscha) in british columbia, canada. *bioRxiv* 2023–02 (2023).

[25] Nowlan, J. P., Britney, S. R., Lumsden, J. S. & Russell, S. Experimental induction of tenacibaculosis in atlantic salmon (salmo salar l.) using tenacibaculum maritimum, t. dicentrarchi, and t. finnmarkense. *Pathogens* **10**, 1439 (2021).

[26] Småge, S. B. *et al.* Induction of tenacibaculosis in atlantic salmon smolts using tenacibaculum finnmarkense and the evaluation of a whole cell inactivated vaccine. *Aquaculture* **495**, 858–864 (2018).

[27] Avendaño-Herrera, R., Collarte, C., Saldarriaga-Córdoba, M. & Irgang, R. New salmonid hosts for tenacibaculum species: Expansion of tenacibaculosis in chilean aquaculture. *Journal of fish diseases* **43**, 1077–1085 (2020).

[28] Valdes, S. *et al.* First report and characterization of tenacibaculum maritimum isolates recovered from rainbow trout (oncorhynchus mykiss) farmed in chile. *Journal of Fish Diseases* **44**, 1481–1490 (2021).

[29] Chen, M., Henry-Ford, D. & Groff, J. Isolation and characterization of flexibacter maritimus from marine fishes of california. *Journal of Aquatic Animal Health* **7**, 318–326 (1995).

[30] Bateman, A. W. *et al.* Atlantic salmon farms are a likely source of *Tenacibaculum maritimum* infection in migratory Fraser River sockeye salmon. *Canadian Journal of Fisheries and Aquatic Sciences* **79**, 1225–1240 (2022).

[31] Mikalsen, A. B. *et al.* Characterization of a novel calicivirus causing systemic infection in atlantic salmon (salmo salar l.): proposal for a new genus of caliciviridae. *PloS one* **9**, e107132 (2014).

[32] Long, A. *et al.* Distribution and pathogenicity of two cutthroat trout virus (ctv) genotypes in canada. *Viruses* **13**, 1730 (2021).

[33] Mordecai, G. J. *et al.* Discovery and surveillance of viruses from salmon in British Columbia using viral immune-response biomarkers, metatranscriptomics, and high-throughput RT-PCR. *Virus evolution* **7**, veaa069 (2021).

[34] Bateman, A. W. *et al.* Descriptive multi-agent epidemiology via molecular screening on Atlantic salmon farms in the northeast Pacific Ocean. *Scientific Reports* **11**, 1–15 (2021).

[35] Haney, D., Hursh, D., Mix, M. & Winton, J. Physiological and hematological changes in chum salmon artificially infected with erythrocytic necrosis virus. *Journal of Aquatic Animal Health* **4**, 48–57 (1992).

[36] Evelyn, T. & Traxler, G. Viral erythrocytic necrosis: natural occurrence in Pacific salmon and experimental transmission. *Journal of the Fisheries Board of Canada* **35**, 903–907 (1978).

[37] Eaton, W. Artificial transmission of erythrocytic necrosis virus (env) from pacific herring in alaska to chum, sockeye, and pink salmon. *Journal of applied ichthyology* **6**, 136–141 (1990).

[38] Wessel, Ø. *et al.* Piscine orthoreovirus-1 isolates differ in their ability to induce heart and skeletal muscle inflammation in atlantic salmon (salmo salar). *Pathogens* **9**, 1050 (2020).

[39] Garver, K. A. *et al.* Piscine reovirus, but not Jaundice Syndrome, was transmissible to Chinook salmon, *Oncorhynchus tshawytscha* (walbaum), sockeye salmon, *Oncorhynchus nerka* (walbaum), and Atlantic salmon, *Salmo salar* l. *Journal of Fish Diseases* **39**, 117–128 (2016).

[40] Purcell, M. K. *et al.* Consequences of piscine orthoreovirus genotype 1 (prv-1) infections in chinook salmon (oncorhynchus tshawytscha), coho salmon (o. kisutch) and rainbow trout (o. mykiss). *Journal of fish diseases* **43**, 719–728 (2020).

[41] Di Cicco, E. *et al.* Heart and skeletal muscle inflammation (HSMI) disease diagnosed on a British Columbia salmon farm through a longitudinal farm study. *PLoS One* **12**, e0171471 (2017).

[42] Di Cicco, E. *et al.* The same strain of piscine orthoreovirus (prv-1) is involved in the development of different, but related, diseases in atlantic and pacific salmon in british columbia. *Facets* **3**, 599–641 (2018).

[43] Turcotte, L. D., Bradshaw, J. C., Polinski, M. P. & Johnson, S. C. Piscine orthoreovirus genotype-1 (prv-1) in wild pacific salmon of british columbia, canada: 2011–2020. *Fishes* **8**, 252 (2023).

[44] Saksida, S. *et al.* Parasites and hepatic lesions among pink salmon, oncorhynchus gorbuscha (walbaum), during early seawater residence. *Journal of Fish Diseases* **35**, 137–151 (2012).

[45] Godwin, S. C., Dill, L. M., Reynolds, J. D. & Krkošek, M. Sea lice, sockeye salmon, and foraging competition: lousy fish are lousy competitors. *Canadian Journal of Fisheries and Aquatic Sciences* **72**, 1113–1120 (2015).

[46] Godwin, S., Dill, L., Krkošek, M., Price, M. & Reynolds, J. Reduced growth in wild juvenile sockeye salmon oncorhynchus nerka infected with sea lice. *Journal of Fish Biology* **91**, 41–57 (2017).

[47] Godwin, S. C., Krkošek, M., Reynolds, J. D., Rogers, L. A. & Dill, L. M. Heavy sea louse infection is associated with decreased stomach fullness in wild juvenile sockeye salmon. *Canadian Journal of Fisheries and Aquatic Sciences* **75**, 1587–1595 (2018).

[48] Jones, S. & Dawe, S. *Ichthyophonus hoferi* (Plehn & Mulsow) in British Columbia stocks of Pacific herring, *Clupea pallasi* (Valenciennes), and its infectivity to chinook salmon, *Oncorhynchus tshawytscha* (Walbaum). *Journal of Fish Diseases* **25**, 415–421 (2002).

[49] Kocan, R., LaPatra, S., Gregg, J., Winton, J. & Hershberger, P. Ichthyophonus-induced cardiac damage: a mechanism for reduced swimming stamina in salmonids. *Journal of Fish Diseases* **29**, 521–527 (2006).

[50] Zuray, S., Kocan, R. & Hershberger, P. Synchronous cycling of ichthyophoniasis with chinook salmon density revealed during the annual yukon river spawning migration. *Transactions of the American Fisheries Society* **141**, 615–623 (2012).

[51] Dickerson, H. W. Ichthyophthirius multifiliis, chap. 4, 55–72 (CABI, Cambridge, MA, USA, 2012).

[52] Traxler, G., Richard, J. & McDonald, T. *Ichthyophthirius multifiliis* (ich) epizootics in spawning sockeye salmon in British Columbia, Canada. *Journal of Aquatic Animal Health* **10**, 143–151 (1998).

[53] Urawa, S. Effects of ichthyobodo necator infections on seawater survival of juvenile chum salmon (oncorhynchus keta). *Aquaculture* **110**, 101–110 (1993).

[54] Mizuno, S. *et al.* Epizootiology of the ectoparasitic protozoans ichthyobodo salmonis and trichodina truttae on wild chum salmon oncorhynchus keta. *Diseases of Aquatic Organisms* **126**, 99–109 (2017).

[55] Urawa, S., Ueki, N. & Karlsbakk, E. A review of ichthyobodo infection in marine fishes. *Fish Pathology* **33**, 311–320 (1998).

[56] Moran, J. D. W., Whitaker, D. J. & Kent, M. L. A review of the myxosporean genus *Kudoa* Meglitsch, 1947, and its impact on the international aquaculture industry and commercial fisheries. *Aquaculture* **172**, 163–196 (1999).

[57] Shaw, R. W., Kent, M. L. & Adamson, M. Innate susceptibility differences in chinook salmon oncorhynchus tshawytscha to loma salmonae (microsporidia). *Diseases of aquatic organisms* **43**, 49–53 (2000).

[58] Hauck, A. A mortality and associated tissue reactions of chinook salmon, *Oncorhynchus tshawytscha* (Walbaum), caused by the microsporidan Loma sp. *Journal of Fish Diseases* **7**, 217–229 (1984).

[59] Kent, M. L., Elliott, D. G., Groff, J. M. & Hedrick, R. P. Loma salmonae (protozoa: Microspora) infections in seawater reared coho salmon oncorhynchus kisutch. *Aquaculture* **80**, 211–222 (1989).

[60] Weli, S. C. *et al.* A case study of desmozoon lepeophtherii infection in farmed atlantic salmon associated with gill disease, peritonitis, intestinal infection, stunted growth, and increased mortality. *Parasites & vectors* **10**, 1–13 (2017).

[61] Gjerde, B. *et al.* Estimates of genetic correlations between susceptibility of atlantic salmon to amoebic gill disease in a bath challenge test and a field test. *Aquaculture* **511**, 734265 (2019).

[62] Gunnarsson, G. *et al.* Temporal changes in infections with some pathogens associated with gill disease in farmed Atlantic salmon (*Salmo salar*). *Aquaculture* **468**, 126–134 (2017).

Table S2: Fisheries and Oceans Canada Aquaculture Management Department (AMD) sea lice counts compared to eDNA detections from this study, with data matched by netpen and date. A minus sign indicates absence and a positive sign indicates presence (e.g., AMD+/eDNA- indicates that sea lice were present but not detected in eDNA).

Copepod species	lysis buffer	AMD-/eDNA-	AMD-/eDNA+	AMD+/eDNA-	AMD+/eDNA+	% agreement
L. salmonis	Invitrogen	3	0	51	9	19%
L. salmonis	ReBead	5	0	93	0	5%
C. clemensi	Invitrogen	9	14	11	29	60%
C. clemensi	ReBead	49	2	35	12	62%

Table S3: Type and number of samples collected at seven active farms and four decommissioned farms (Cecil Island, Larsen Island, Wicklow Point, Port Elizabeth) throughout the study period. Duplicate field samples are not counted here.

month	sample	Cecil Island	Cypress Harbour	Doctor Islets	Humphrey Rock	Larsen Island	Sargeaunt Passage	Sir Edmund Bay	Swanson Island	Wicklow Point	Midsummer Island	Port Elizabeth
Oct-2021	S.salar tissues		7	35	39		33		37		23	
Oct-2021	netpen eDNA		4	3	5		6		4		5	
Oct-2021	farm transect eDNA		3	4	4		4		4		4	
Oct-2021	inactive site eDNA	4										4
Nov-2021	S.salar tissues						37	74			19	
Nov-2021	netpen eDNA						6	10			3	
Nov-2021	farm transect eDNA						4	8			4	
Nov-2021	inactive site eDNA	4		4		3				8		4
Dec-2021	S.salar tissues		9		38				35			
Dec-2021	netpen eDNA		4		5				4			
Dec-2021	farm transect eDNA		4		3				4			
Dec-2021	inactive site eDNA					4						
Jan-2022	S.salar tissues		7		38		34	33	38		32	
Jan-2022	netpen eDNA		2		5			4	5			
Jan-2022	farm transect eDNA		4		4		4	4	4		4	
Jan-2022	inactive site eDNA			4		4				4		4
Feb-2022	S.salar tissues		5		39		35	37	40		73	
Feb-2022	netpen eDNA		5		5		6	5	5		10	
Feb-2022	farm transect eDNA		4		4		4	4	4		8	
Feb-2022	inactive site eDNA	8		4								4
Mar-2022	S.salar tissues		7				39	32			35	
Mar-2022	netpen eDNA		6				6	5			5	
Mar-2022	farm transect eDNA		4				4	4			4	
Mar-2022	inactive site eDNA	3								4		4
Apr-2022	S.salar tissues		1		40		37	37	74			
Apr-2022	netpen eDNA		5		5		5	5	10			
Apr-2022	farm transect eDNA		4		4		4	4	8			
Apr-2022	inactive site eDNA	4		4		8				4		
May-2022	S.salar tissues		5	74	39		40	40			38	
May-2022	netpen eDNA		5	9	5		6	5			6	
May-2022	farm transect eDNA		4	8	4		4	4			3	

May-2022	inactive site eDNA	4								4		3
Jun-2022	S.salar tissues				40		39	38	39		40	
Jun-2022	netpen eDNA		6		5		6	5	6		4	
Jun-2022	farm transect eDNA		4		4		4	4	4		4	
Jun-2022	inactive site eDNA	4								4		4
Jul-2022	S.salar tissues		8	34	40		39	37	38		40	
Jul-2022	netpen eDNA		5	4	5		6	4	5		5	
Jul-2022	farm transect eDNA		4	4	4		4	4	4		3	
Jul-2022	inactive site eDNA	4				4				4		4
Aug-2022	S.salar tissues		5	37	40		77	37	40		39	
Aug-2022	netpen eDNA		3	4	5		10	5	6		5	
Aug-2022	farm transect eDNA		3	4	4		7	4	4		4	
Aug-2022	inactive site eDNA	4				8				4		4
Sep-2022	S.salar tissues		4	34	40		40	30	40		40	
Sep-2022	netpen eDNA		4	3	5		6	5	6		5	
Sep-2022	farm transect eDNA		4	4	4		4	3	4		4	
Sep-2022	inactive site eDNA	4				4				4	4	8
Oct-2022	S.salar tissues		6		10			40				
Oct-2022	netpen eDNA		3		5			5				
Oct-2022	farm transect eDNA		4		4			3				
Oct-2022	inactive site eDNA	4								4		
Nov-2022	S.salar tissues			40	23				40			
Nov-2022	netpen eDNA			4					6			
Nov-2022	farm transect eDNA			4	4				4			
Nov-2022	inactive site eDNA					4						4
Dec-2022	S.salar tissues		5	35				6				
Dec-2022	farm transect eDNA		4	4				4	4			
Dec-2022	inactive site eDNA	4			4	4	4			4	3	
Jan-2023	S.salar tissues		6	38								
Jan-2023	farm transect eDNA		4	4								
Jan-2023	inactive site eDNA	4			4	4	4	4		4	4	
Feb-2023	S.salar tissues			39								
Feb-2023	farm transect eDNA		3	3								
Feb-2023	inactive site eDNA	4			2		2	4	4		4	

Table S4: Sequences for all PCR assay primers and probes used to test Broughton Archipelago samples, 2021-2023. LOD refers to Limit of Detection at 95% sensitivity.

Species Name	Assay abbreviation	Primer sequences: Forward<br>Reverse<br>Probe	LOD (Ct/copy)	Origin
Aeromonas salmonicida	ae_sal	TAAAGCACTGTCTGTTACC<br>GCTACTTCACCCTGATTGG<br>ACATCAGCAGGCTTCAGAGTCACTG	22.3/31.4	modified from [1]
Atlantic salmon calicivirus	ascv	ACCGACTGCCCGGTTGT<br>CTCCGATTGCCTGTGATAATACC<br>CTTAGGGTTAAAGCAGTCG	26.4/0.4	[2]
Candidatus Branchiomonas cysticola	c_b_cys	AATACATCGGAACGTGTCTAGTG<br>GCCATCAGCCGCTCATGTG<br>CTCGGTCCCAGGCTTTCCTCTCCCA	26.0/3.9	[3]
Caligus clemensi	ca_cl	GCATTCCCCCGTCTTAATAACA<br>ACCCAGTACCTGCTCCCCTTT<br>TTTACTCCTGTTAAGGGCTT	25.6/1.7	developed by Shaorong Li (MGL)
Cutthroat trout virus-2	ctv-2	CCACTTGTCGCTACGATGAAAC<br>CGCCTCCTTTGCCTTTCTC<br>ATGCCGGGCCATC	27.9/0.4	[2]
Erythrocytic necrosis virus	env	CGTAGGGCCCCAATAGTTTCT<br>GGAGGAAATGCAGACAAGATTTG<br>TCTTGCCGTTATTTCCAGCACCCG	23.1/1.9	[4]
Facilispora margolisi	fa_mar	AGGAAGGAGCACGCAAGAAC<br>CGCGTGCAGCCCAGTAC<br>TCAGTGATGCCCTCAGA	29.3/1.9	[5]
Flavobacterium psychrophilum	fl_psy	GATCCTTATTCTCACAGTACCGTCAA<br>TGTAAACTGCTTTTGCACAGGAA<br>AAACACTCGGTCGTGACC	27.8/7.7	[6]
Ichthyophonus hoferi	ic_hof	GTCTGTACTGGTACGGCAGTTTC<br>TCCCGAACTCAGTAGACACTCAA<br>TAAGAGCACCCACTGCCTTCGAGAAGA	25.4/3.9	[7]
Ichthyophthirius multifiliis	ic_mul	AAATGGGCATACGTTTGCAAA<br>AACCTGCCTGAAACACTCTAATTTTT<br>ACTCGGCCTTCACTGGTTCGACTTGG	23.7/3.9	[5]

Ichthyobodo spp.	Ic_spp	ACGAACTTATGCGAAGGCA	27.3/1.3	[8]
		TGAGTATTCACTYCCGATCCAT		
		TCCACGACTGCAAACGATGACG		
Kudoa thyrsites	ku_thy	TGGCGGCCAAATCTAGGTT	22.1/3.9	[9]
		GACCGCACACAAGAAGTTAATCC		
		TATCGCGAGAGCCGC		
Lepeophtheirus salmonis	le_sa	GACATAGCTTTCCCCCGCTTA	26.7/3.3	[10]
		AGTTCCTGCACCACTTTCTACTAATG		
		ACCCTCTTTGAGTTTATTACT		
Loma salmonae	lo_sal	GGAGTCGCAGCGAAGATAGC	24.7/1.0	[5]
		CTTTTCCTCCCTTTACTCATATGCTT		
		TGCCTGAAATCACGAGAGTGAGACTACCC		
Moritella viscosa	mo_vis	CGTTGCGAATGCAGAGGT	25.8/6.1	[11]
		AGGCATTGCTTGCTGGTTA		
		TGCAGGCAAGCCAACTTCGACA		
Neoparamoeba perurans	ne_per	GTTCTTTCGGGAGCTGGGAG	21.8/3.9	[12]
		GAACTATCGCCGGCACAAAAG		
		CAATGCCATTCTTTTCGGA		
Parvicapsula kabatai	pa_kab	CGACCATCTGCACGGTACTG	25.5/7.7	[5]
		ACACCACAACTCTGCCTTCCA		
		CTTCGGGTAGGTCCGG		
Parvicapsula pseudobranchicola	pa_pse	CAGCTCCAGTAGTGTATTTCA	23.6/7.7	[13]
		TTGAGCACTCTGCTTTATTCAA		
		CGTATTGCTGTCTTTGACATGCAGT		
Paranucleospora theridion	pa_ther	CGGACAGGGAGCATGGTATAG	28.1/1.9	[14]
(syn. *Desmozoon lepeophtheirii*)		GGTCCAGGTTGGGTCTTGAG		
		TTGGCGAAGAATGAAA		
Piscirickettsia salmonis	pisck_sal	TCTGGGAAGTGTGGCGATAGA	23.7/7.7	[15]
		TCCCGACCTACTCTTGTTTCATC		
		TGATAGCCCCGTACACGAAACGGCATA		
Putative Narna-like virus	p-narnav	TGTCCCTGAAGATTCATTTCGA	28.5/0.8	[2]
		CTATGTAAAGCCTCGTCGGTGAT		
		TCCTAGGTGATGATATAAT		
Piscine orthoreovirus 1a	prv-1	TGCTAACACTCCAGGAGTCATTG	25.4/1.0	[16]
		TGAATCCGCTGCAGATGAGTA		
		CGCCGGTAGCTCT		

Renibacterium salmoninarum	re_sal	CAACAGGGTGGTTATTCTGCTTTC	23.6/1.9	[17]
		CTATAAGAGCCACCAGCTGCAA		
		CTCCAGCGCCGCAGGAGGAC		
Candidatus Syngnamydia salmonis	sch	GGGTAGCCCGATATCTTCAAAGT	27.9/3.9	[6]
		CCCATGAGCCGCTCTCTCT		
		TCCTTCGGGACCTTAC		
Salmon pescarenavirus-1	spav-1	CCTGCCTCTTTGCTCATTGTG	26.9/3.0	[18]
		AGAAAAAGCTGTGGTACTTTAGAAAGC		
		ATCCGCCTAACGGTTGG		
Salmon pescarenavirus-2	spav-2	AACATGAAGGGCGATTCGTT	27.5/0.4	[18]
		CAGCCCGCGGACTGAGT		
		CAAGTGATGTAAGCTTG		
Tenacibaculum dicentrarchi	te_dic	TGCTTGCATAGATGACGACCG	27.4/1.7	modified from [19]
		GGGATAATCCTCTCAGACCCCC		
		AGTGCTTCGGCATCGRAAC		
Tenacibaculum finnmarkense	Te_fin	CAGAATTTAATTACGAAACGTTGGC	26.2/1.7	developed by Shaorong Li (MGL)
		CCTTCATCATCTGTGTTACGTTTATC		
		ACGCGTATGCGTGAATTGTCTT		
Tenacibaculum maritimum	te_mar	TGCCTTCTACAGAGGGATAGCC	26.7/1.9	[20]
		CTATCGTTGCCATGGTAAGCCG		
		CACTTTGGAATGGCATCG		
Viral hemorrhagic septicemia virus	vhsv	AAACTCGCAGGATGTGTGCGTCC	26.9/15.5	[21]
		TCTGCGATCTCAGTCAGGATGAA		
		TAGAGGGCCTTGGTGATCTTCTG		
Aliivibrio salmonicida	vi_sal	GTGTGATGACCGTTCCATATTT	21.5/1.9	[5]
		GCTATTGTCATCACTCTGTTTCTT		
		TCGCTTCATGTTGTGTAATTAGGAGCGA		
Yersinia ruckeri	ye_ruc	TCCAGCACCAAATACGAAGG	25.8/28.9	[1]
		ACATGGCAGAACGCAGATC		
		AAGGCGGTTACTTCCCGGTTCCC		
Salmo salar	Sasa	CGCCCTAAGTCTCTTGATTCGA	NA	[22]
		CGTTATAAATTTGGTCATCTCCCAGA		
		AGAACTCAGCCAGCCTG		
Oncorhynchus tshawytscha	Onts	CTGGCACMGGGTGAACAGTCTACC	NA	[23]
		AATGAAGGGAGAAGATCGTYAGATCA		
		CTAGCCCACGCAGGAG		

Oncorhynchus kisutch	Onki	CCTTGGTGGCGGATATACTTATCTTA	NA	[24]
		GAACTAGGAAGATGGCGAAGTAGATC		
		TGGAACACCCATTCAT		
Oncorhynchus gorbuscha	Ongo	CCCTAAACATGCTAGGGCTACTTCCA	NA	developed by Shaorong Li (MGL)
		GGTTCCTTCAGGCAAGAGGT		
		TGCCACAGTAATCATCGGC		
Oncorhynchus keta	Onke	CCCTAAACATACTGGGACTACTTCCA	NA	developed by Shaorong Li (MGL)
		GGTCCCCTCAGGCAAGAGAT		
		CGCCACAGTAATTATTGGT		
Oncorhynchus nerka	Onne	TCTGCCCTTCTCCTTACGATTTT	NA	[25]
		GTTCGACCTAGAAATCGCCCTT		
		CCATCCTGTTCCTCCT		
Oncorhynchus mykiss	Onmy	GGATGAACAGTATACCCCCCTCTA	NA	developed by Angela Schulze (MGL)
		TGAAGGGAGAAGATAGTTAAATCAACAG		
		CGGCAACCTCGCC		
Gadus macrocephalus	Pacifcicod	TTGTTTGCCTACGCCATCCT	NA	developed by Shaorong Li (MGL)
		TTGAAGTGTGGAGGAAGGGC		
		GTGGCGTACTTGCACTCCTA		
Clupea harengus / *Clupea pallasli*	Herring	CCCATTTGTGATTGCAGGGG	NA	[26]
		CTGAGTTAAGTCCTGCCGGG		
		TACTATTCTCCACCTTCTGTTCCTC		
Ammodytes personatus	Sandlance	GATTCTTGGAGCAATCAACTTCATC	NA	developed by Shaorong Li (MGL)
		AATAAGGGTGTCTGATACTGAGAGATAGC		
		CCACAATTATTAACATGAAACC		
Hypomesus pretiosus	surf Smelt	TTTGGCTCCCTCCTTGGACTA	NA	developed by Shaorong Li (MGL)
		CAGAAGAAAATGCCGTAGCAGTT		
		TCTTATTATTCAAATCCTCACAGGC		
Thaleichthys pacificus	Eulachon	GTTGATTTACCTGCGCCCTCTA	NA	developed by Shaorong Li (MGL)
		CCAGAAATAGGCCCGTAAGGA		
		ATCTCTGTCTGATGAAAC		
Lampetra spp	Lamprey	CTTTAGCAGCAGCCATCATA	NA	[27]
		GTAGTGCTAGATCAGCAATTAGAA		
		AACGTGGCATTCAATTTCGTCCGC		
Gasterosteus aculeatus	Stickleback	ACGCCACCTTAACACGTTTC	NA	[28]
		AGAGCCTGTCTGGTGAAGGA		
		CTGGTGCCACACTTGTTCAC		

Engraulis mordax	Anchovy	TTCACTTGGCATTTGACGGG	NA	[29]
		TGCTCCTGAGATCACTTATGC		
		AGGTTGAACATTTTCCTTGCTTGCGA		
Gadus chalcogrammus	Pollock	GCTAACCCCATCGTTACCCCT	NA	developed by Shaorong Li (MGL)
		ACAGGAGTGCAAGTACACCG		
		CCTACGCCATCTTACGCTCT		

References for Table S4

References

[1] Keeling, S. *et al.* Development and validation of a real-time pcr assay for the detection of a eromonas salmonicida. *Journal of Fish Diseases* **36**, 495–503 (2013).

[2] Mordecai, G. J. *et al.* Discovery and surveillance of viruses from salmon in British Columbia using viral immune-response biomarkers, metatranscriptomics, and high-throughput RT-PCR. *Virus evolution* **7**, veaa069 (2021).

[3] Mitchell, S. O. *et al.* '*Candidatus* Branchiomonas cysticola' is a common agent of epitheliocysts in seawater-farmed Atlantic salmon *Salmo salar* in Norway and Ireland. *Disease of Aquatic Organisms* **103**, 35–43 (2013).

[4] Purcell, M. K. *et al.* Identification of the major capsid protein of erythrocytic necrosis virus (env) and development of quantitative real-time pcr assays for quantification of env dna. *Journal of Veterinary Diagnostic Investigation* **28**, 382–391 (2016).

[5] Miller, K. M. *et al.* Report on the performance evaluation of the Fluidigm BioMark platform for high-throughput microbe monitoring in salmon. DFO Can. Sci. Advis. Sec. Res. Doc. 2016/038. xi + 282 p. (2016).

[6] Duesund, H., Nylund, S., Watanabe, K., Ottem, K. F. & Nylund, A. Characterization of a vhs virus genotype iii isolated from rainbow trout (oncorhychus mykiss) at a marine site on the west coast of norway. *Virology journal* **7**, 1–15 (2010).

[7] White, V. C., Morado, J. F., Crosson, L. M., Vadopalas, B. & Friedman, C. S. Development and validation of a quantitative pcr assay for ichthyophonus spp. *Diseases of Aquatic Organisms* **104**, 69–81 (2013).

[8] Isaksen, T. E., Karlsbakk, E., Repstad, O. & Nylund, A. Molecular tools for the detection and identification of ichthyobodo spp.(kinetoplastida), important fish parasites. *Parasitology International* **61**, 675–683 (2012).

[9] Funk, V. A. *et al.* Development and validation of an RNA-and DNA-based quantitative PCR assay for determination of *Kudoa thyrsites* infection levels in Atlantic salmon *Salmo salar*. *Diseases of aquatic organisms* **75**, 239–249 (2007).

[10] McBeath, A. J. *et al.* Development and application of real-time pcr for specific detection of lepeophtheirus salmonis and caligus elongatus larvae in scottish plankton samples. *Diseases of aquatic organisms* **73**, 141–150 (2006).

[11] Grove, S., Reitan, L., Lunder, T. & Colquhoun, D. Real-time pcr detection of moritella viscosa, the likely causal agent of winter-ulcer in atlantic salmon salmo salar and rainbow trout oncorhynchus mykiss. *Diseases of aquatic organisms* **82**, 105–109 (2008).

[12] Fringuelli, E., Gordon, A., Rodger, H., Welsh, M. & Graham, D. Detection of neoparamoeba perurans by duplex quantitative taqman real-time pcr in formalin-fixed, paraffin-embedded atlantic salmonid gill tissues. *Journal of Fish Diseases* **35**, 711–724 (2012).

[13] Jørgensen, A., Nylund, A., Nikolaisen, V., Alexandersen, S. & Karlsbakk, E. Real-time PCR detection of *Parvicapsula pseudobranchicola* (Myxozoa: Myxosporea) in wild salmonids in Norway. *Journal of Fish Diseases* **34**, 365–371 (2011).

[14] Nylund, S., Nylund, A., Watanabe, K., Arnesen, C. E. & Karlsbakk, E. *Paranucleospora theridion* n. gen., n. sp.(Microsporidia, Enterocytozoonidae) with a life cycle in the salmon louse (*Lepeophtheirus salmonis*, Copepoda) and Atlantic salmon (*Salmo salar*). *Journal of Eukaryotic Microbiology* **57**, 95–114 (2010).

[15] Corbeil, S., McColl, K. A. & Crane, M. S. J. Development of a taqman quantitative pcr assay for the identification of piscirickettsia salmonis. *Bulletin-European Association of Fish Pathologists* **23**, 95–101 (2003).

[16] Wiik-Nielsen, C., Ski, P.-M., Aunsmo, A. & Løvoll, M. Prevalence of viral rna from piscine reovirus and piscine myocarditis virus in atlantic salmon, salmo salar l., broodfish and progeny. *Journal of Fish Diseases* **35**, 169–171 (2012).

[17] Powell, M., Overturf, K., Hogge, C. & Johnson, K. Detection of renibacterium salmoninarum in chinook salmon, oncorhynchus tshawytscha (walbaum), using quantitative pcr. *Journal of Fish Diseases* **28**,

615–622 (2005).

[18] Mordecai, G. J. *et al.* Endangered wild salmon infected by newly discovered viruses. *eLife* **8** (2019).

[19] Nowlan, J. P., Lumsden, J. S. & Russell, S. Advancements in characterizing *Tenacibaculum* infections in canada. *Pathogens* **9**, 1029 (2020).

[20] Fringuelli, E. *et al.* Development of a quantitative real-time pcr for the detection of tenacibaculum maritimum and its application to field samples. *Journal of Fish Diseases* **35**, 579–590 (2012).

[21] Jonstrup, S. P., Kahns, S., Skall, H. F., Boutrup, T. S. & Olesen, N. J. Development and validation of a novel t aqman-based real-time rt-pcr assay suitable for demonstrating freedom from viral haemorrhagic septicaemia virus. *Journal of Fish Diseases* **36**, 9–23 (2013).

[22] Atkinson, S. *et al.* A quantitative pcr-based environmental dna assay for detecting atlantic salmon (salmo salar l.). *Aquatic Conservation: Marine and Freshwater Ecosystems* **28**, 1238–1243 (2018).

[23] Laramie, M. B., Pilliod, D. S. & Goldberg, C. S. Characterizing the distribution of an endangered salmonid using environmental dna analysis. *Biological Conservation* **183**, 29–37 (2015).

[24] Pilliod, D. S. & Laramie, M. B. Salmon redd identification using environmental dna (edna). Tech. Rep., US Geological Survey (2016).

[25] Tillotson, M. D. *et al.* Concentrations of environmental dna (edna) reflect spawning salmon abundance at fine spatial and temporal scales. *Biological Conservation* **220**, 1–11 (2018).

[26] Knudsen, S. W. *et al.* Species-specific detection and quantification of environmental dna from marine fishes in the baltic sea. *Journal of experimental marine biology and ecology* **510**, 31–45 (2019).

[27] Ostberg, C. O., Chase, D. M., Hayes, M. C. & Duda, J. J. Distribution and seasonal differences in pacific lamprey and lampetra spp edna across 18 puget sound watersheds. *PeerJ* **6**, e4496 (2018).

[28] Thomsen, P. F. *et al.* Detection of a diverse marine fish fauna using environmental dna from seawater samples. (2012).

[29] Sassoubre, L. M., Yamahara, K. M., Gardner, L. D., Block, B. A. & Boehm, A. B. Quantification of environmental dna (edna) shedding and decay rates for three marine fish. *Environmental science & technology* **50**, 10456–10464 (2016).

Figures

Figures S1-S28: The following plots present raw data for each infectious agent showing cycle threshold (lower number indicates greater abundance) for the agent in water (red points) and in Atlantic salmon tissue (green points). Chinook salmon eDNA detections (blue points) are shown to indicate when infectious agent eDNA detections overlap with Chinook detections. Points positioned beneath 40 (max Ct) in the plots indicate samples were collected but the target nucleic acid was not detected. Blue vertical dashed lines indicate *S. salar* stocking dates and orange vertical dashed lines indicate harvest dates (thus farms are fallow after orange lines). Note that Cypress Harbour was a broodstock facility so that occasional, but never complete, harvest occurred and Doctor Islets was the only farm to be harvested and then restocked during the study period (fallow from November 2021 to March 2022). The abbreviation for the agent name is visible in the figure legend and abbreviations can be found in Table 1 or Table S2.

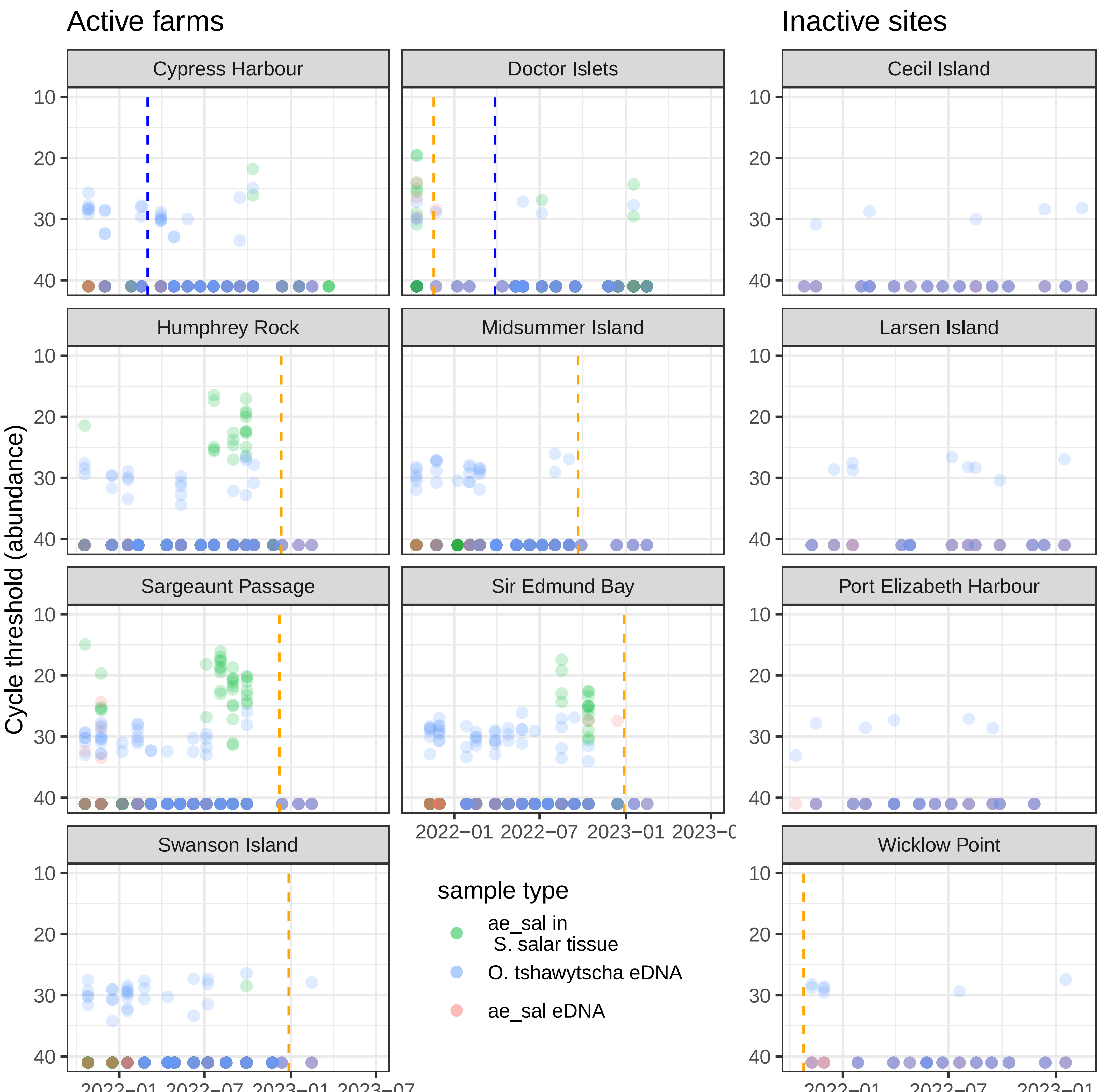

Active farms
Inactive sites
Cypress Harbour
Doctor Islets
Cecil Island
Humphrey Rock
Midsummer Island
Larsen Island
Sargeaunt Passage
Sir Edmund Bay
Port Elizabeth Harbour
Swanson Island
Wicklow Point
Cycle threshold (abundance)
10
20
30
40
2022−01
2022−07
2023−01
2023−07
sample type
ae_sal in S. salar tissue
O. tshawytscha eDNA
ae_sal eDNA

Figure S1

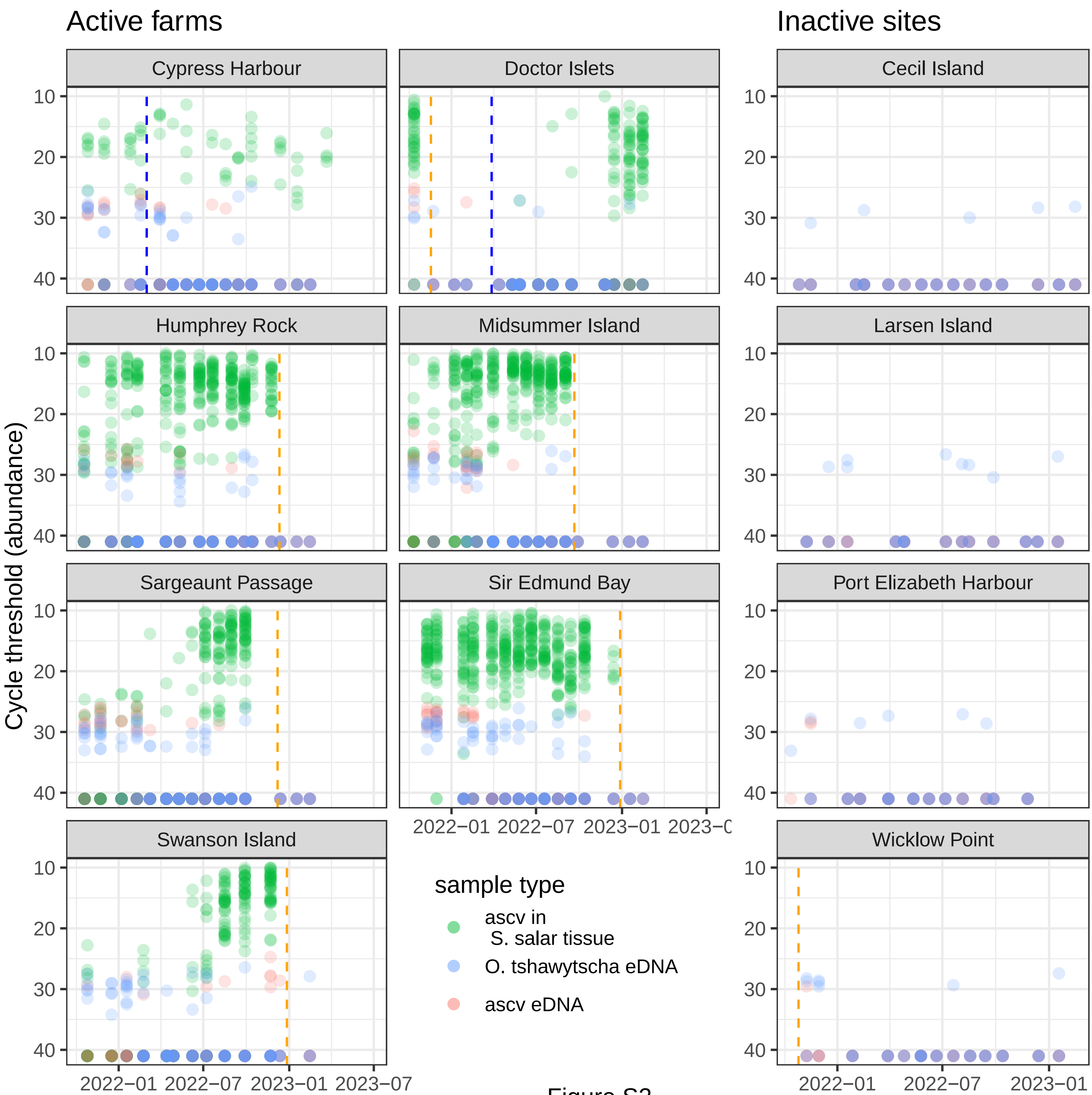

Active farms
Inactive sites
Cypress Harbour
Doctor Islets
Cecil Island
Humphrey Rock
Midsummer Island
Larsen Island
Sargeaunt Passage
Sir Edmund Bay
Port Elizabeth Harbour
Swanson Island
Wicklow Point
Cycle threshold (abundance)
10
20
30
40
2022−01
2022−07
2023−01
2023−07
sample type
ascv in S. salar tissue
O. tshawytscha eDNA
ascv eDNA

Figure S2

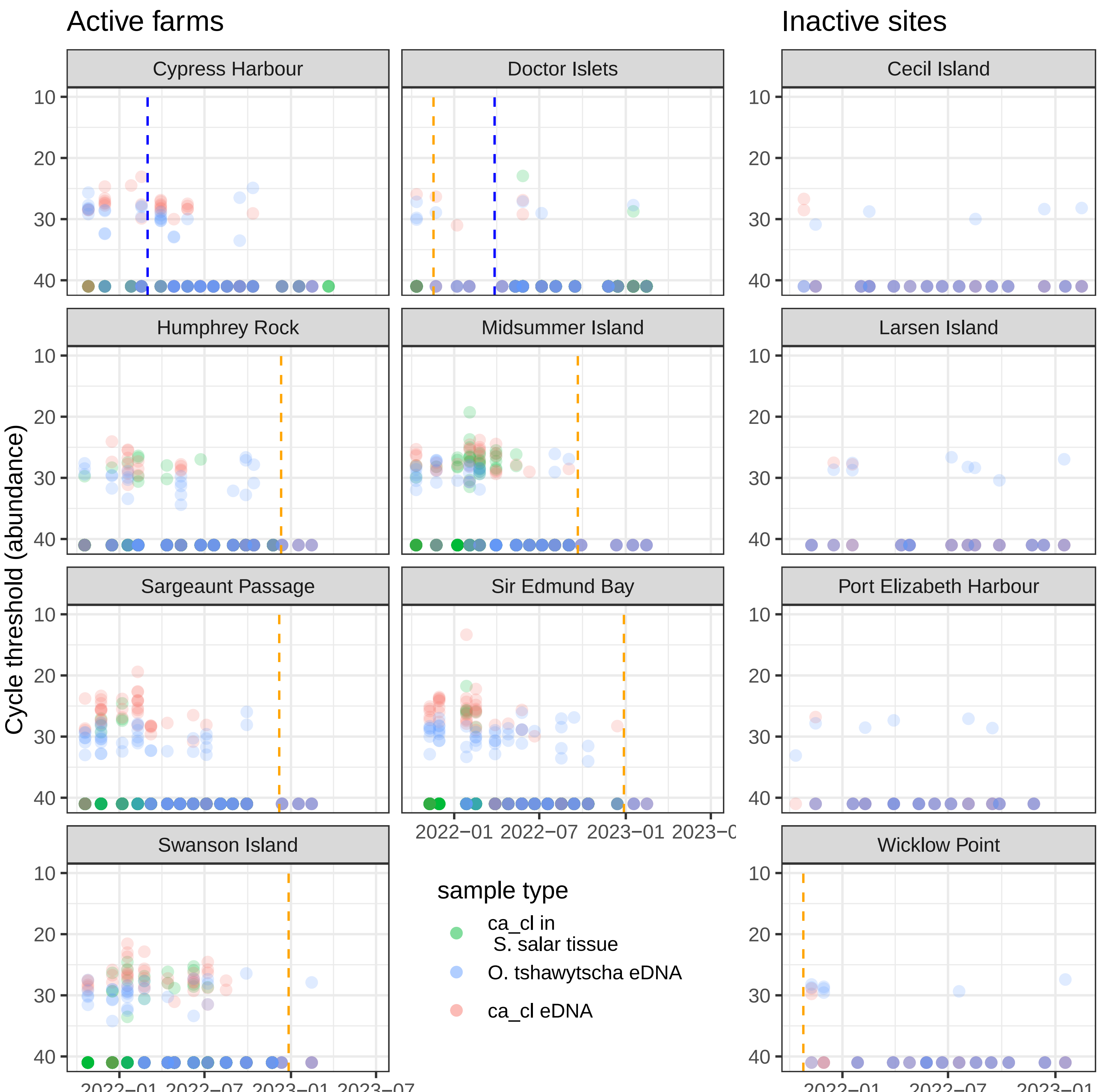

Active farms
Inactive sites
Cypress Harbour
Doctor Islets
Cecil Island
Humphrey Rock
Midsummer Island
Larsen Island
Sargeaunt Passage
Sir Edmund Bay
Port Elizabeth Harbour
Swanson Island
Wicklow Point
Cycle threshold (abundance)
10
20
30
40
2022−01
2022−07
2023−01
2023−07
sample type
ca_cl in S. salar tissue
O. tshawytscha eDNA
ca_cl eDNA

Figure S3

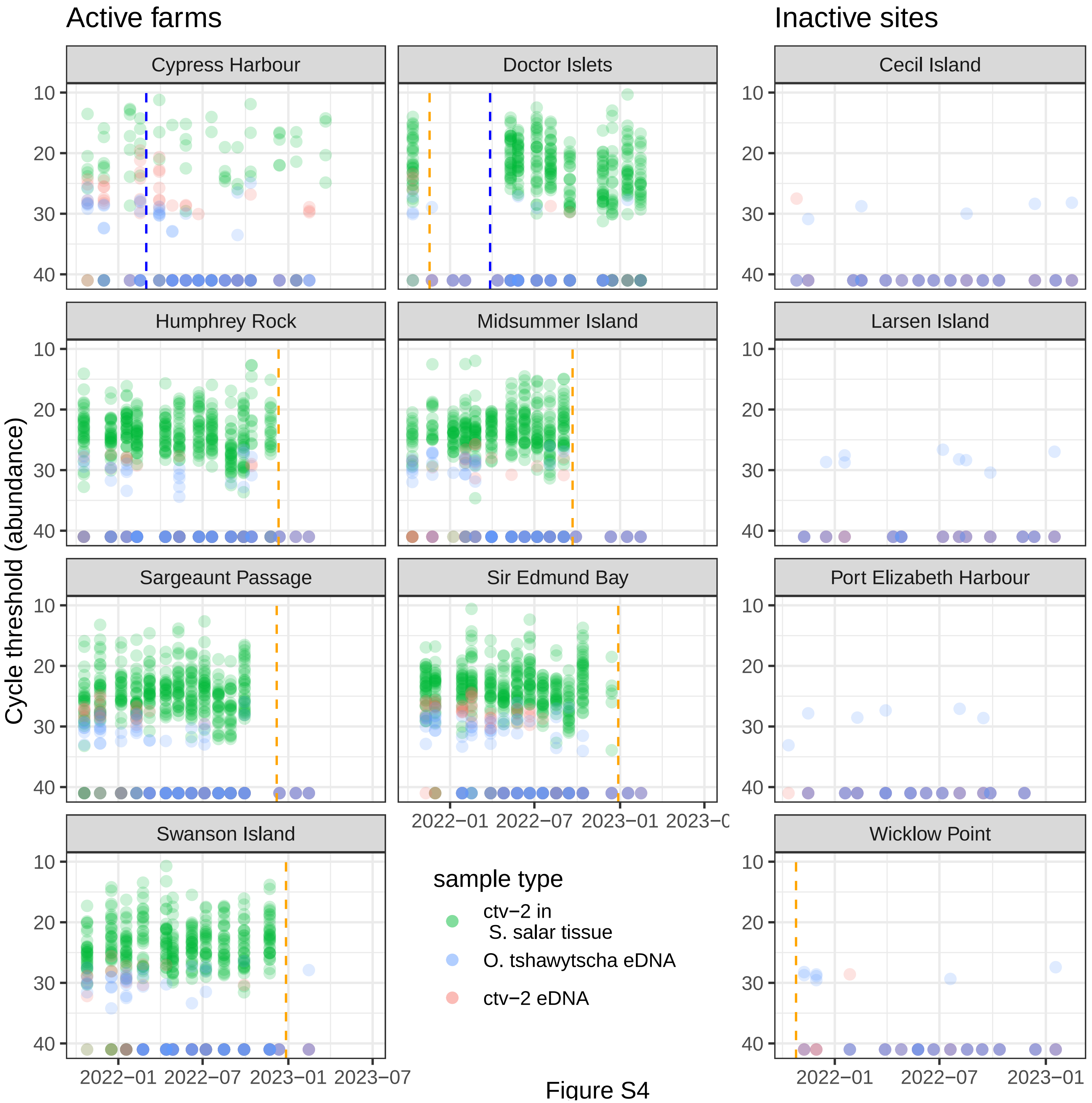
Active farms
Inactive sites
Cypress Harbour
Doctor Islets
Cecil Island
Humphrey Rock
Midsummer Island
Larsen Island
Sargeaunt Passage
Sir Edmund Bay
Port Elizabeth Harbour
Swanson Island
Wicklow Point
Cycle threshold (abundance)
10
20
30
40
2022−01
2022−07
2023−01
2023−07
sample type
ctv−2 in
S. salar tissue
O. tshawytscha eDNA
ctv−2 eDNA

Figure S4

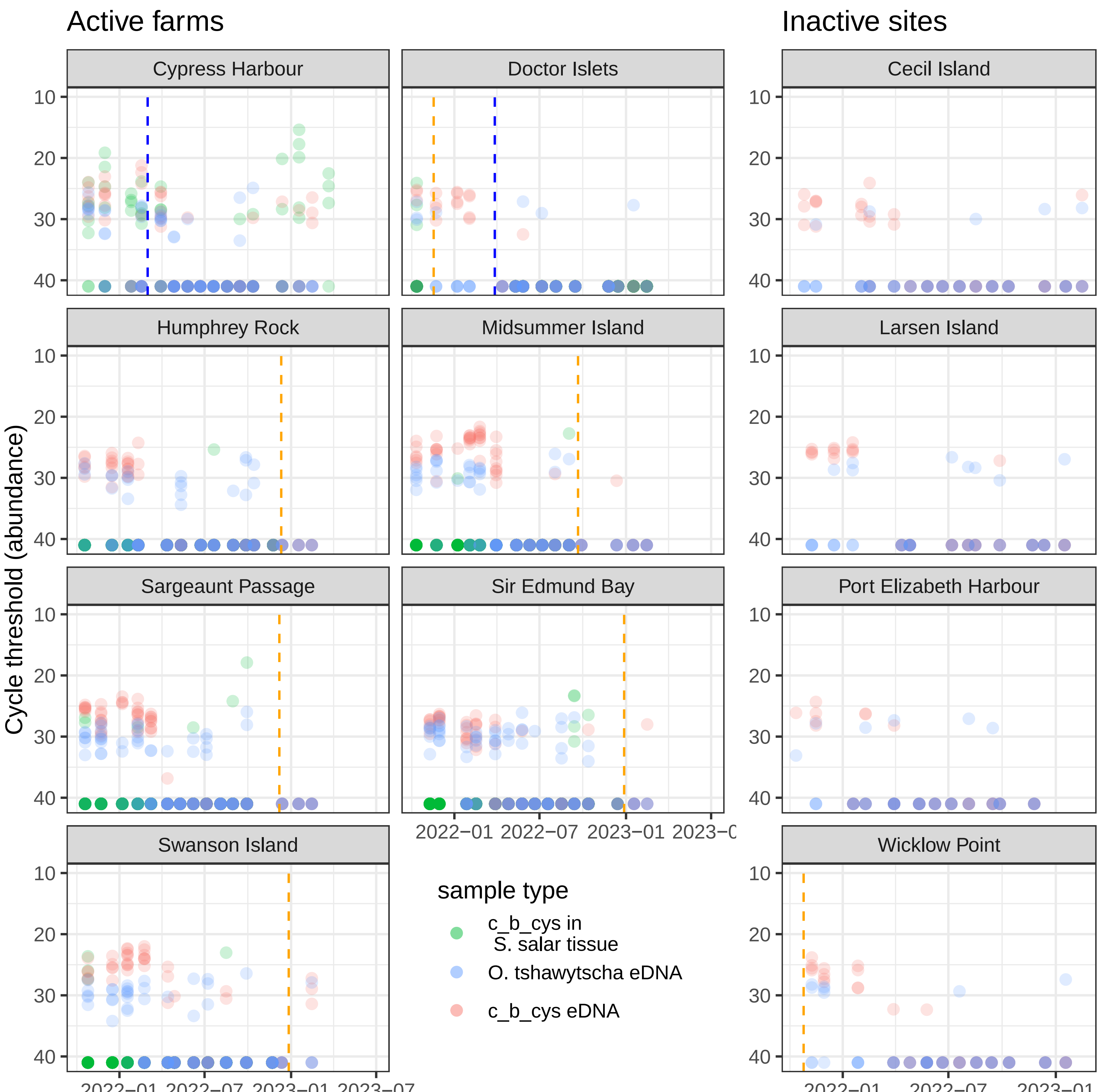
Active farms
Inactive sites
Cypress Harbour
Doctor Islets
Cecil Island
Humphrey Rock
Midsummer Island
Larsen Island
Sargeaunt Passage
Sir Edmund Bay
Port Elizabeth Harbour
Swanson Island
Wicklow Point
Cycle threshold (abundance)
10
20
30
40
2022−01
2022−07
2023−01
2023−07
sample type
c_b_cys in S. salar tissue
O. tshawytscha eDNA
c_b_cys eDNA

Figure S5

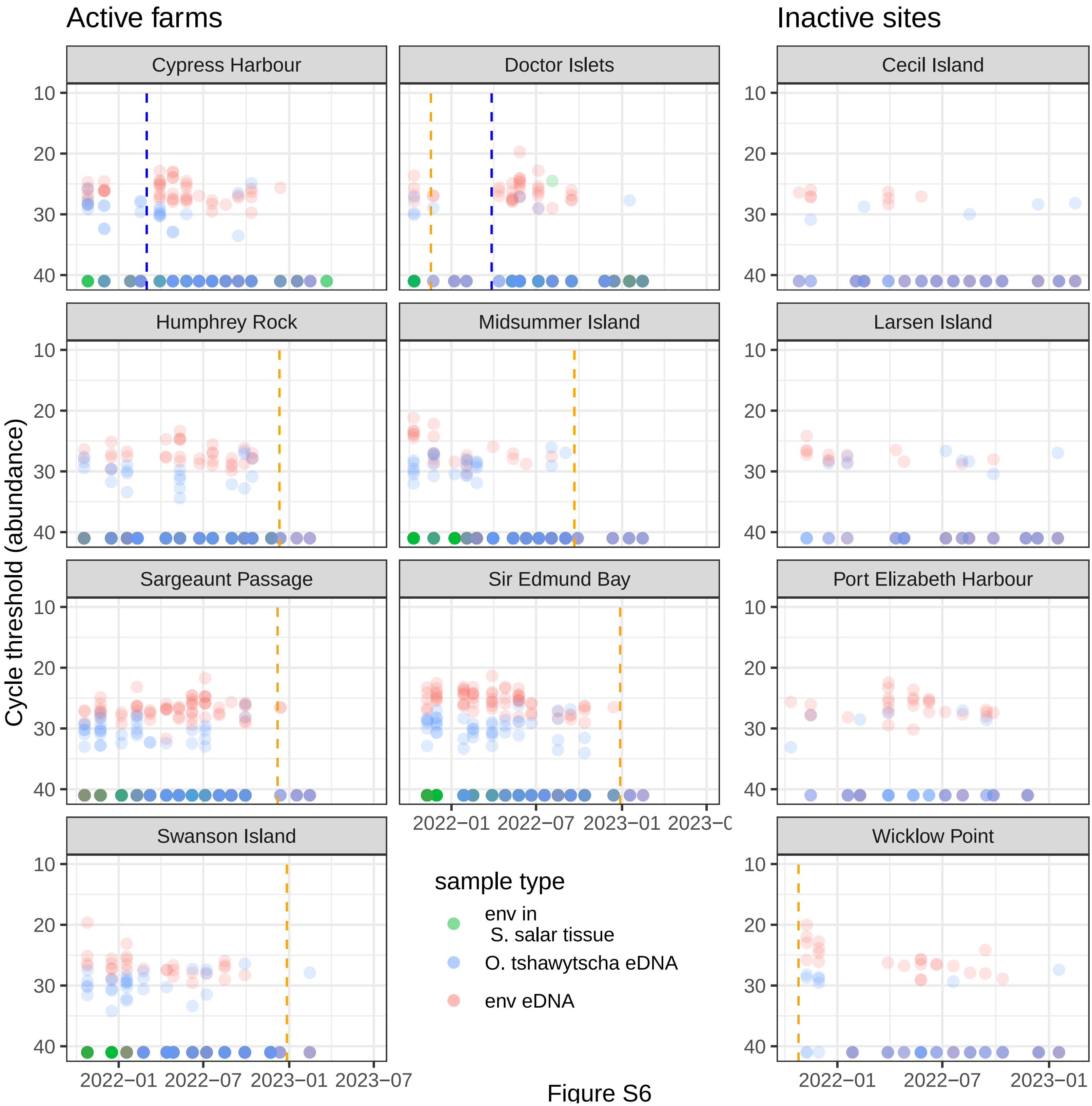
Active farms
Inactive sites
Cypress Harbour
Doctor Islets
Cecil Island
Humphrey Rock
Midsummer Island
Larsen Island
Sargeaunt Passage
Sir Edmund Bay
Port Elizabeth Harbour
Swanson Island
Wicklow Point
Cycle threshold (abundance)
10
20
30
40
2022−01
2022−07
2023−01
2023−07
sample type
env in
S. salar tissue
O. tshawytscha eDNA
env eDNA

Figure S6

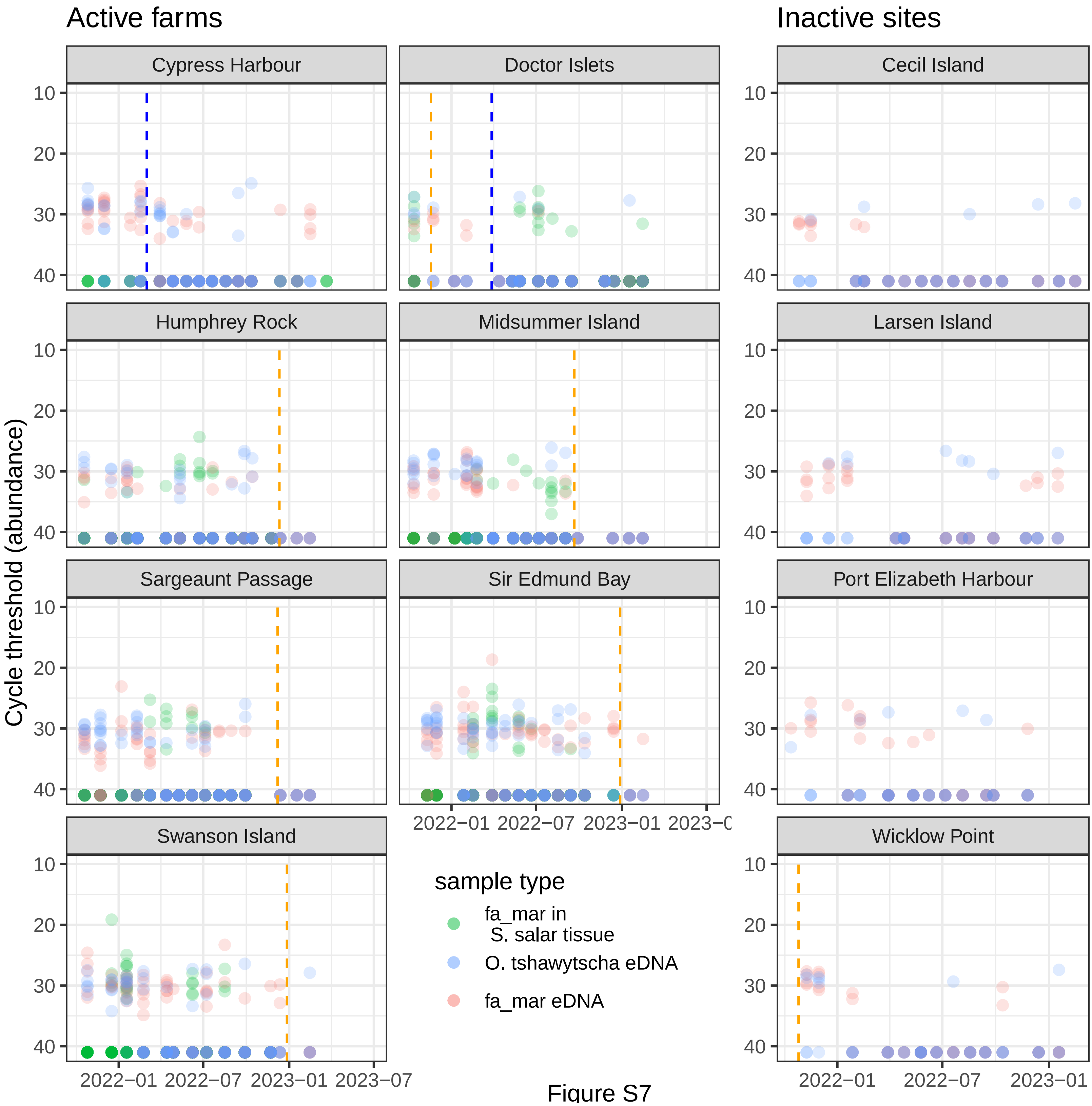
Active farms
Inactive sites
Cypress Harbour
Doctor Islets
Cecil Island
Humphrey Rock
Midsummer Island
Larsen Island
Sargeaunt Passage
Sir Edmund Bay
Port Elizabeth Harbour
Swanson Island
Wicklow Point
Cycle threshold (abundance)
10
20
30
40
2022−01
2022−07
2023−01
2023−07
sample type
fa_mar in S. salar tissue
O. tshawytscha eDNA
fa_mar eDNA

Figure S7

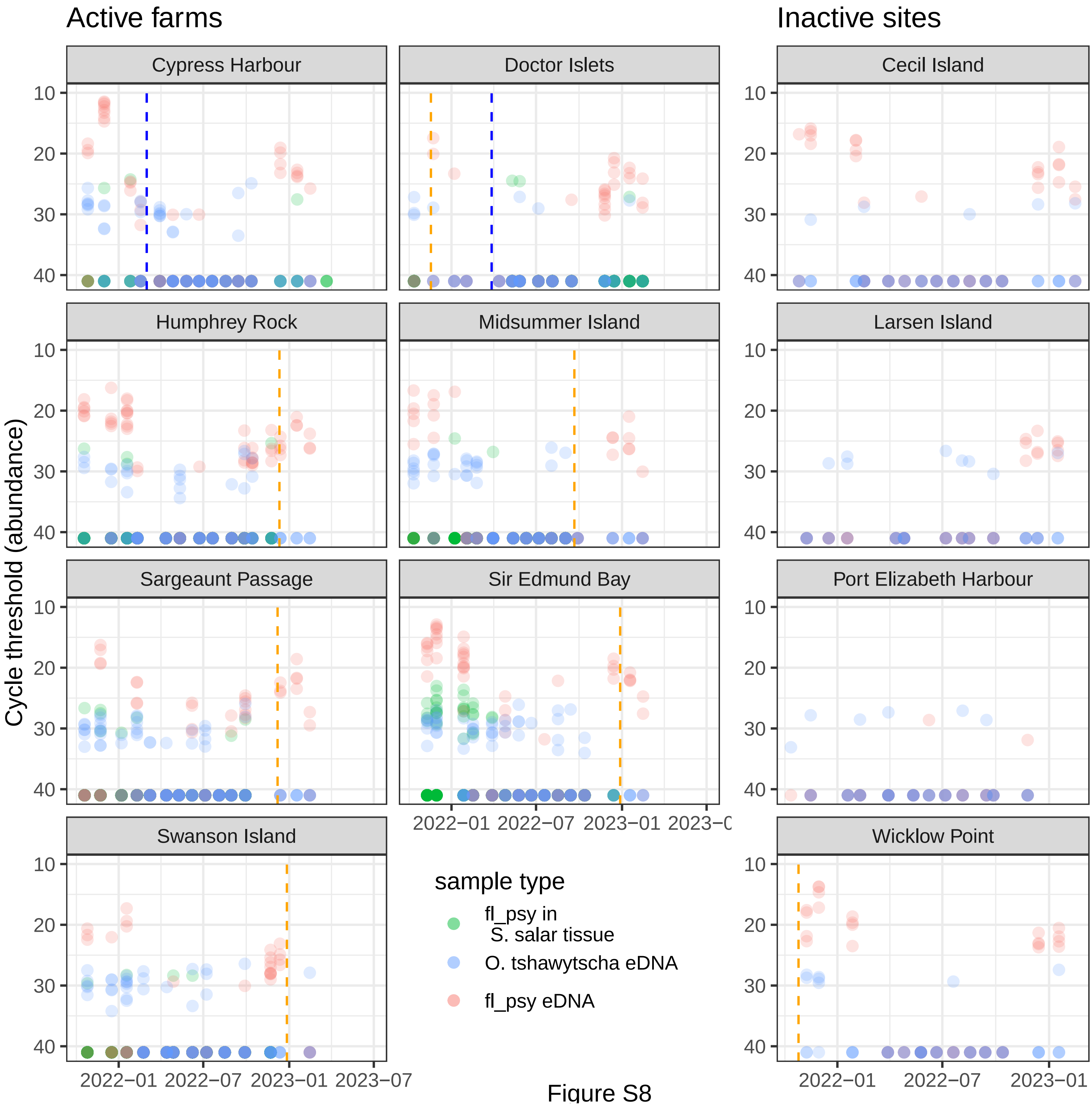

Active farms
Inactive sites
Cypress Harbour
Doctor Islets
Cecil Island
Humphrey Rock
Midsummer Island
Larsen Island
Sargeaunt Passage
Sir Edmund Bay
Port Elizabeth Harbour
Swanson Island
Wicklow Point
Cycle threshold (abundance)
10
20
30
40
2022−01
2022−07
2023−01
2023−07
sample type
fl_psy in S. salar tissue
O. tshawytscha eDNA
fl_psy eDNA

Figure S8

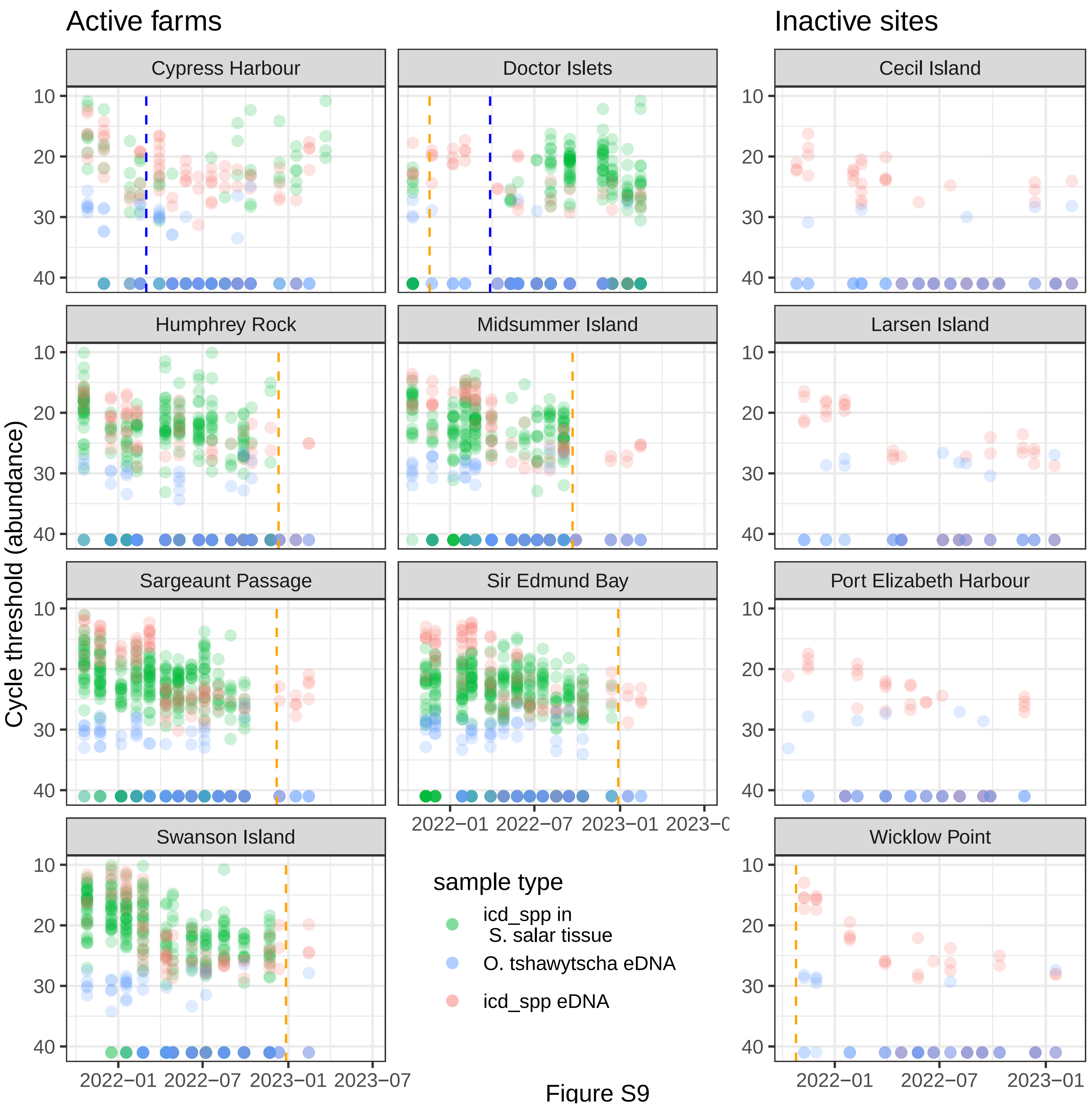

Active farms
Inactive sites
Cypress Harbour
Doctor Islets
Cecil Island
Humphrey Rock
Midsummer Island
Larsen Island
Sargeaunt Passage
Sir Edmund Bay
Port Elizabeth Harbour
Swanson Island
Wicklow Point
Cycle threshold (abundance)
10
20
30
40
2022−01
2022−07
2023−01
2023−07
sample type
icd_spp in S. salar tissue
O. tshawytscha eDNA
icd_spp eDNA

Figure S9

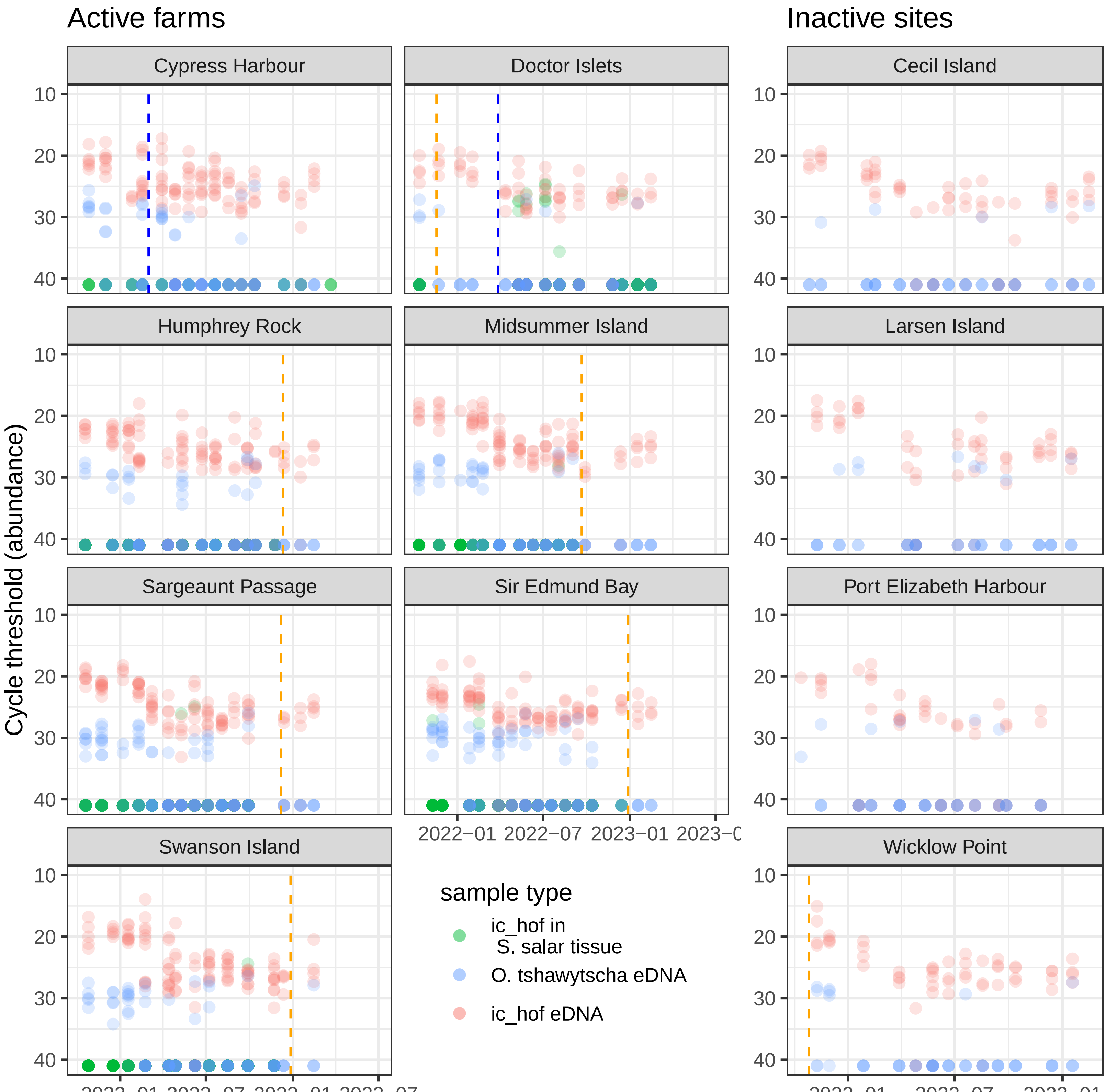
Active farms
Inactive sites
Cypress Harbour
Doctor Islets
Cecil Island
Humphrey Rock
Midsummer Island
Larsen Island
Sargeaunt Passage
Sir Edmund Bay
Port Elizabeth Harbour
Swanson Island
Wicklow Point
Cycle threshold (abundance)
10
20
30
40
2022−01
2022−07
2023−01
2023−07
sample type
ic_hof in
S. salar tissue
O. tshawytscha eDNA
ic_hof eDNA

Figure S10

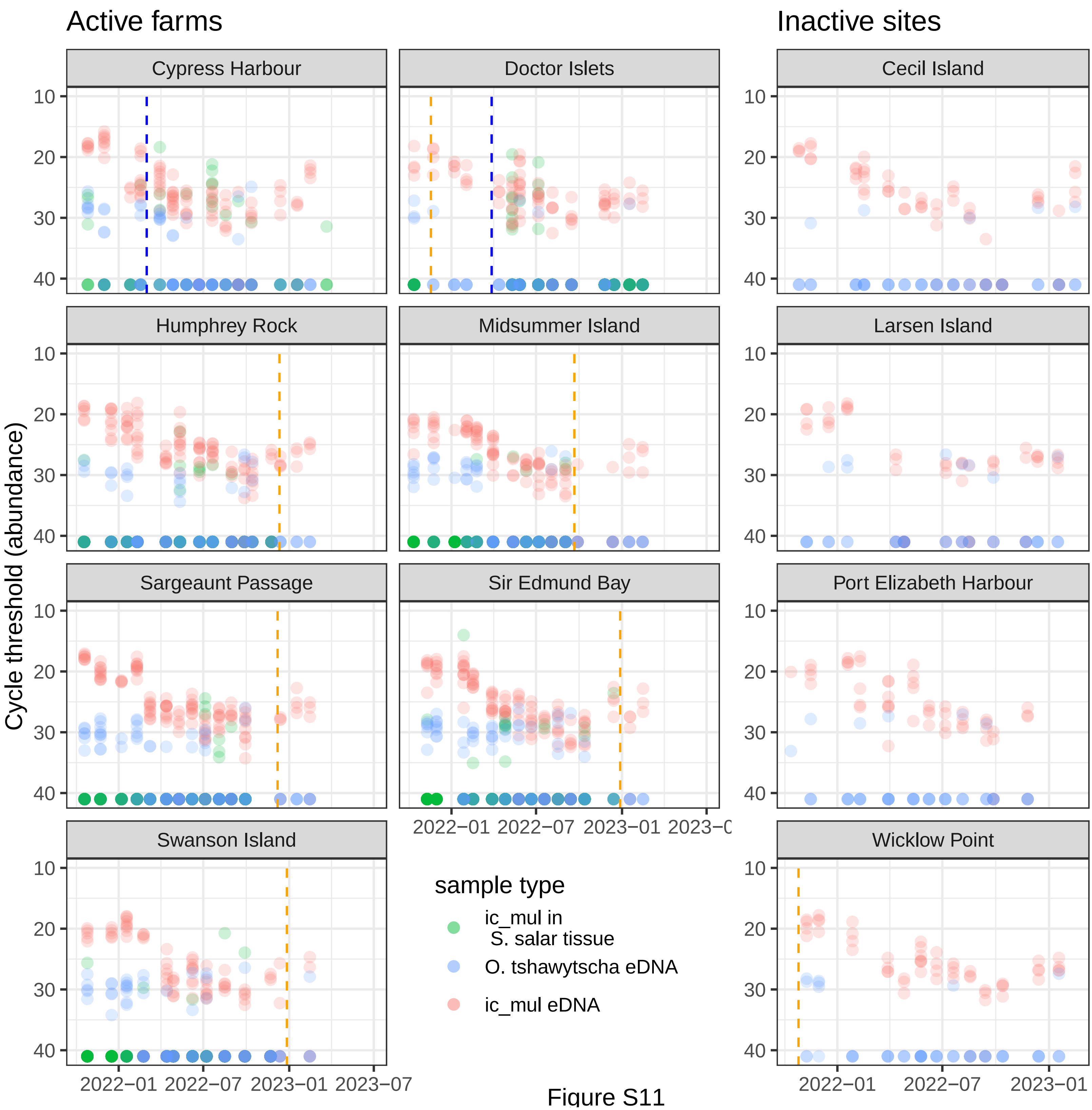

Active farms
Inactive sites
Cypress Harbour
Doctor Islets
Cecil Island
Humphrey Rock
Midsummer Island
Larsen Island
Sargeaunt Passage
Sir Edmund Bay
Port Elizabeth Harbour
Swanson Island
Wicklow Point
Cycle threshold (abundance)
10
20
30
40
2022−01
2022−07
2023−01
2023−07
sample type
ic_mul in
S. salar tissue
O. tshawytscha eDNA
ic_mul eDNA

Figure S11

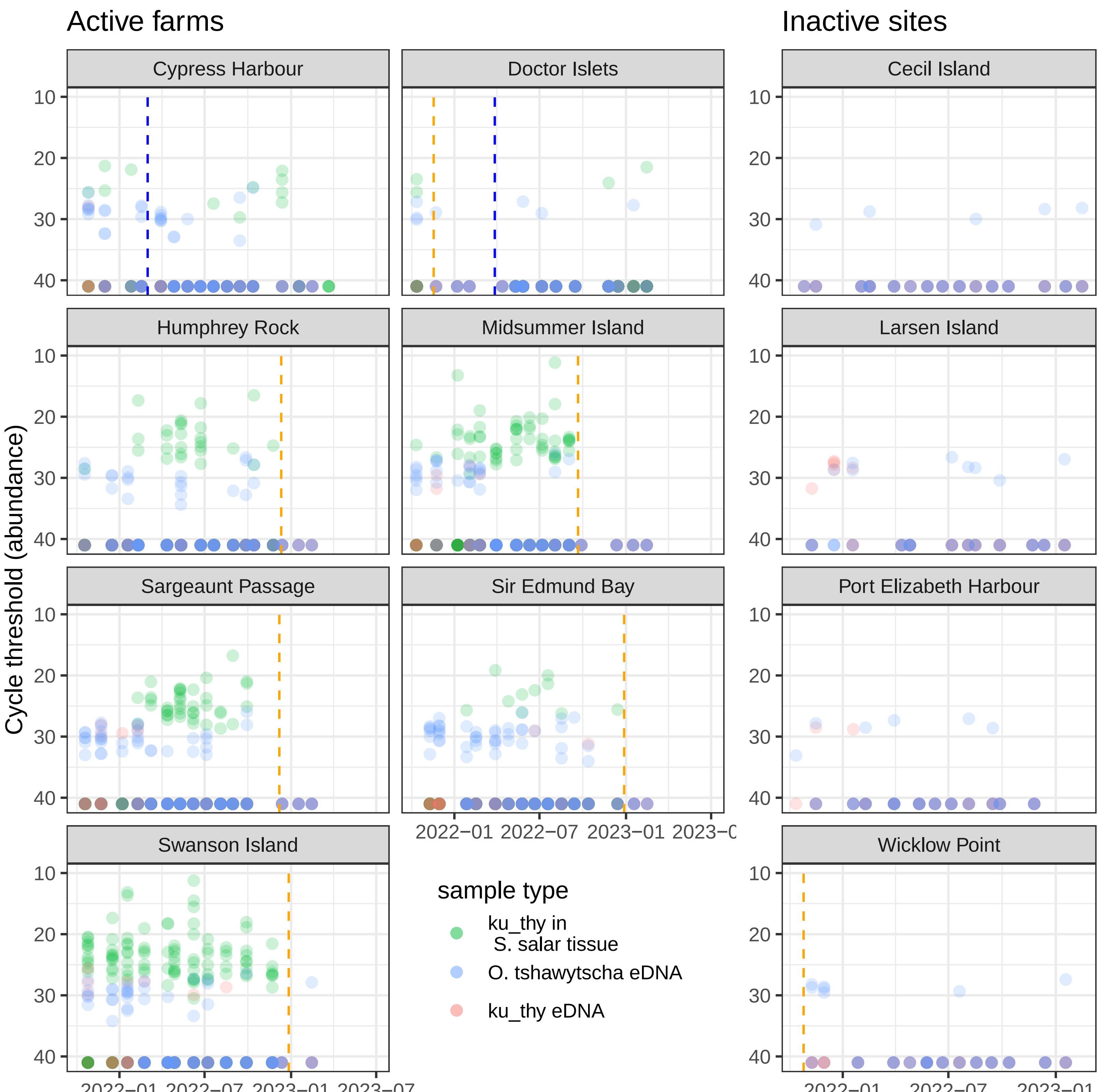

Active farms
Inactive sites
Cypress Harbour
Doctor Islets
Cecil Island
Humphrey Rock
Midsummer Island
Larsen Island
Sargeaunt Passage
Sir Edmund Bay
Port Elizabeth Harbour
Swanson Island
Wicklow Point
Cycle threshold (abundance)
10
20
30
40
2022−01
2022−07
2023−01
2023−07
sample type
ku_thy in S. salar tissue
O. tshawytscha eDNA
ku_thy eDNA

Figure S12

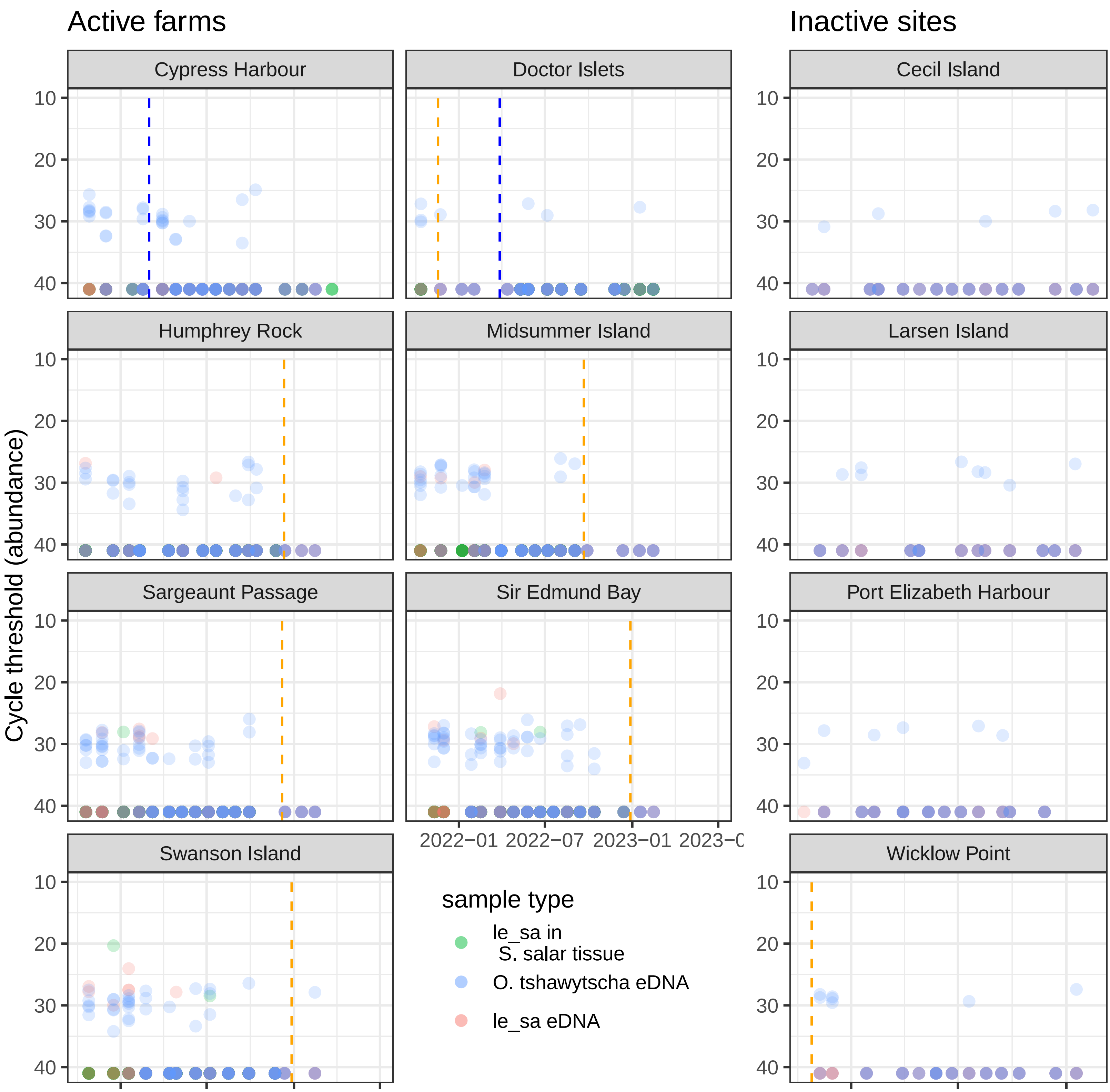
Active farms
Inactive sites
Cypress Harbour
Doctor Islets
Cecil Island
Humphrey Rock
Midsummer Island
Larsen Island
Sargeaunt Passage
Sir Edmund Bay
Port Elizabeth Harbour
Swanson Island
Wicklow Point
Cycle threshold (abundance)
10
20
30
40
2022−01
2022−07
2023−01
2023−07
sample type
le_sa in
S. salar tissue
O. tshawytscha eDNA
le_sa eDNA

Figure S13

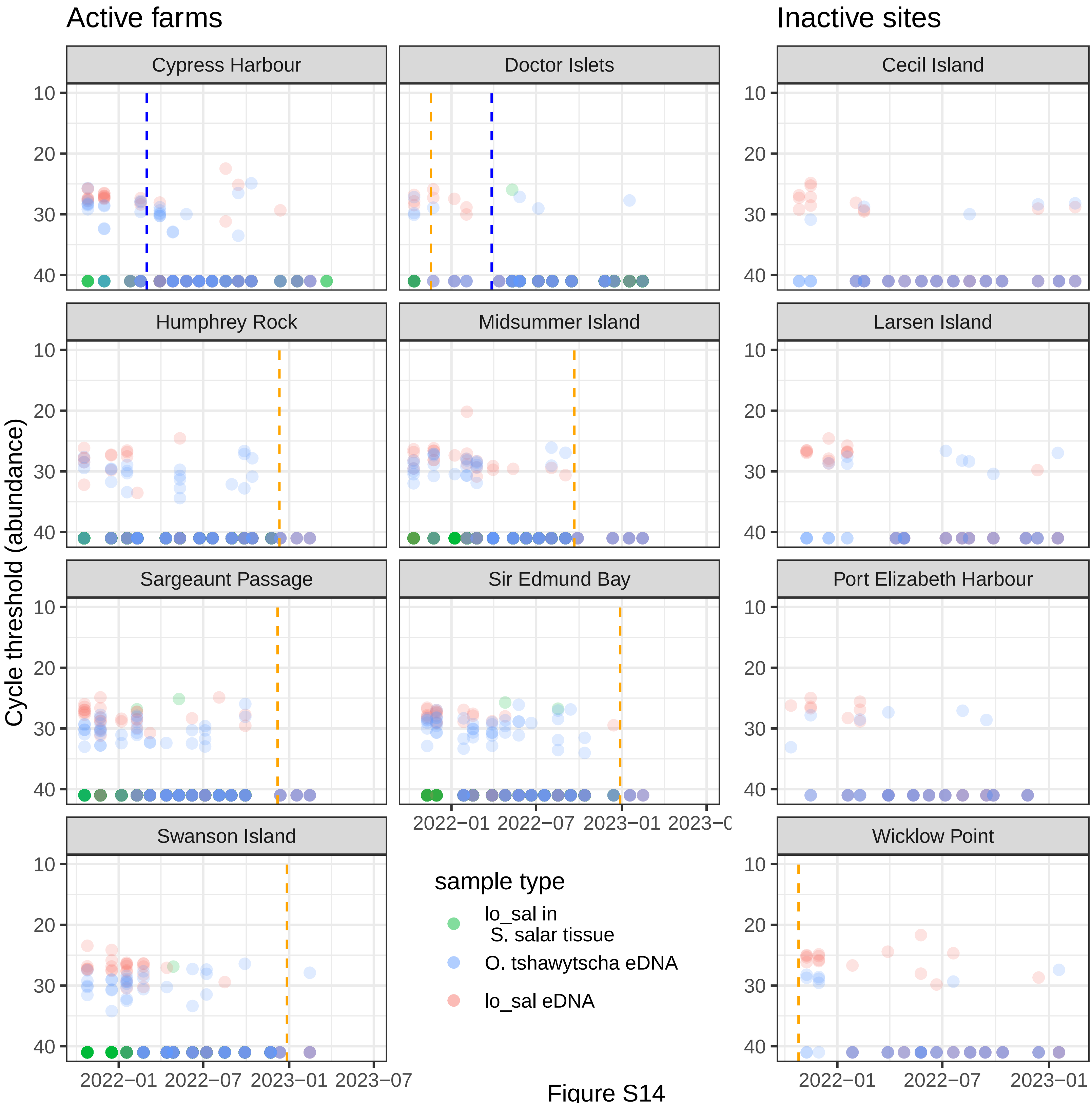

Active farms
Inactive sites
Cypress Harbour
Doctor Islets
Cecil Island
Humphrey Rock
Midsummer Island
Larsen Island
Sargeaunt Passage
Sir Edmund Bay
Port Elizabeth Harbour
Swanson Island
Wicklow Point
Cycle threshold (abundance)
10
20
30
40
2022−01
2022−07
2023−01
2023−07
sample type
lo_sal in
S. salar tissue
O. tshawytscha eDNA
lo_sal eDNA

Figure S14

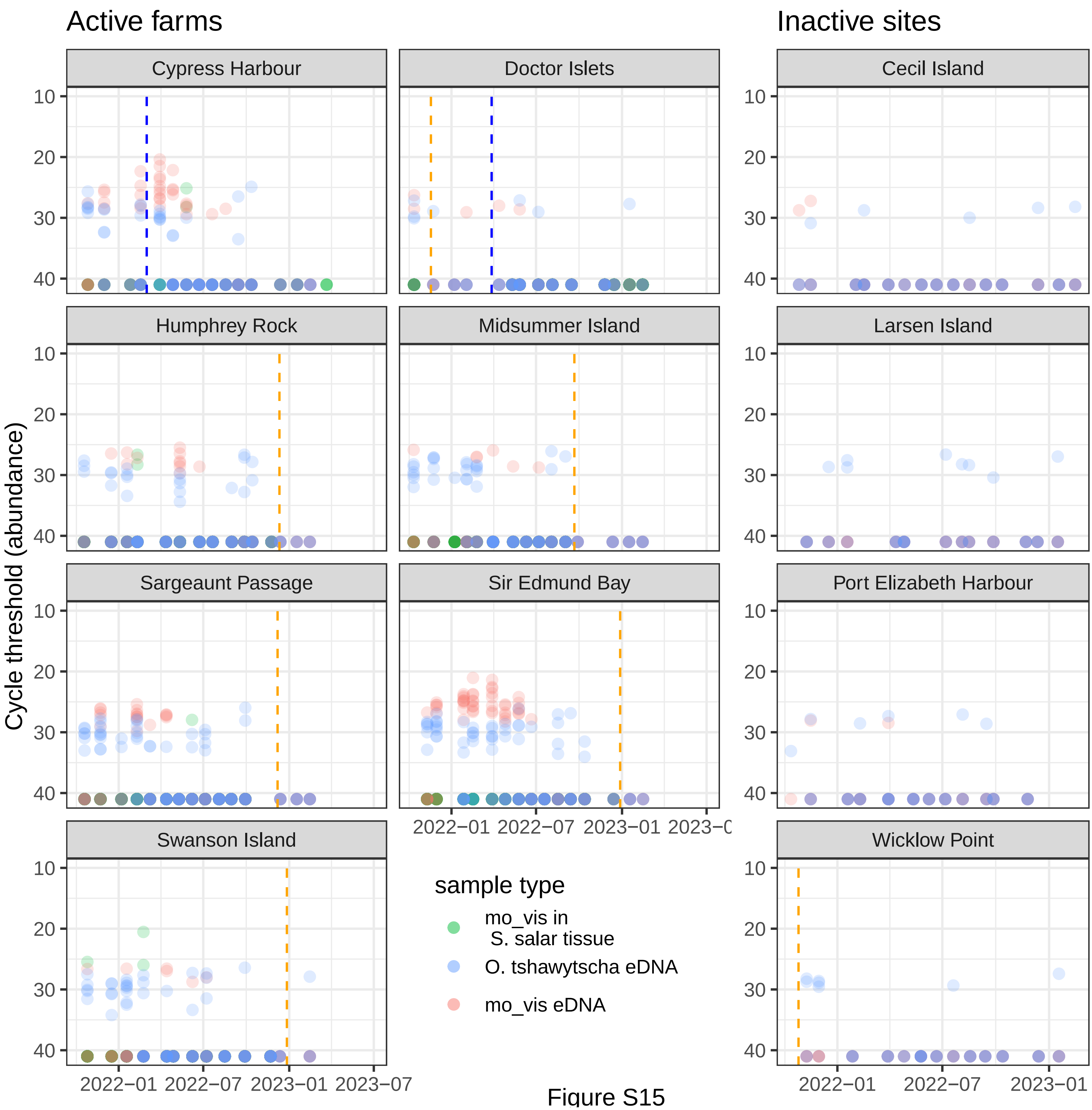

Active farms
Inactive sites
Cypress Harbour
Doctor Islets
Cecil Island
Humphrey Rock
Midsummer Island
Larsen Island
Sargeaunt Passage
Sir Edmund Bay
Port Elizabeth Harbour
Swanson Island
Wicklow Point
Cycle threshold (abundance)
10
20
30
40
2022−01
2022−07
2023−01
2023−07
sample type
mo_vis in
S. salar tissue
O. tshawytscha eDNA
mo_vis eDNA

Figure S15

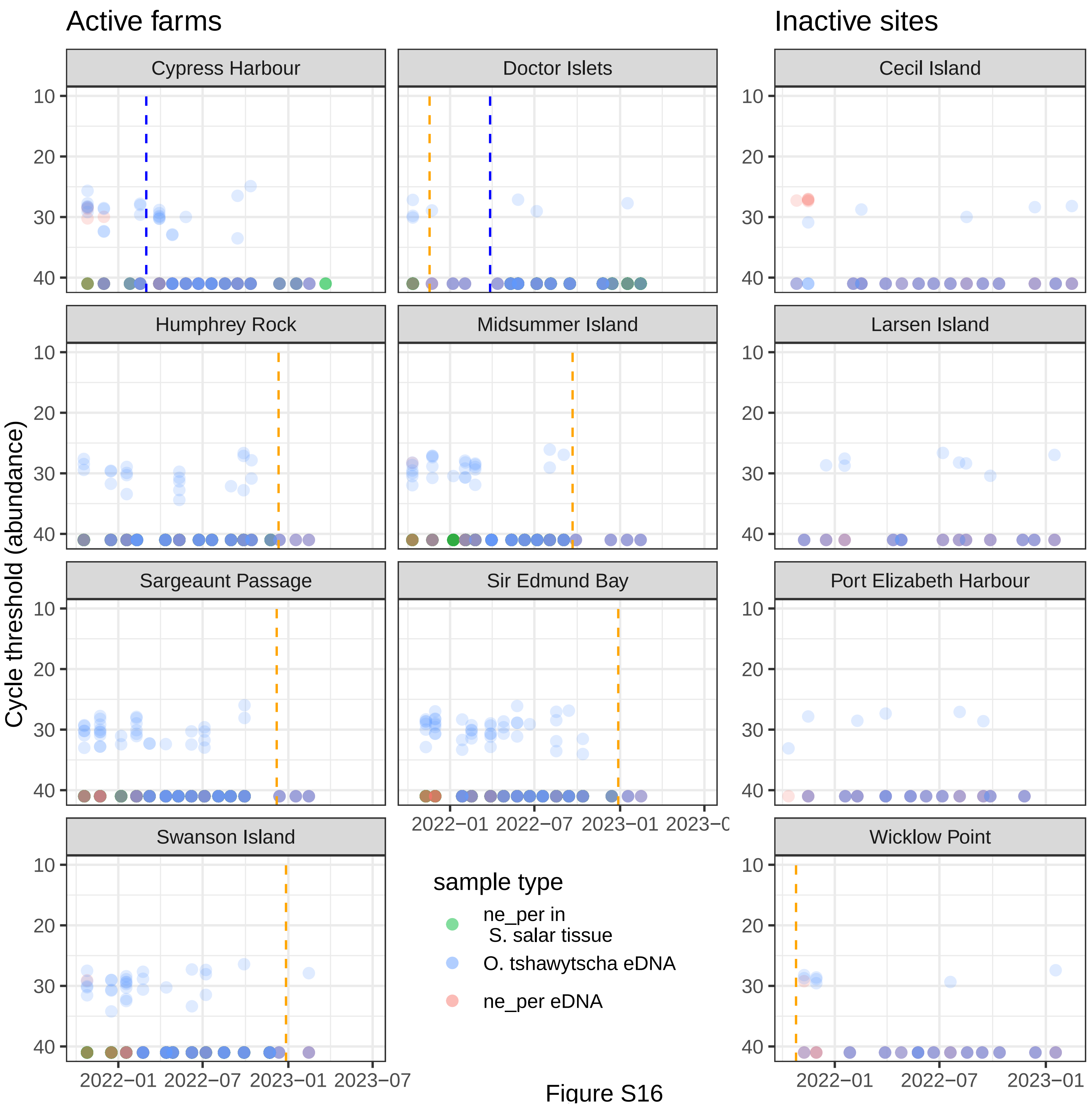
Active farms
Inactive sites
Cypress Harbour
Doctor Islets
Cecil Island
Humphrey Rock
Midsummer Island
Larsen Island
Sargeaunt Passage
Sir Edmund Bay
Port Elizabeth Harbour
Swanson Island
Wicklow Point
Cycle threshold (abundance)
10
20
30
40
2022−01
2022−07
2023−01
2023−07
sample type
ne_per in
S. salar tissue
O. tshawytscha eDNA
ne_per eDNA

Figure S16

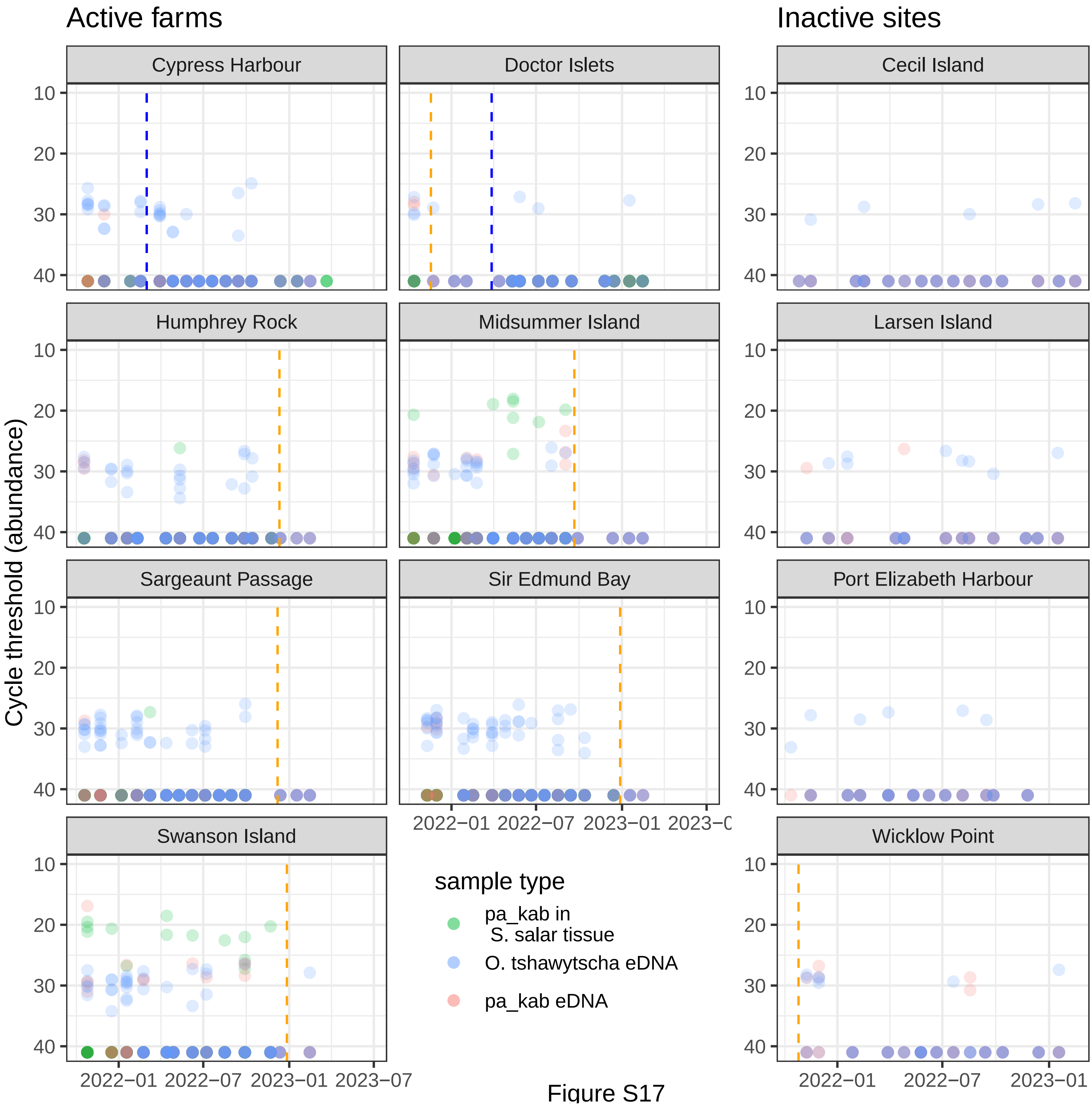
Active farms
Inactive sites
Cypress Harbour
Doctor Islets
Cecil Island
Humphrey Rock
Midsummer Island
Larsen Island
Sargeaunt Passage
Sir Edmund Bay
Port Elizabeth Harbour
Swanson Island
Wicklow Point
Cycle threshold (abundance)
10
20
30
40
2022−01
2022−07
2023−01
2023−07
sample type
pa_kab in S. salar tissue
O. tshawytscha eDNA
pa_kab eDNA

Figure S17

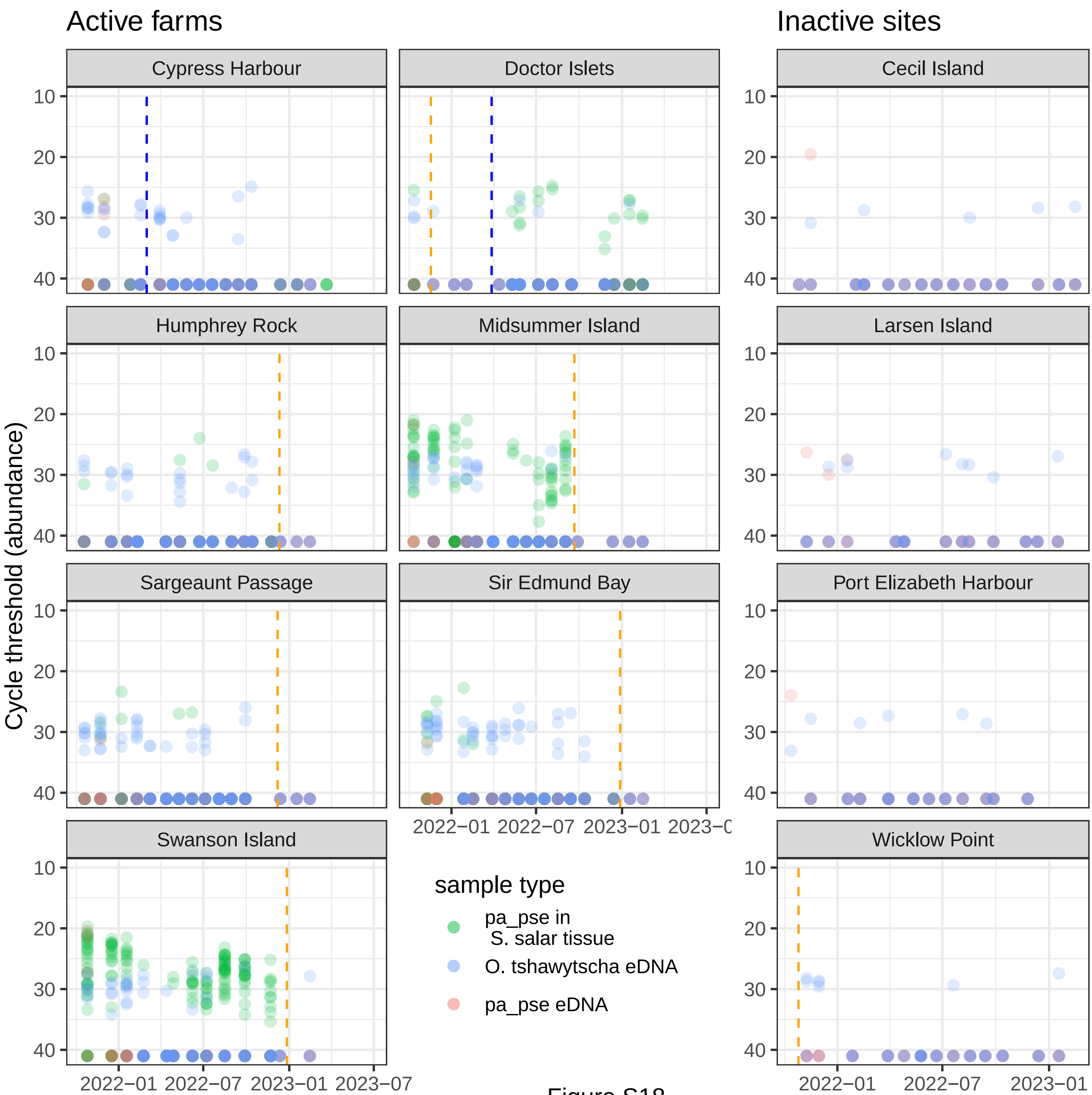
Active farms
Inactive sites
Cypress Harbour
Doctor Islets
Cecil Island
Humphrey Rock
Midsummer Island
Larsen Island
Sargeaunt Passage
Sir Edmund Bay
Port Elizabeth Harbour
Swanson Island
Wicklow Point
Cycle threshold (abundance)
10
20
30
40
2022−01
2022−07
2023−01
2023−07
sample type
pa_pse in S. salar tissue
O. tshawytscha eDNA
pa_pse eDNA

Figure S18

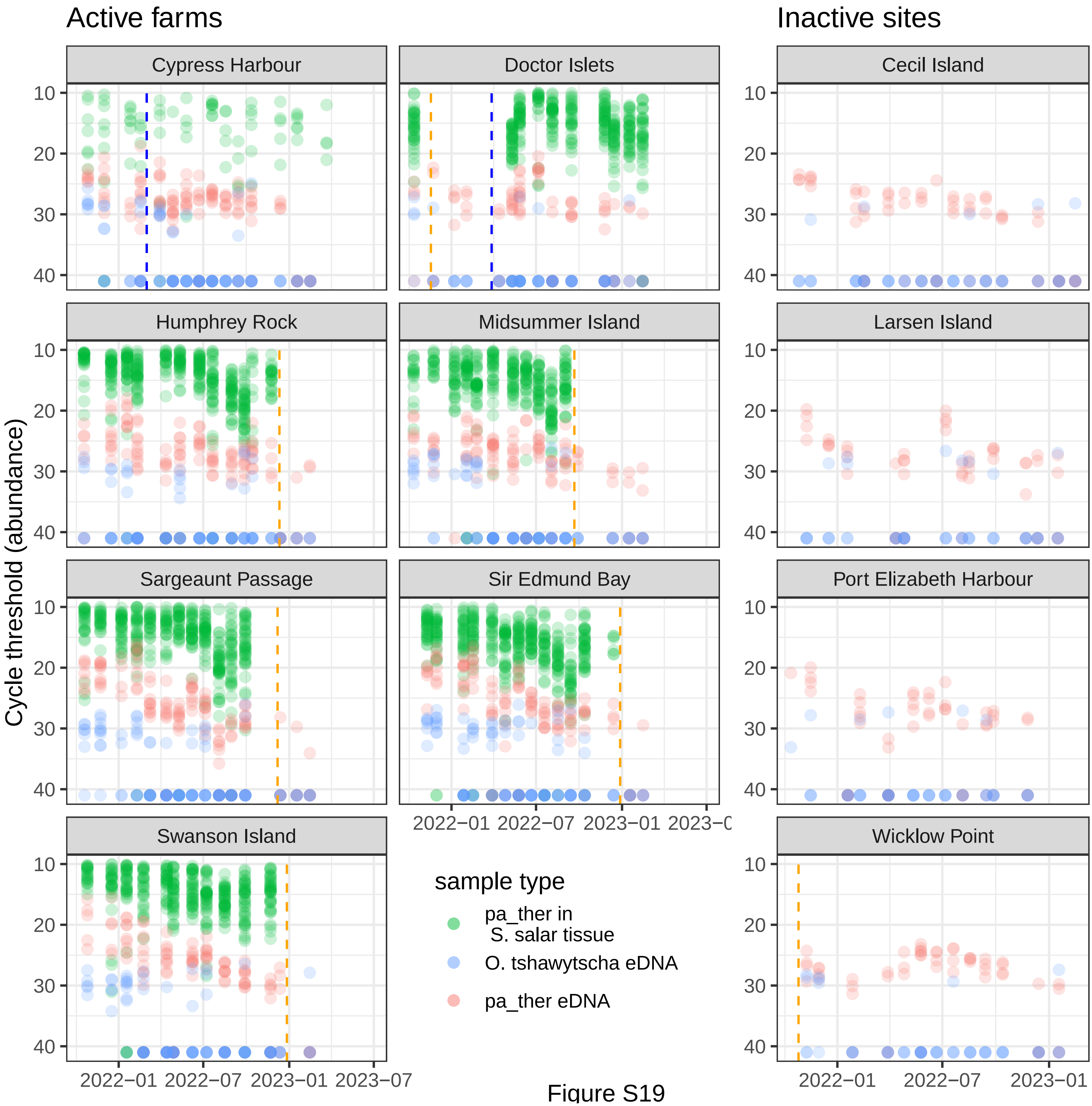

Active farms
Inactive sites
Cypress Harbour
Doctor Islets
Cecil Island
Humphrey Rock
Midsummer Island
Larsen Island
Sargeaunt Passage
Sir Edmund Bay
Port Elizabeth Harbour
Swanson Island
Wicklow Point
Cycle threshold (abundance)
10
20
30
40
2022−01
2022−07
2023−01
2023−07
sample type
pa_ther in S. salar tissue
O. tshawytscha eDNA
pa_ther eDNA

Figure S19

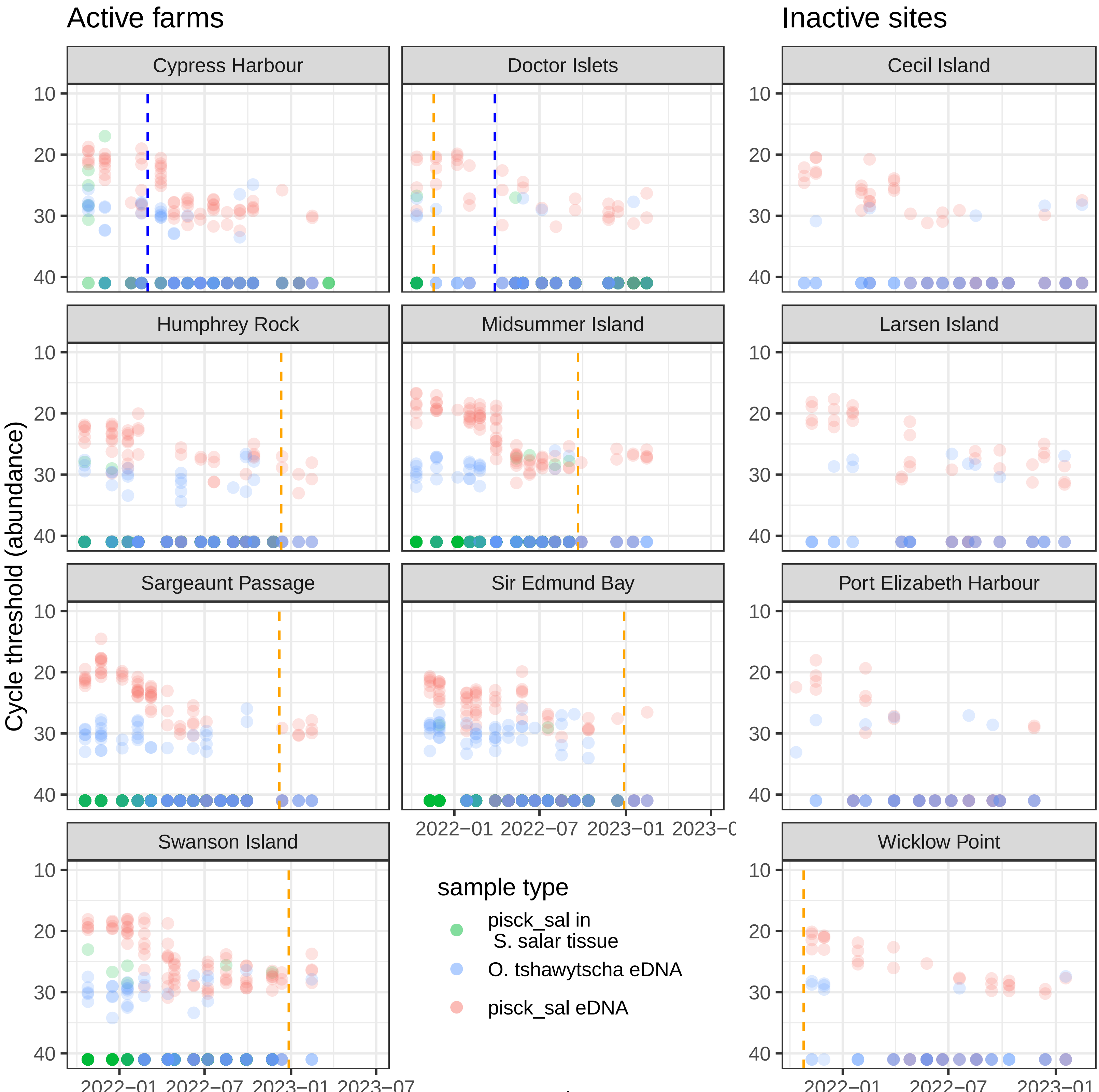

Active farms
Inactive sites
Cypress Harbour
Doctor Islets
Cecil Island
Humphrey Rock
Midsummer Island
Larsen Island
Sargeaunt Passage
Sir Edmund Bay
Port Elizabeth Harbour
Swanson Island
Wicklow Point
Cycle threshold (abundance)
10
20
30
40
2022−01
2022−07
2023−01
2023−07
sample type
pisck_sal in S. salar tissue
O. tshawytscha eDNA
pisck_sal eDNA

Figure S20

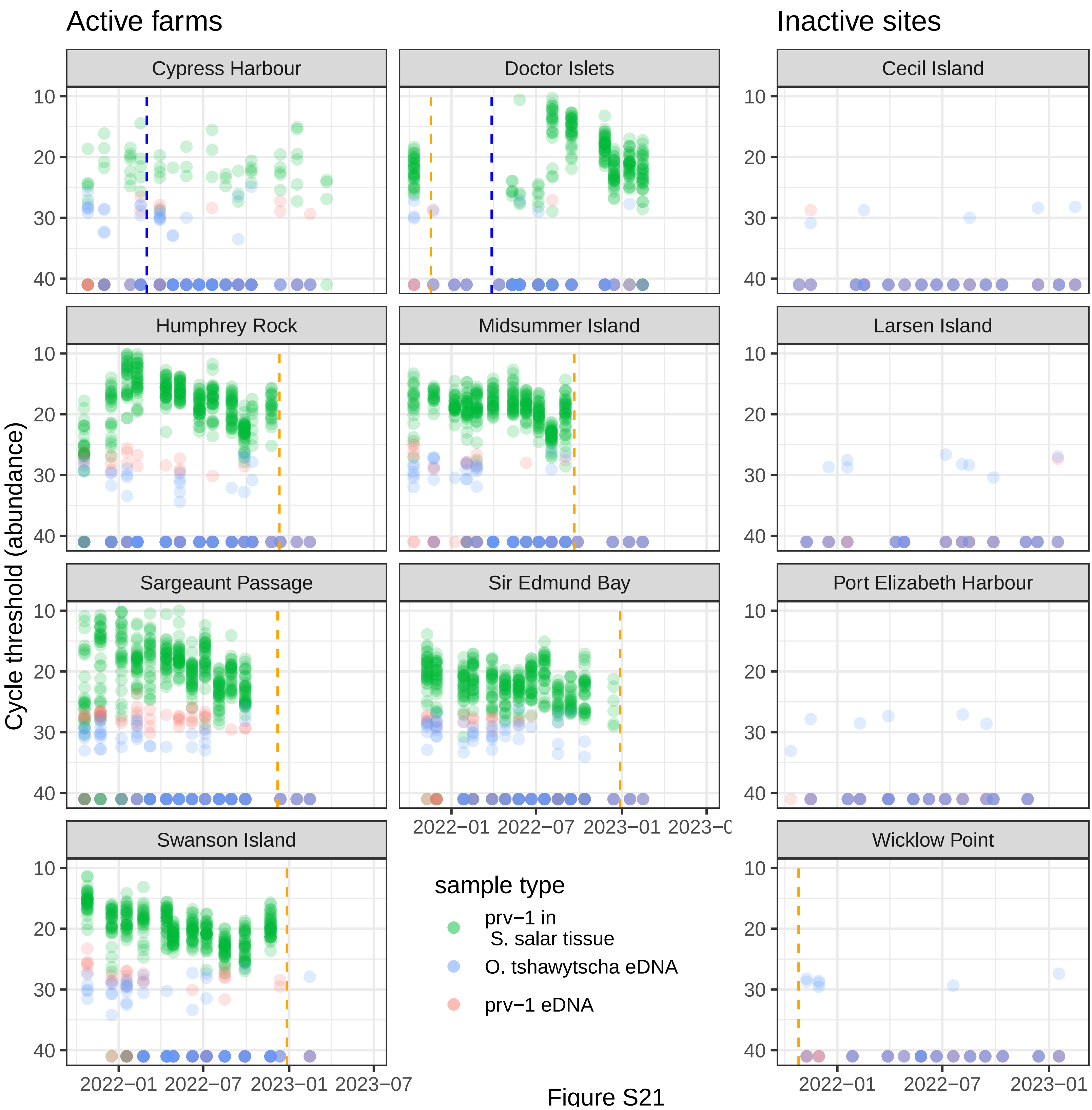

Active farms
Inactive sites
Cypress Harbour
Doctor Islets
Cecil Island
Humphrey Rock
Midsummer Island
Larsen Island
Sargeaunt Passage
Sir Edmund Bay
Port Elizabeth Harbour
Swanson Island
Wicklow Point
Cycle threshold (abundance)
10
20
30
40
2022−01
2022−07
2023−01
2023−07
sample type
prv−1 in S. salar tissue
O. tshawytscha eDNA
prv−1 eDNA

Figure S21

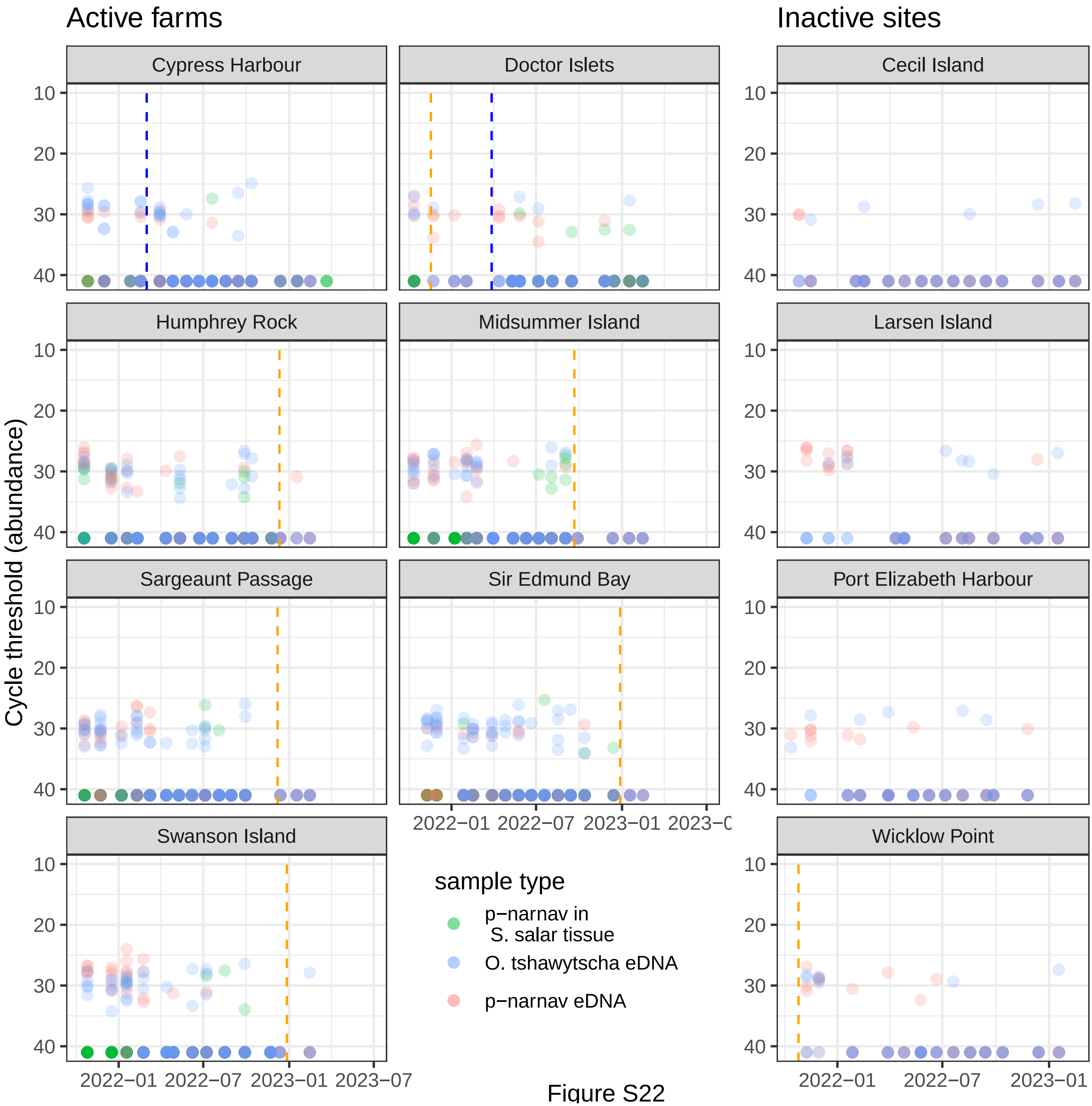

Active farms
Inactive sites
Cypress Harbour
Doctor Islets
Cecil Island
Humphrey Rock
Midsummer Island
Larsen Island
Sargeaunt Passage
Sir Edmund Bay
Port Elizabeth Harbour
Swanson Island
Wicklow Point
Cycle threshold (abundance)
10
20
30
40
2022−01
2022−07
2023−01
2023−07
sample type
p−narnav in S. salar tissue
O. tshawytscha eDNA
p−narnav eDNA

Figure S22

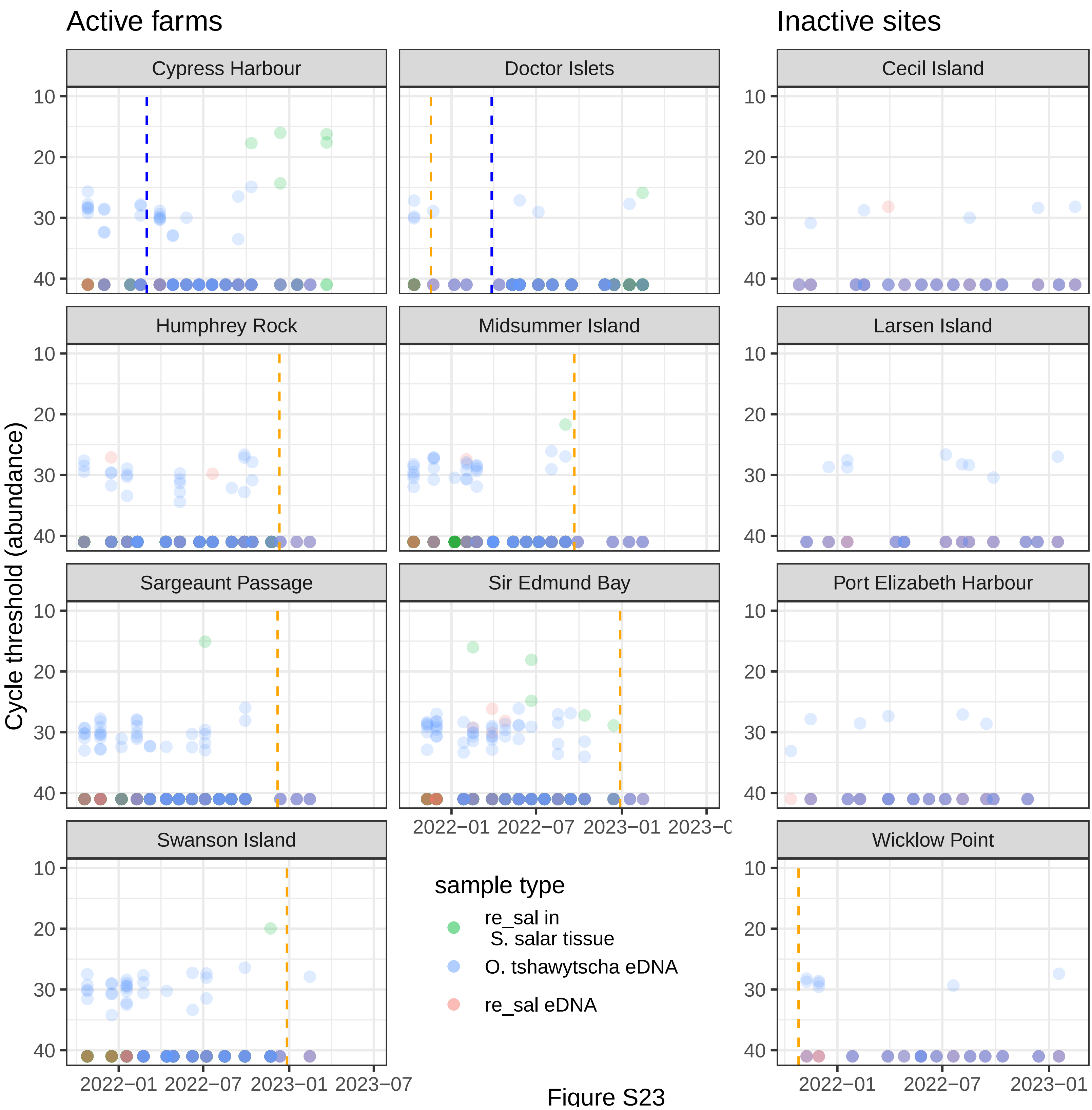
Active farms
Inactive sites
Cypress Harbour
Doctor Islets
Cecil Island
Humphrey Rock
Midsummer Island
Larsen Island
Sargeaunt Passage
Sir Edmund Bay
Port Elizabeth Harbour
Swanson Island
Wicklow Point
Cycle threshold (abundance)
10
20
30
40
2022−01
2022−07
2023−01
2023−07
sample type
re_sal in S. salar tissue
O. tshawytscha eDNA
re_sal eDNA

Figure S23

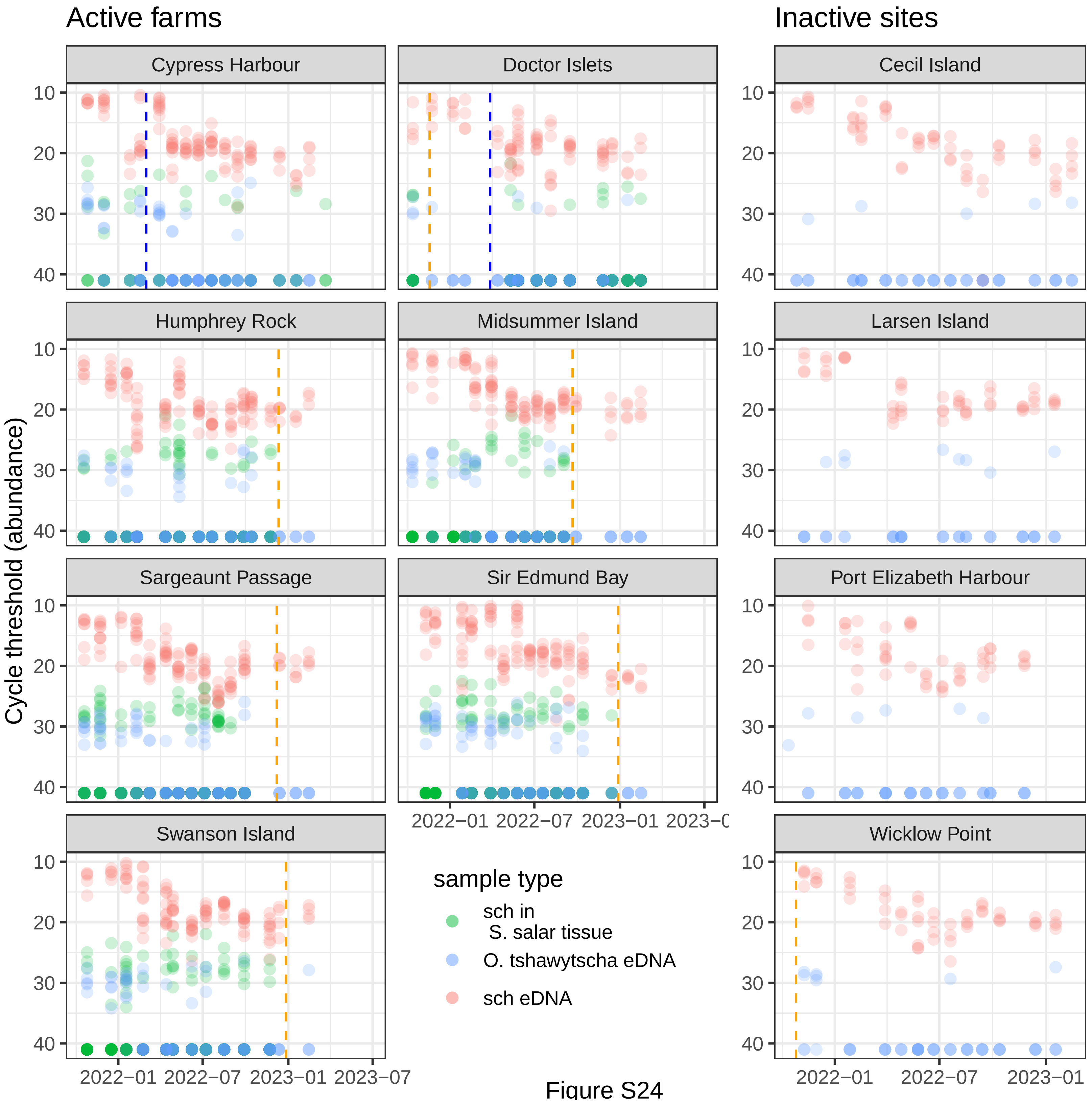
Active farms
Inactive sites
Cypress Harbour
Doctor Islets
Cecil Island
Humphrey Rock
Midsummer Island
Larsen Island
Sargeaunt Passage
Sir Edmund Bay
Port Elizabeth Harbour
Swanson Island
Wicklow Point
Cycle threshold (abundance)
10
20
30
40
2022−01
2022−07
2023−01
2023−07
sample type
sch in S. salar tissue
O. tshawytscha eDNA
sch eDNA

Figure S24

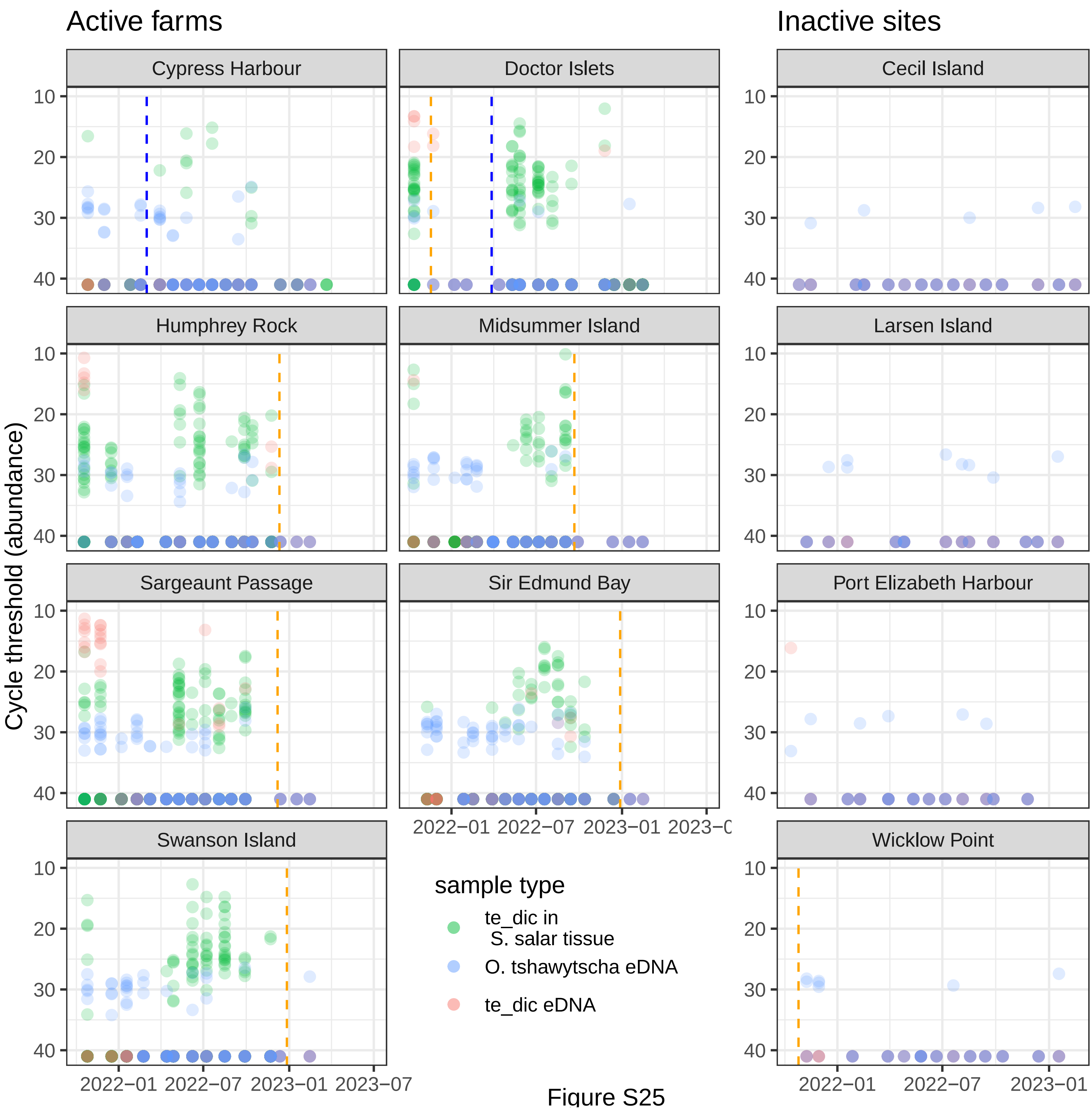
Active farms
Inactive sites
Cypress Harbour
Doctor Islets
Cecil Island
Humphrey Rock
Midsummer Island
Larsen Island
Sargeaunt Passage
Sir Edmund Bay
Port Elizabeth Harbour
Swanson Island
Wicklow Point
Cycle threshold (abundance)
10
20
30
40
2022−01
2022−07
2023−01
2023−07
sample type
te_dic in
S. salar tissue
O. tshawytscha eDNA
te_dic eDNA

Figure S25

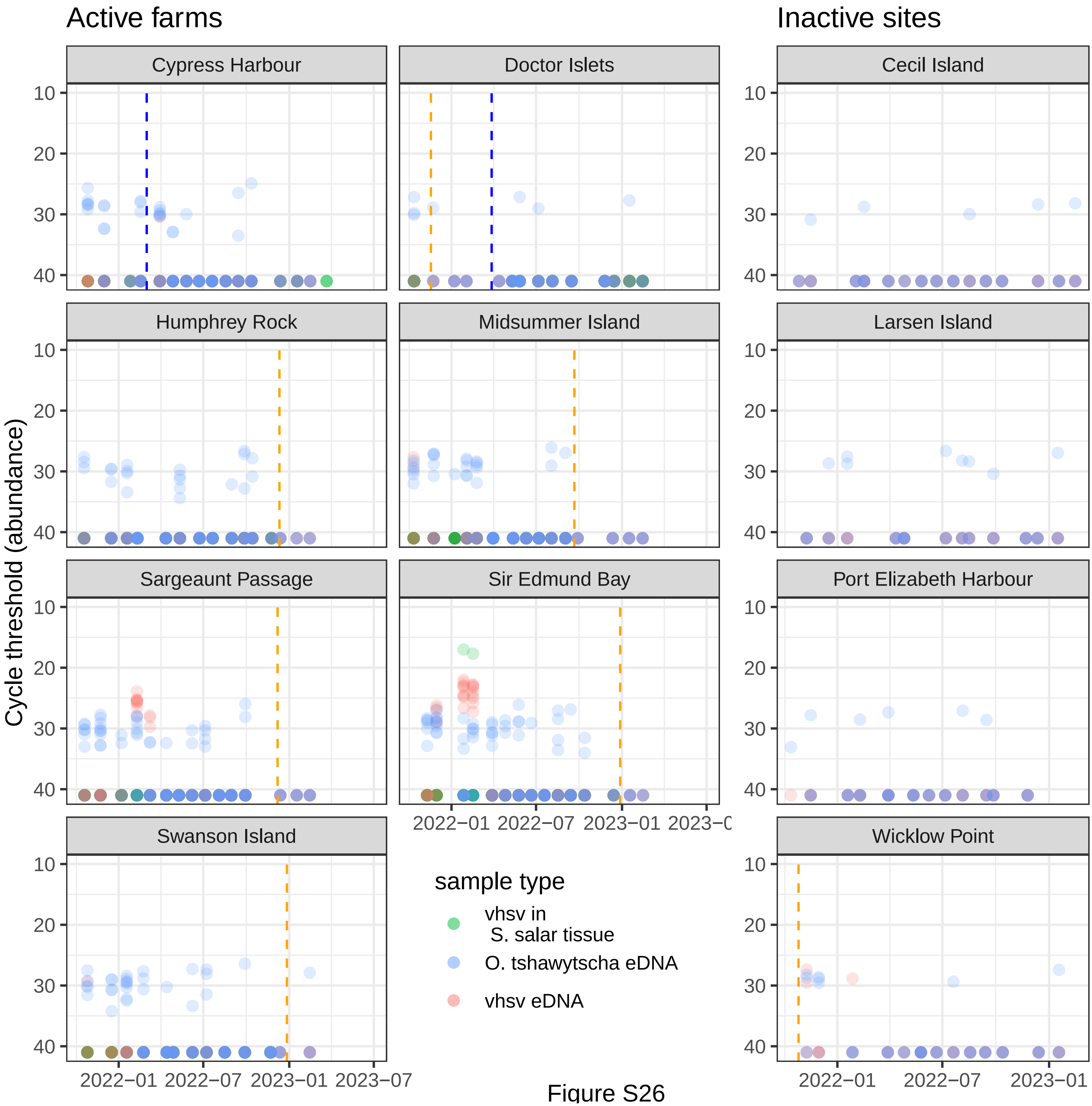
Active farms
Inactive sites
Cypress Harbour
Doctor Islets
Cecil Island
Humphrey Rock
Midsummer Island
Larsen Island
Sargeaunt Passage
Sir Edmund Bay
Port Elizabeth Harbour
Swanson Island
Wicklow Point
Cycle threshold (abundance)
10
20
30
40
2022−01
2022−07
2023−01
2023−07
sample type
vhsv in
S. salar tissue
O. tshawytscha eDNA
vhsv eDNA

Figure S26

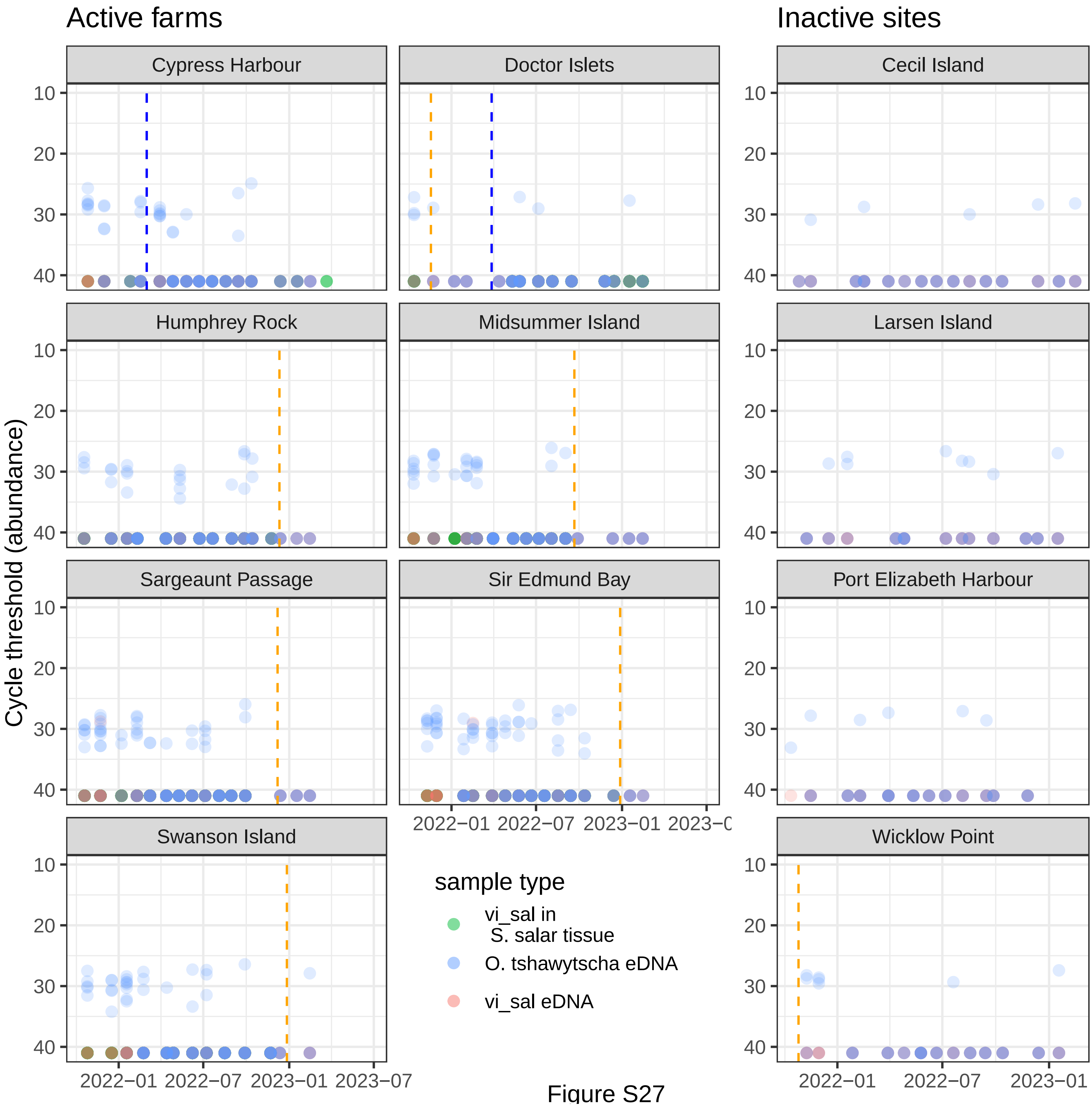

Active farms
Inactive sites
Cypress Harbour
Doctor Islets
Cecil Island
Humphrey Rock
Midsummer Island
Larsen Island
Sargeaunt Passage
Sir Edmund Bay
Port Elizabeth Harbour
Swanson Island
Wicklow Point
Cycle threshold (abundance)
10
20
30
40
2022−01
2022−07
2023−01
2023−07
sample type
vi_sal in S. salar tissue
O. tshawytscha eDNA
vi_sal eDNA

Figure S27

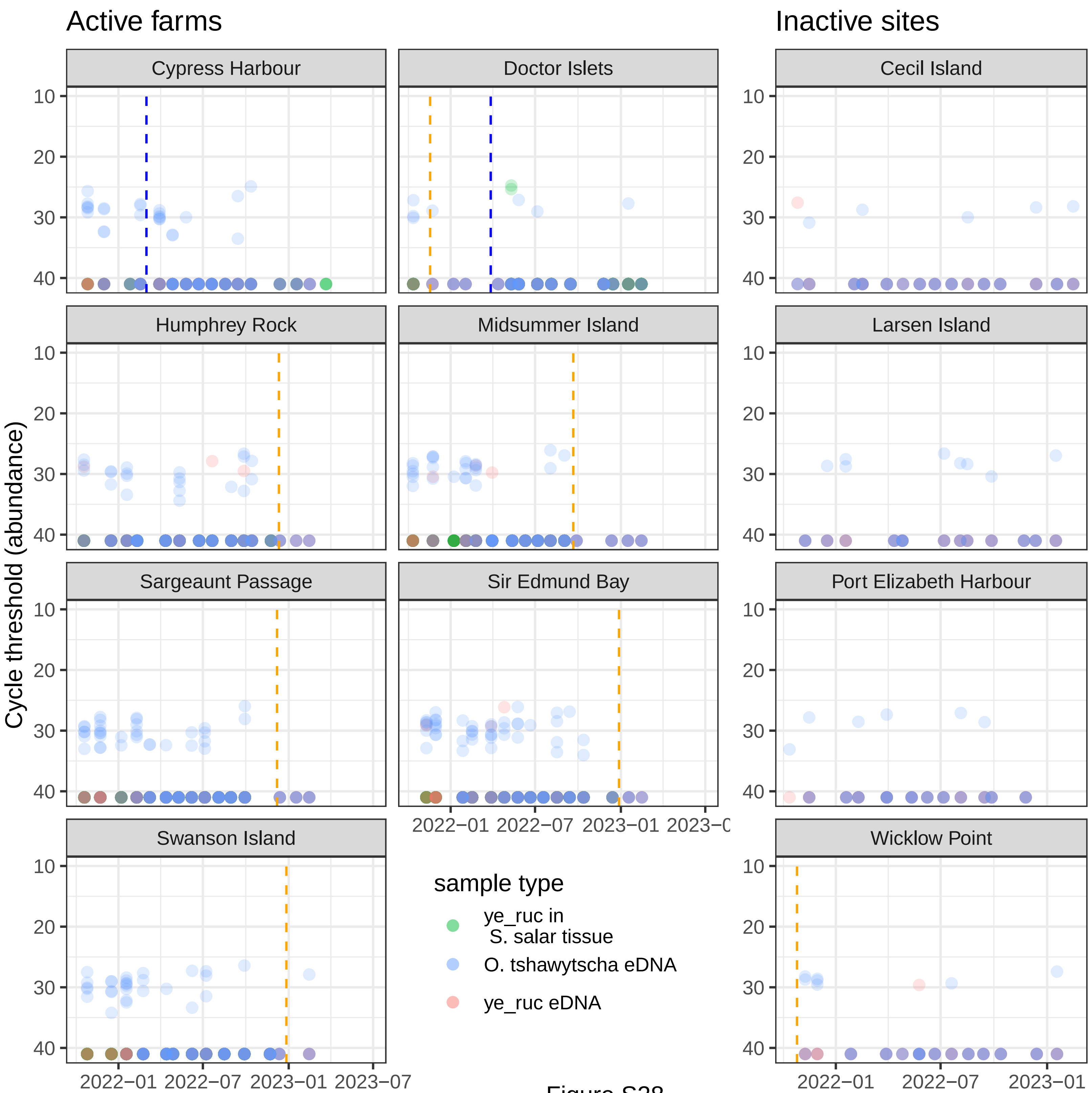
Active farms
Inactive sites
Cypress Harbour
Doctor Islets
Cecil Island
Humphrey Rock
Midsummer Island
Larsen Island
Sargeaunt Passage
Sir Edmund Bay
Port Elizabeth Harbour
Swanson Island
Wicklow Point
Cycle threshold (abundance)
10
20
30
40
2022−01
2022−07
2023−01
2023−07
sample type
ye_ruc in S. salar tissue
O. tshawytscha eDNA
ye_ruc eDNA

Figure S28

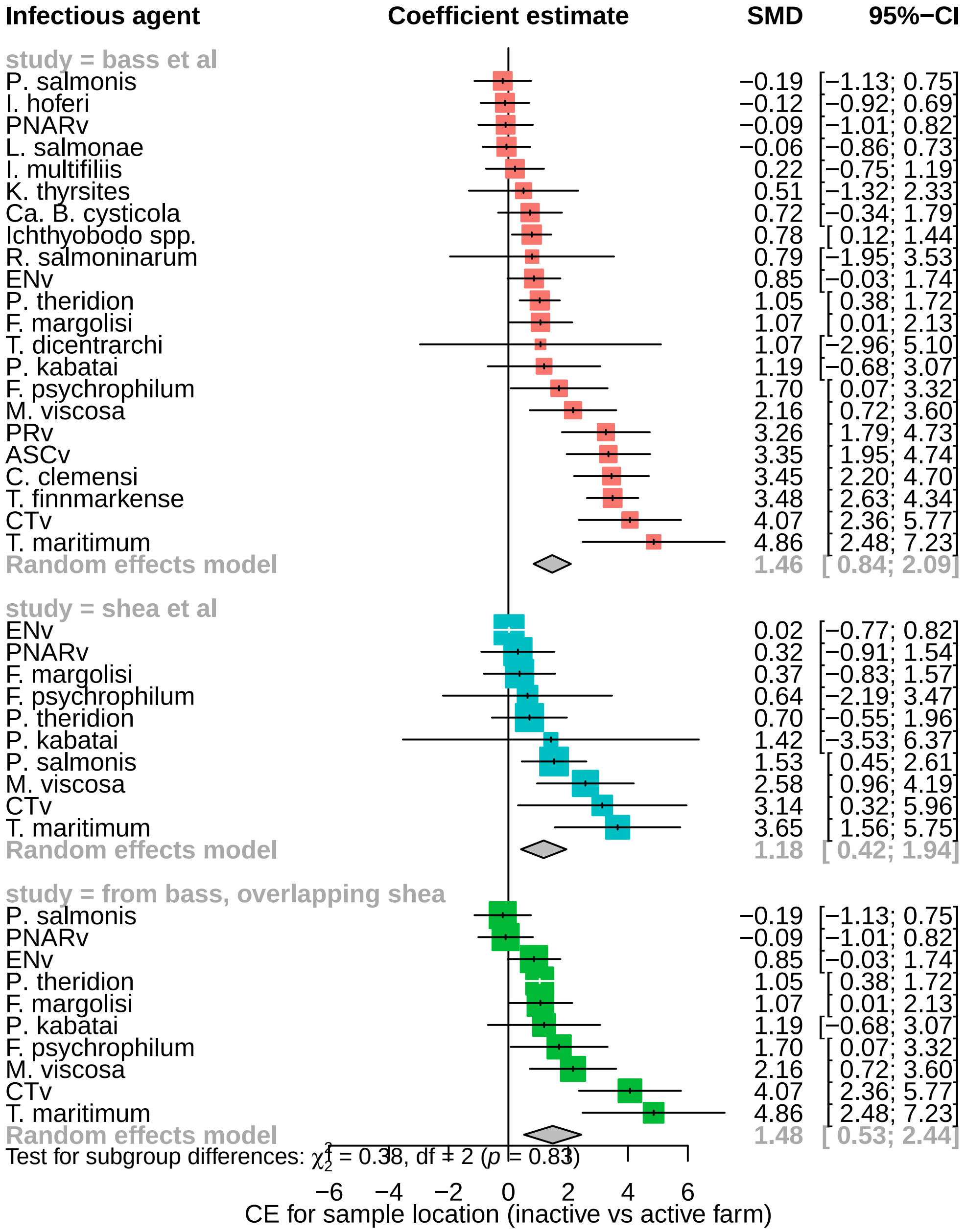

Figure S29: In both this study and Shea *et al.* 2020, meta-analytic means indicated that as a group, infectious agents assayed in eDNA samples were more likely to be detected around active netpen aquaculture than inactive sites. Model results are grouped by study, with a third group displaying only those pathogens from this study that overlap with those we could model from Shea *et al.* 2020. Diamonds indicate the mean and 95% confidence intervals for pathogens from each study. Horizontal black lines represent 95% confidence intervals around coefficient estimates.

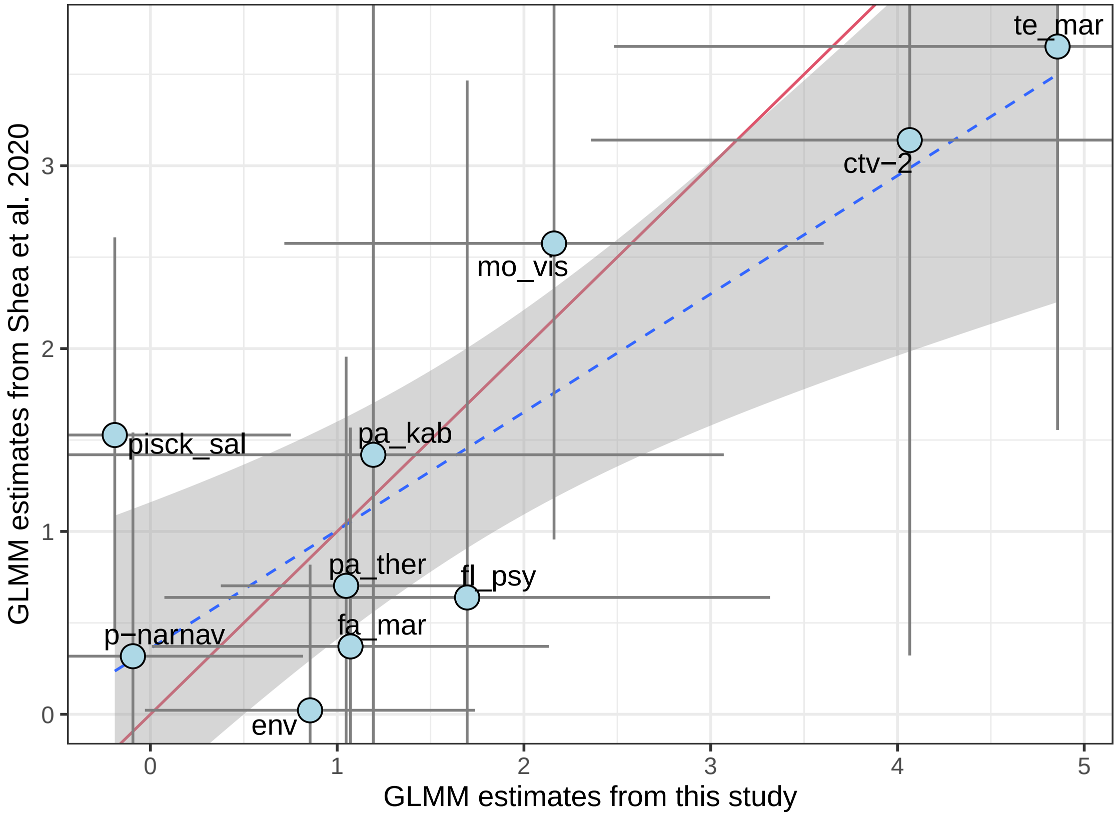

Figure S30: GLMM model estimates from this study were similar ($R^2 = 0.65$) to those for the same infectious agents using data from Shea *et al.* 2020. Error bars around each point indicate the 95% confidence intervals for the estimates from the GLMM models. The dotted blue line indicates the model fit and the gray shaded area is the 95% confidence interval around the fit. The red line indicates a slope of 1.

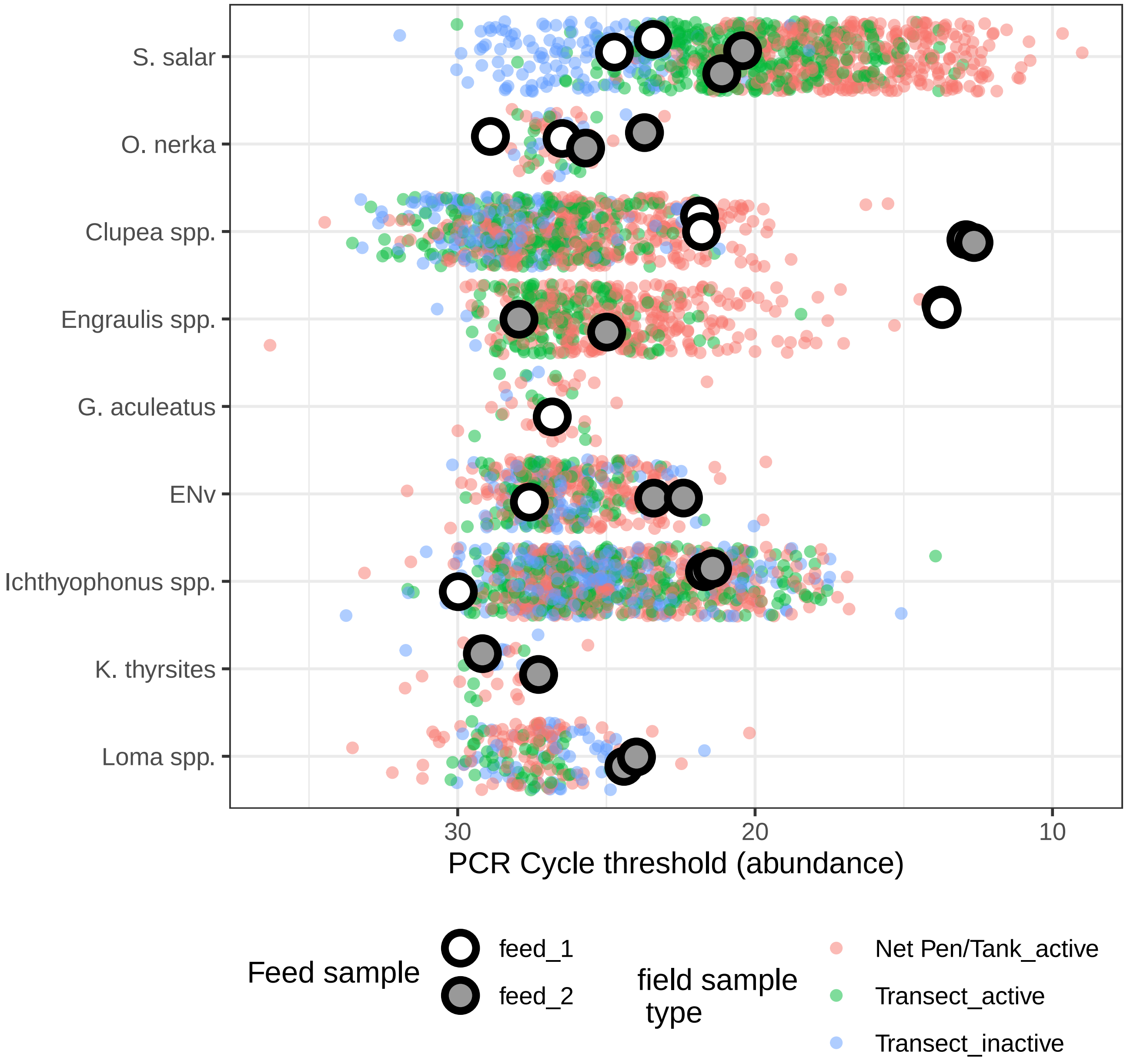

Figure S31: Salmonid, marine fish, and pathogen nucleic acids targeted by our assay panel were detected in two samples of pelleted salmon feed. Color points show PCR detections in water samples collected at active (within and around netpens) and inactive farms. Large, gray and white circles represent duplicate analyses of two feed samples. All points are jittered vertically for display. Lower cycle threshold values (left) indicate greater abundance of nucleic acids.

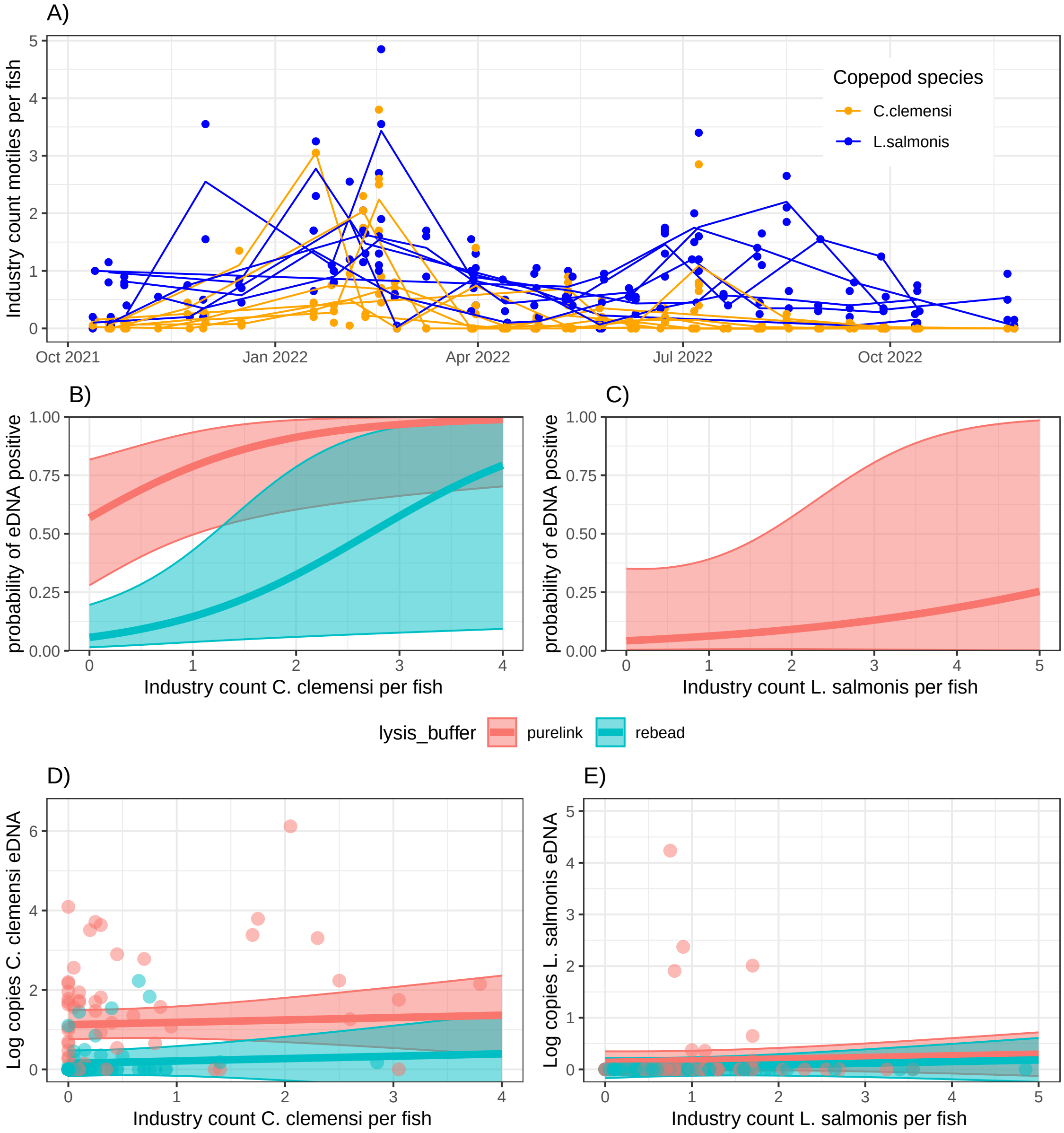

Figure S32: Despite higher aquaculture industry counts of *L. salmonis* than *C. caligus* (panel A), *C. caligus* eDNA was more frequently detected and showed a positive association between industry counts and likelihood of detection (panel B). Panel A shows netpen-specific industry counts (points) over the study period for each species with monthly farm-specific averages linked by lines. Panels B and C show the model fit for a binomial GLMM testing the association between industry counts and likelihood of detection in eDNA, split by lysis buffer (red=purelink, blue=rebead). Due to no detections with the rebead lysis buffer, a fit could not be created for *L. salmonis* (C). Panels D and E show raw data and LMM fits for tests of correlations between industry counts and eDNA RNA/DNA log copy number.
